# Enantioselective catalytic β-amination through proton-coupled electron transfer followed by stereocontrolled radical–radical coupling†
†Electronic supplementary information (ESI) available. See DOI: 10.1039/c7sc02031g



**DOI:** 10.1039/c7sc02031g

**Published:** 2017-06-15

**Authors:** Zijun Zhou, Yanjun Li, Bowen Han, Lei Gong, Eric Meggers

**Affiliations:** a College of Chemistry and Chemical Engineering , Xiamen University , Xiamen 361005 , P. R. China . Email: gongl@xmu.edu.cn; b Fachbereich Chemie , Philipps-Universität Marburg , Hans-Meerwein-Strasse 4 , 35043 Marburg , Germany . Email: meggers@chemie.uni-marburg.de

## Abstract

A new mechanistic approach for the catalytic, enantioselective conjugate addition of nitrogen-based nucleophiles to acceptor-substituted alkenes is reported, which is based on a visible light induced and phosphate base promoted transfer of a single electron from a nitrogen nucleophile to a catalyst-bound acceptor-substituted alkene, followed by a stereocontrolled C–N bond formation through stereocontrolled radical–radical coupling. Specifically, *N*-aryl carbamates are added to the β-position of α,β-unsaturated 2-acyl imidazoles using a visible light activated photoredox mediator in combination with a chiral-at-rhodium Lewis acid catalyst and a weak phosphate base, affording new C–N bonds in a highly enantioselective fashion with enantioselectivities reaching up to 99% ee and >99 : 1 dr for a menthol-derived carbamate. As an application, the straightforward synthesis of a chiral β-amino acid ester derivative is demonstrated.

## Abstract 2

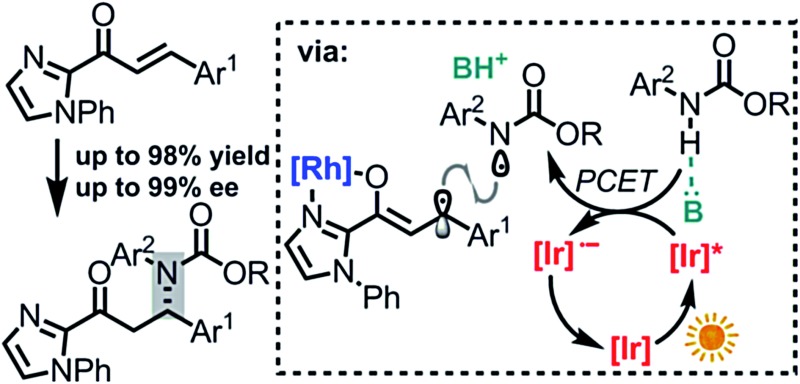
The catalytic asymmetry conjugate addition of carbamates to α,β-unsaturated 2-acyl imidazoles is accomplished using visible-light-induced proton-coupled electron transfer.

## 

Chiral amines featuring a stereogenic carbon connected to a nitrogen are important structural motifs in pharmaceuticals and natural products.^1^ The development of methodology for the catalytic, asymmetric construction of C–N bonds is therefore of high relevance.^2^ In this respect, the conjugate amination of readily available α,β-unsaturated carbonyl compounds is a principal strategy which provides useful β-amino carbonyl building blocks and exploits the intrinsic nucleophilicity of nitrogen lone pairs, with or without a prior deprotonation of the N–H group (Fig. 1).^3,4^ However, the high nucleophilicity of such nitrogen reagents needs to be controlled carefully in order to prevent side reactions, catalyst deactivation, and uncatalyzed background reactions reducing the enantioselectivity.^1^

**Fig. 1:**
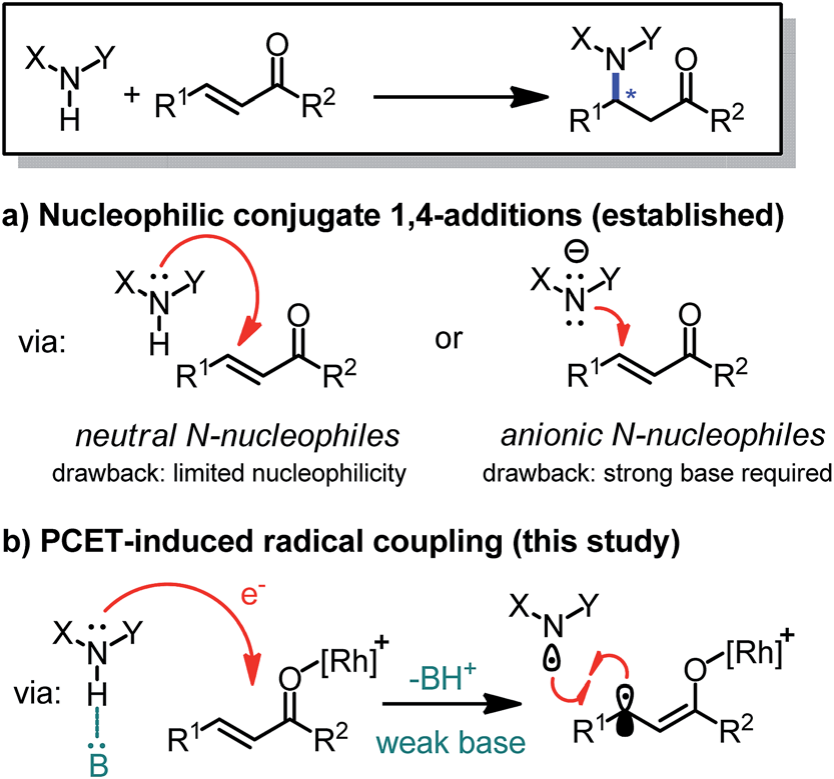
Formation of C–N bonds by β-amination of readily available α,β-unsaturated carbonyl compounds. Scope limitations for the PCET-induced radical coupling reported in this study: X = aryl, Y = CO_2_R, R^1^ = aryl, R^2^ = 2-imidazolyl.

Recently, Knowles introduced an elegant strategy making use of proton-coupled electron transfer (PCET) to convert N–H groups into nitrogen-centered radicals, whose high reactivities can then be exploited for radical additions to electron-rich double bonds or hydrogen atom transfer reactions.^5–8^ The activation of N–H containing compounds by PCET is attractive for its mild reaction conditions: it requires only a weak base, such as a phosphate, and the single electron transfer can be induced by visible light using a photoredox mediator. Unfortunately, the electron-deficient nature of nitrogen-centered radicals renders their application to the direct radical β-amination of unsaturated carbonyl compounds unfavorable, at least for intermolecular reactions.^9–11^

Herein, we introduce a novel strategy to interface photoinduced PCET with the catalytic, enantioselective conjugate amination of α,β-unsaturated carbonyl compounds. The mild and visible light induced enantioselective C–N bond formation is catalyzed by a previously developed chiral-at-rhodium Lewis acid^12^ and couples *N*-aryl carbamates with α,β-unsaturated 2-acyl imidazoles with high yields and excellent enantioselectivities.

We commenced our study by investigating the reaction of the α,β-unsaturated 2-acyl imidazole **1a** with *N*-phenyl carbamic acid methyl ester **2a** under PCET conditions^8^ using the iridium-based photoredox mediator **PC1** ^13^ and the weak phosphate base **B1**, and combined this with the chiral-at-rhodium Lewis acid Δ-**RhO** ^14^ (Table 1). Encouragingly, upon irradiation with blue LEDs at room temperature for 16 hours we observed a conversion of 43% to the C–N bond formation product (*S*)-**3a** with 96% ee (entry 1). Changing the solvent from CHCl_3_ to CH_2_Cl_2_ and slightly elongating the reaction time provided full conversion while retaining the high enantioselectivity (entry 2). The enantiomeric excess could be further improved to 98% ee upon optimization of the phosphate base (**B2** and **B3**, entries 3 and 4). Examining other photoredox mediators provided inferior results (entries 5 and 6) and it is also worth noting that the related chiral-at-metal catalysts Δ-**RhS** ^15^ (entry 7) and Δ-**IrO** ^16^ (entry 8) are not suitable for this method. Meanwhile, control experiments verified that Δ-**RhO**, a phosphate base (**B2**), and blue light are all essential for product formation (entries 9–11). Interestingly, product is formed with high enantioselectivity even in the absence of the photoredox mediator, but the conversion is more sluggish and cannot be brought to full completion even after elongated reaction times (entry 12).

**Table 1:** Optimization of the reaction conditions^*a*^

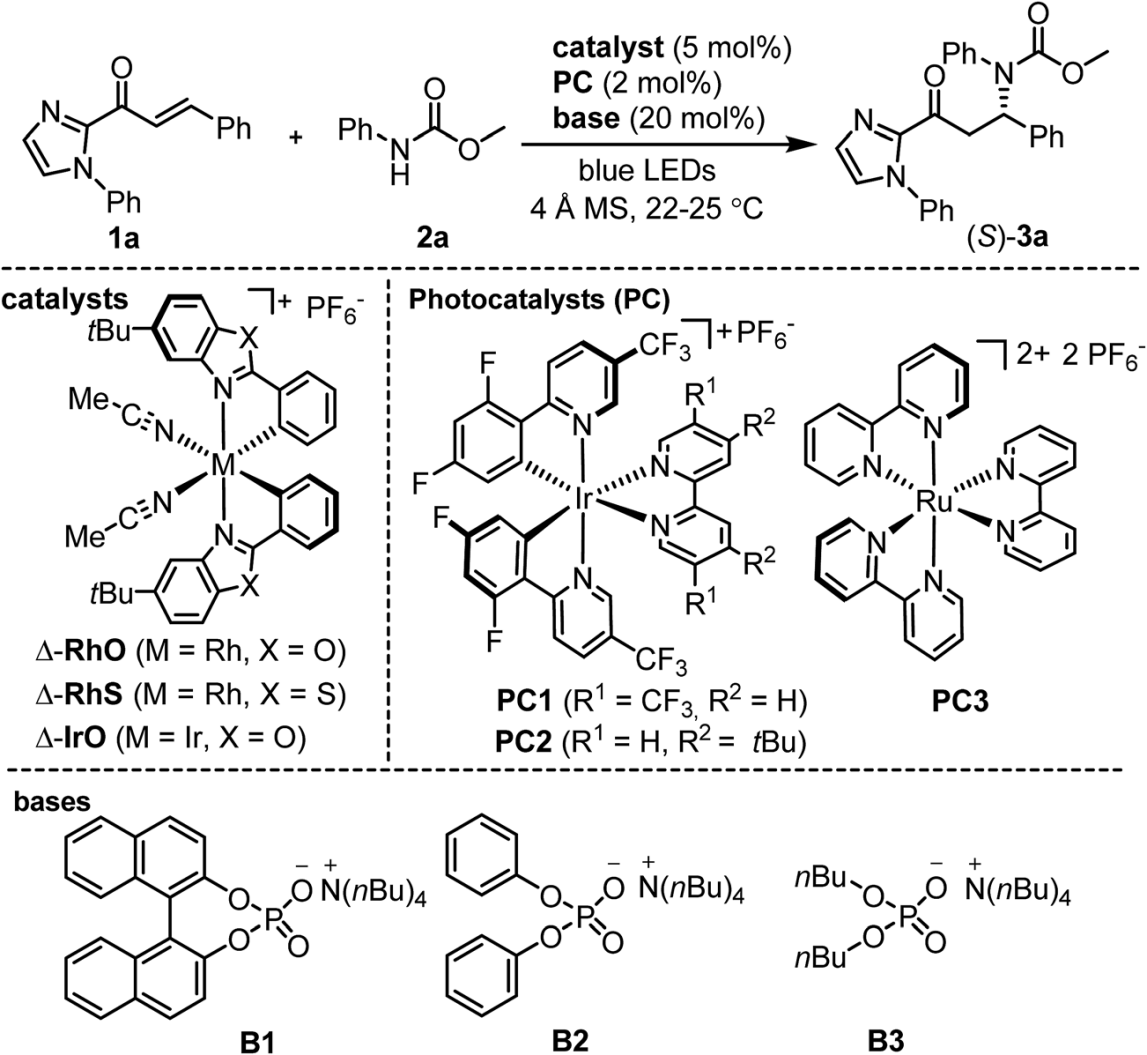							
Entry	Catalyst	Base	PC	*hν*	Solvent	Yield^*b*^ (%)	ee^*c*^ (%)
1	Δ-**RhO**	**B1**	**PC1**	Light	CHCl_3_	43	96
2	Δ-**RhO**	**B1**	**PC1**	Light	CH_2_Cl_2_	Quant.	96
3	Δ-**RhO**	**B2**	**PC1**	Light	CH_2_Cl_2_	Quant.	98
4	Δ-**RhO**	**B3**	**PC1**	Light	CH_2_Cl_2_	24	74
5	Δ-**RhO**	**B2**	**PC2**	Light	CH_2_Cl_2_	41	97
6	Δ-**RhO**	**B2**	**PC3**	Light	CH_2_Cl_2_	34	98
7	Δ-**RhS**	**B2**	**PC1**	Light	CH_2_Cl_2_	<10	n.d.^*d*^
8	Δ-**IrO**	**B2**	**PC1**	Light	CH_2_Cl_2_	Trace	n.d.^*d*^
9	None	**B2**	**PC1**	Light	CH_2_Cl_2_	0^*e*^	n.a.^*f*^
10	Δ-**RhO**	None	**PC1**	Light	CH_2_Cl_2_	8	93
11	Δ-**RhO**	**B2**	**PC1**	Dark	CH_2_Cl_2_	0	n.a.^*f*^
12	Δ-**RhO**	**B2**	None	Light	CH_2_Cl_2_	39	98

^*a*^Reaction conditions: α,β-unsaturated acyl imidazole **1a** (0.10 mmol), carbamate **2a** (0.12 mmol), metal catalyst (0.005 mmol), **PC1-3** (0.002 mmol), **B1-3** (0.02 mmol), 4 Å molecular sieves (MS) (10 mg), and solvent (1.0 mL). The reactions were irradiated with blue LEDs (24 + 36 W), except for entry 11.

^*b*^Yields determined by crude ^1^H NMR analysis.

^*c*^Ee values determined by HPLC analysis on a chiral stationary phase.

^*d*^Not determined.

^*e*^Cyclobutane side product formed.

^*f*^Not applicable.

The proposed mechanism is shown in Fig. 2. The photoactivated photoredox mediator (PC*) initiates a proton-coupled electron transfer (PCET) involving the Brønsted-base-activated carbamate to generate a nitrogen-centered radical and a reduced photoredox mediator (PC˙^–^), which in turn transfers an electron to the Rh-coordinated substrate (**I** → **II**). Thus, the photoredox mediator serves as a single electron shuttle from the carbamate to the Rh-bound α,β-unsaturated 2-acyl imidazole, providing an electron deficient carbamoyl *N*-radical and the persistent electron rich rhodium enolate radical intermediate **II**,^17,18^ which subsequently recombines to the rhodium enolate intermediate **III**.^19^ After protonation by the protonated phosphate base (**III** → **IV**), product release, and coordination of new substrate (**IV** → **I**), a new catalytic cycle can be initiated. The function of the rhodium catalyst in this mechanism is twofold. First, the established *N*,*O*-bidentate coordination^12^ to the unsaturated 2-acyl imidazole facilitates the reduction of the Rh-bound substrate. Secondly, the chiral Lewis acid controls the stereochemistry of the radical–radical coupling by providing a high asymmetric induction. Note that in this radical–radical coupling step the stereocontrol occurs through controlling the reaction of a catalyst-bound prochiral carbon-centered radical.^20^

**Fig. 2:**
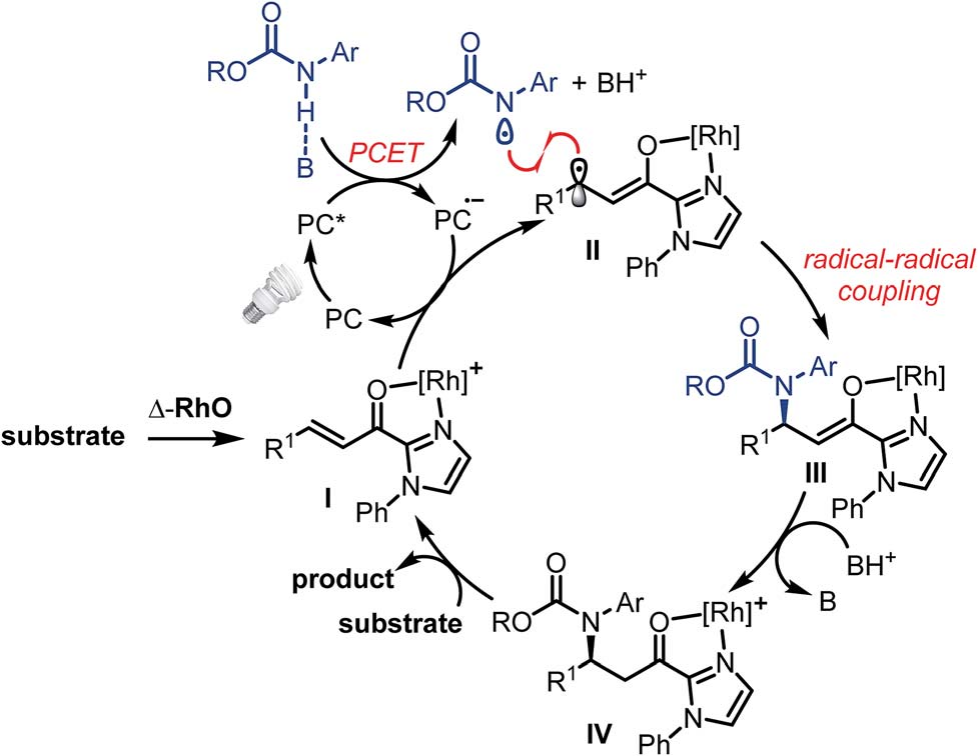
Proposed mechanism.

A number of control experiments back this mechanism. First, visible light, rhodium catalyst, and phosphate base are all required for product formation. Second, intermediate *N*-centered radical formation was verified by trapping the nitrogen-centered radical by an intramolecular radical addition to an alkene (**2b**) followed by reaction of the formed carbon-centered radical with the unsaturated 2-acyl imidazole **1a**, affording the product **4** with 89% yield and 97% ee (Fig. 3a). Third, in the absence of any Rh-catalyst, the cyclobutane complex **5** is formed as the major product in 36% yield. This reaction is analogous to Yoon's reported photoredox-mediated [2 + 2] cycloaddition involving unsaturated 2-acyl imidazoles which was proposed to proceed through the single electron reduction of the unsaturated 2-acyl imidazoles and supports the involvement of the Rh-enolate radical intermediate **II** in our pathway.^18,21^ Finally, when we added one equivalent of TEMPO to the reaction, no product was formed which is indicative of a radical mechanism.

**Fig. 3:**
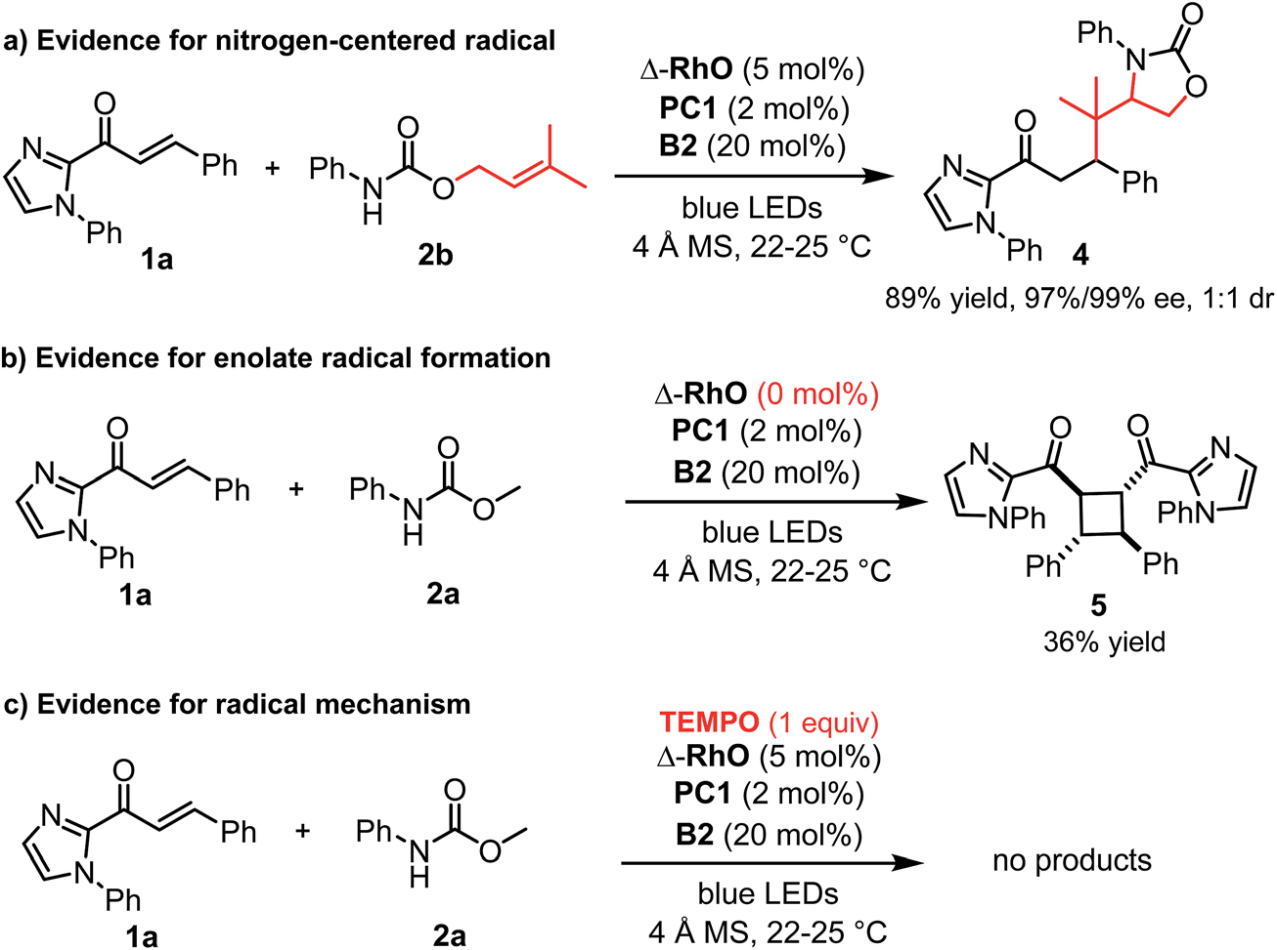
Control experiments.

Alternative mechanistic scenarios through the addition of *N*-carbamoyl radicals or carbamate anions to the rhodium-coordinated α,β-unsaturated 2-acyl imidazoles are unlikely and are briefly discussed. *N*-centered carbamoyl and amidyl radicals are classified as electrophilic radicals which prefer to react with electron rich alkenes.^11,22,23^ Rhodium coordination to the α,β-unsaturated 2-acyl imidazole substrates would further decrease the radical addition rate and is therefore not consistent with the observed high enantioselectivity under rhodium catalysis. On the other hand, a mechanism through the conjugate addition of carbamate anions is unlikely due to the requirement for light activation and the experimental evidence for the intermediate formation of a carbamoyl radical (Fig. 3a). As an additional control experiment we performed the standard reaction in the presence of the strong base sodium ethoxide instead of the weak phosphate base but no product was formed in the absence of light (see ESI for details†). Furthermore, the reduction of an intermediate carbamoyl radical to the anion (by the reduced photoredox mediator) followed by a conjugate addition is unlikely because the reaction also proceeds in the absence of an additional photoredox mediator with reduced yields but identical enantioselectivity (Table 1, entry 12).

This proposed mechanism resembles some conceptional similarity with Melchiorre's recently reported visible-light-excited enantioselective β-alkylation of α,β-unsaturated aldehydes catalyzed by a chiral secondary amine.^20*g*^ A photoactivated conjugated iminium ion intermediate oxidizes the alkylsilane co-substrate by single electron transfer followed by a radical–radical recombination. The iminium ion in Melchiorre's system resembles our rhodium-coordinated α,β-unsaturated 2-acyl imidazole (intermediate **I**), both of which serve as *in situ* assembled chiral single electron acceptors, accepting the single electron from a cosubstrate, followed by a stereocontrolled radical–radical recombination. Other photoactivated catalytic asymmetric reactions in which single electrons are transferred between a substrate-bound catalyst and a co-substrate have been reported.^20^

Next, after having investigated the mechanism of this novel catalytic, enantioselective C–N bond formation, we evaluated the scope (Fig. 4). The visible-light-induced enantioselective β-amination of **2a** to electron-deficient alkenes provided the C–N bond formation products (**3b–g**) with 82–97% yields and 94–99% ee. Different substituted aromatic moieties with respect to steric and electronic effects are well tolerated. With an electron-donating methoxy substituted aromatic moiety (product **3i**), the reaction time needed to be extended to 72 hours in order to achieve a high yield (87%) and high enantioselectivity (97% ee). However, when we applied this β-amination to a substrate with an *ortho*-methylated phenyl group, only traces of the product **3h** were detected. Apparently, the methyl group prevents a proper conjugation of the entire π-system, which suppresses the entire PCET mechanism. This also explains the requirement for an aromatic moiety in β-position of the α,β-unsaturated 2-acyl imidazole substrates as the formation of the product **3j** failed.

**Fig. 4:**
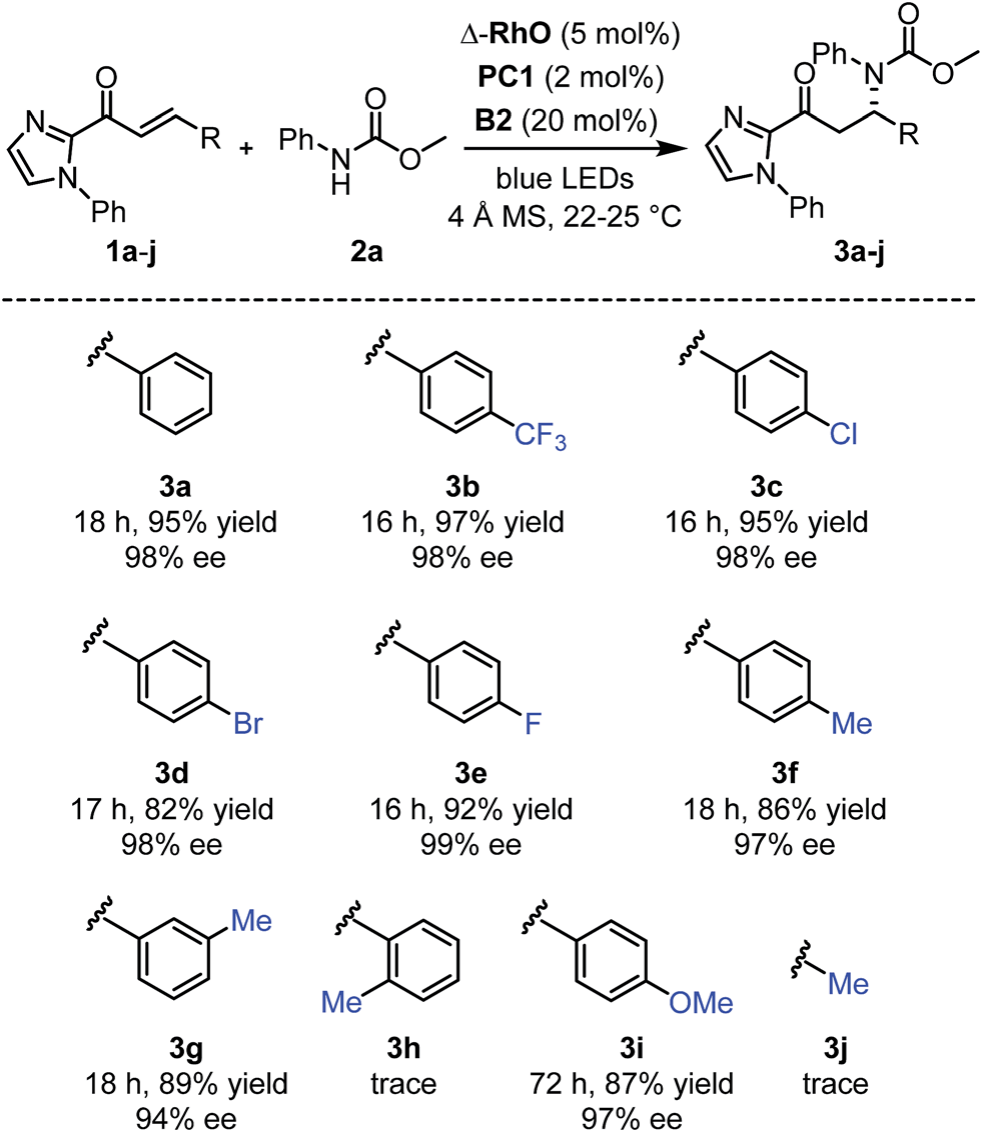
Reaction scope with regards to 2-acyl imidazoles.

The scope of this reaction with respect to substituted carbamates is outlined in Fig. 5. Different O-substituents such as ethyl, isopropyl, *tert*-butyl, and isobutyl (products **3k–n**) in the ester protected group are tolerated and provide high yields (87–98%) with excellent enantioselectivities (94–98% ee). Carbamates with *O*-benzyl or an *O*-phenethyl group (products **3o**, **p**) reacted more slowly. The *N-para*-methoxyphenyl (PMP) protection group was not tolerated and did not provide the β-amination product **3q** but instead a dimer of the 2-acyl imidazole substrate. However, methyl and halogen substituents at the *N*-phenyl moiety (products **3r–v**) are well tolerated with β-amination yields of 84–92% and 96–99% ee. Also, a menthol-derived carbamate provided the β-amination product **3w** in 85% yield with >99 : 1 d.r. As a limitation, the carbamate relies on *N*-aryl substituents (**3x**), and amides do not or almost not provide the desired products (**3y**, **z**) but instead the cyclobutane complex **5** as a side product.

**Fig. 5:**
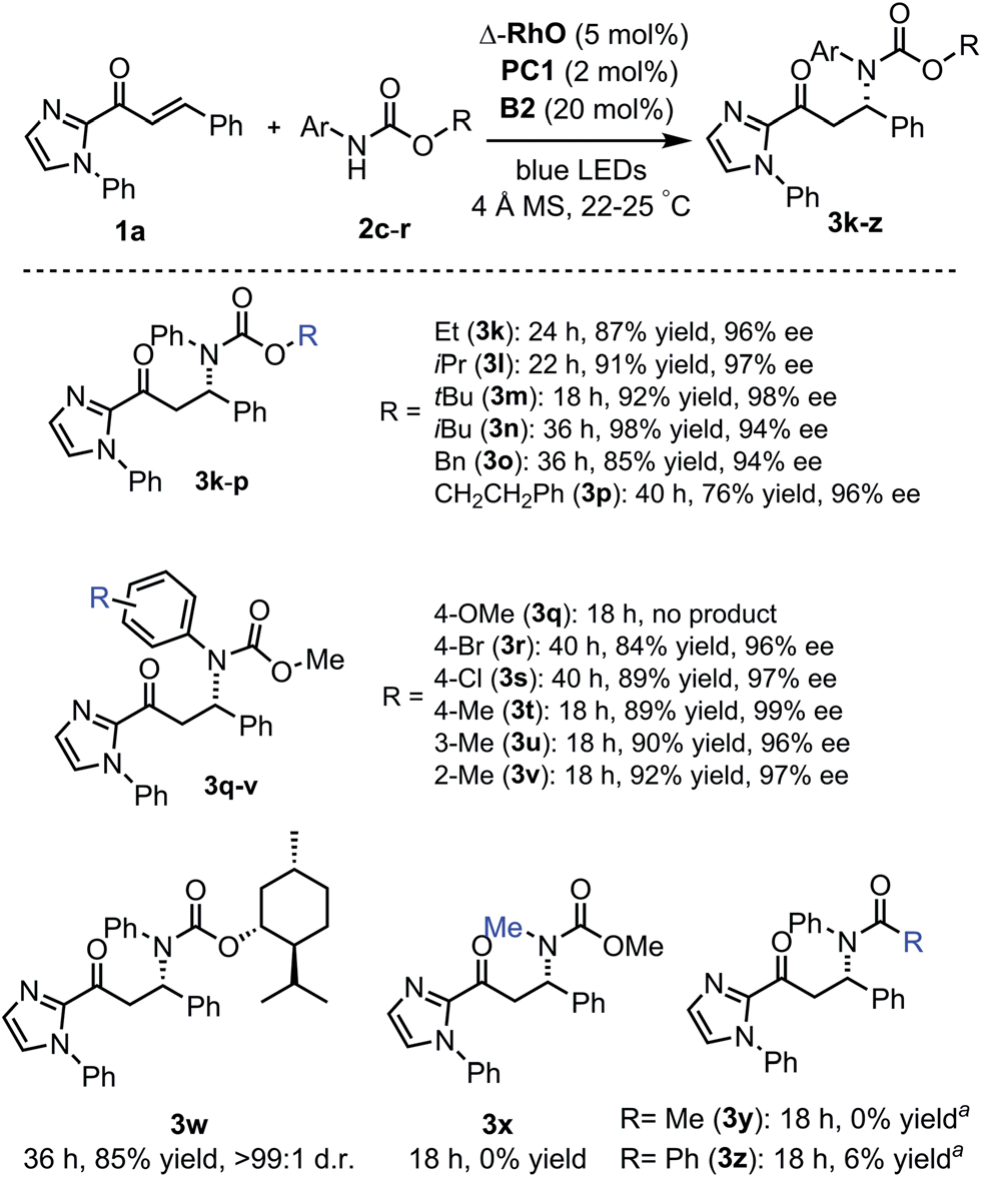
Reaction scope with respect to carbamates. ^a^No desired product formed but instead a cyclobutane side product.

Finally, the conversion of the typical product **3m** to a useful synthetic building block is illustrated in Fig. 6. Removal of the *N*-phenylimidazole moiety smoothly proceeded to provide the intermediate ester following an established procedure.^24^ Then, it was dissolved in CH_2_Cl_2_ and trifluoroacetic acid (10 equivalents) was added to cleave the Boc-protection group and subsequently form 1,2-aminoester derivative (*S*)-**3m′**, which was also used to assign the absolute configuration of the formed products in this study.^25^

**Fig. 6:**
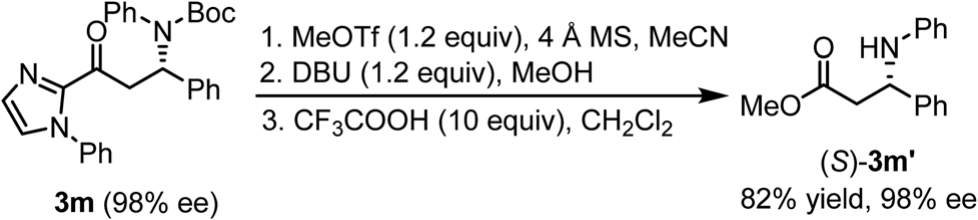
Follow-up chemistry with a cross-coupling reaction product **3m**.

In conclusion, we have introduced a novel strategy to establish a C–N bond in β-position of an α,β-unsaturated carbonyl compound in a catalytic, enantioselective fashion *via* photoinduced proton-coupled electron transfer, followed by a highly stereoselective radical–radical recombination controlled by a chiral rhodium-enolate radical intermediate. This method permits the catalytic, enantioselective synthesis of chiral amines without the necessity for a strong base or a highly nucleophilic nitrogen reagent. Although there are clear restrictions with respect to the substrate scope, within these limitations the obtained yields and enantioselectivities are excellent. Future work will need to address the currently limited scope.

## Experimental section

Representative asymmetric catalysis: to a solution of α,β-unsaturated 2-acyl imidazole **1a** (32.90 mg, 0.12 mmol) in fresh distilled anhydrous CH_2_Cl_2_ (1.0 mL) was added rhodium catalyst Δ-**RhO** (4.15 mg, 0.005 mmol), **PC1** (2.29 mg, 0.002 mmol), **B2** (9.83 mg, 0.02 mmol), 4 Å MS (10.0 mg) in a 10 mL Schlenk tube and the resulting solution was stirred at room temperature for 15 min. Then, *tert*-butyl *N*-phenylcarbamate (19.30 mg, 0.10 mmol) was added and the resulting solution was purged for 15 min with argon. The reaction was stirred under argon in a thermostatic cabinet under irradiation with blue LEDs (24 + 36 W), providing a reaction temperature of 22–25 °C. The Schlenk tube was kept at a distance of approximately 3 cm from the light source. The reaction was monitored by TLC analysis. After 18 hours full conversion was reached and the mixture was diluted with CH_2_Cl_2_ and purified by flash chromatography on silica gel (EtOAc/*n*-hexane = 1/5 to 1/3) to afford the product (*S*)-**3m** (43 mg, yield: 92%) as a white solid. The enantiomeric excess of 98% ee was established by HPLC analysis using a Daicel Chiralpak IC column (250 × 4.6 mm) under the following conditions: UV-detection at 254 nm, mobile phase *n*-hexane/isopropanol = 90 : 10, flow rate 1.0 mL min^–1^, column temperature of 25 °C. Retention times: *t*_r_(minor) = 17.44 min, *t*_r_(major) = 26.98 min. ^1^H NMR (500 MHz, CD_2_Cl_2_): *δ* (ppm) 7.47–7.37 (m, 3H), 7.30–7.14 (m, 12H), 6.89–6.76 (m, 2H), 6.10 (t, *J* = 7.5 Hz, 1H), 3.86 (dd, *J* = 15.6, 6.7 Hz, 1H), 3.55 (dd, *J* = 15.6, 8.3 Hz, 1H), 1.30 (s, 9H). ^13^C NMR (126 MHz, CD_2_Cl_2_): *δ* (ppm) 188.2, 154.5, 142.7, 140.2, 138.2, 129.7, 129.3, 128.6, 128.3, 128.0, 127.9, 127.8, 127.2, 127.0, 126.5, 125.8, 125.6, 79.8, 56.8, 41.6, 27.7. IR (film): *ν* (cm^–1^) 2963, 2920, 2859, 1692, 1404, 1260, 1093, 1020, 829, 699, 559. HRMS (ESI, *m*/*z*) calcd for C_29_H_29_N_3_NaO_3_ (M + Na)^+^: 490.2101, found: 490.2110.
