# Supplementary material for: Enantioselective catalytic β-amination through proton-coupled electron transfer followed by stereocontrolled radical–radical coupling
Source: Chem Sci. 2017 Jun 15;8(8):5757–63. doi: 10.1039/c7sc02031g (PMC5621506; doi:10.1039/c7sc02031g)
Supplement: Supplementary file 1 [file SC-008-C7SC02031G-s001.pdf]

Electronic Supplementary Material (ESI) for Chemical Science.
This journal is © The Royal Society of Chemistry 2017

Supporting Information

Enantioselective Catalytic β-Amination Through Proton-Coupled Electron Transfer Followed by Stereocontrolled Radical-Radical Coupling

Zijun Zhou, Yanjun Li, Bowen Han, Lei Gong, and Eric Meggers

Email: gongl@xmu.edu.cn, meggers@chemie.uni-marburg.de

Contents

1. General Information S02
2. Synthesis of the Substrates S03
   2.1 Synthesis of 2-acyl imidazoles S03
   2.2 Synthesis of carbamate substrates S04
3. Synthesis of the Racemic Reference Products S06
4. Δ-**RhO** Catalyzed Photoredox Reactions S07
5. Control Experiments S31
   5.1 Control experiments with a strong base S31
   5.2 Control experiment with electron-rich alkene to capture the *N*-radical S32
   5.3 Control experiment without Δ-**RhO** catalyst S33
   5.4 Control experiment with TEMPO as an additive S34
6. Follow-up Reaction of the Radical Coupling Product **3m** S35
7. Chiral Chromatography S36
8. Photoreaction in the Absence of a Photoredox Mediator S58
9. References S60
10. ^{1}H & ^{13}C NMR Spectra S61

1. General Information

All reactions were carried out under an argon atmosphere with magnetic stirring. Solvents were distilled with argon from calcium hydride (CH_3CN and CH_2Cl_2) or sodium/benzophenone (THF and toluene). Δ-**RhO**,[1] Δ-**RhS**,[2] Δ-**IrO**,[3] the substrates α,β-unsaturated 2-acyl imidazoles,[4] carbamates,[5,9] and photocatalysts[6,7] were prepared according to published procedures. All other reagents including the amide substrates **2p-r**) were purchased from commercial suppliers (TCI, Aldrich, Alfa, Adamas and J&K) and used without further purification. All photolysis experiments were performed with a combination of 24 W and 36 W blue LEDs (Company: Hongchangzhaoming, website: http://hongchang-led.taobao.com) so that the irradiation could be performed from two opposite sides of the reaction vessel. All catalytic reactions under blue LEDs irradiation were performed in a thermostatic cabinet (the temperature was set to 20 °C and the actual temperature was about 22-25 °C due to blue LEDs irradiation). Column chromatography was performed with silica gel from Huanghai Chemical Reagent (300-400 mesh). ^1H NMR and ^{13}C NMR spectra were recorded on a Bruker AM (400 MHz and 500 MHz) spectrometer at room temperature. NMR standards were used as follows: ^1H NMR spectroscopy: δ = 7.26 ppm ($CDCl_3$), 5.32 ppm (CD_2Cl_2); ^{13}C NMR spectroscopy: δ = 77.1 ppm ($CDCl_3$), CD_2Cl_2 = 53.8 ppm ($CDCl_3$); IR spectra were recorded on a Nicolet Avatar 330 FT-IR spectrophotometer. Chiral HPLC chromatograms were obtained from an Agilent 1260 Series HPLC system. High-resolution mass spectra were recorded on a Bruker En Apex Ultra 7.0T FT-MS instrument using ESI technique. Optical rotations were measured on an Anton Paar MCP 500 polarimeter at concentrations of 1.0 g/100 mL. Enantiomeric excess and diastereomeric ratios of the products were determined by HPLC on chiral phase.

2. Synthesis of the Substrates

2.1 Synthesis of 2-acyl imidazoles

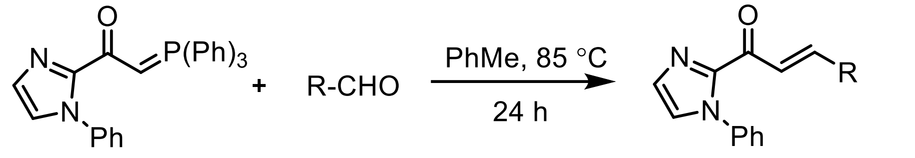

S1a

S2a (R = Ph)
S2b (R = 4-CF_3Ph)
S2c (R = 4-ClPh)
S2d (R = 4-BrPh)
S2e(R = 4-FPh)
S2f (R = 4-MePh)
S2g (R = 3-MePh)
S2h (R = 2-MePh)
S2i (R = 4-MeOPh)
S2j (R = Me)

1a (R = Ph)
1b (R = 4-CF_3Ph)
1c (R = 4-ClPh)
1d (R = 4-BrPh)
1e (R = 4-FPh)
1f (R = 4-MePh)
1g (R = 3-MePh)
1h (R = 2-MePh)
1i (R = 4-MeOPh)
1j (R = Me)

General procedure: All the α,β-unsaturated 2-acyl imidazoles in this work were synthesized according to a published procedure.[4] Accordingly, to a solution of compound **S1a** (2.0 mmol, 1 equiv) in toluene were added aldehydes **S2a-j** (4.0 mmol, 2 equiv) under an argon atmosphere. The resulting solution was heated at 85 °C for 24 h. After cooling the solution was subjected to flash chromatography on silica gel (EtOAc/*n*-hexane = 1:10-1:5) to afford α,β-unsaturated 2-acyl imidazoles **1a-j**. Then, to a solution of purified alkene in CH_2Cl_2 (0.2 M) at room temperature was added DMAP (0.1 equiv). The reaction was sealed and stored at -20 °C (fridge) for 24 h. After isomerization, the solution was passed through a short silica column. The isomerization proceeded well to afford **1j** with an improved *E/Z* value (*E/Z* > 50/1, as judged by ^{1}H NMR).

2.2 Synthesis of carbamate substrates

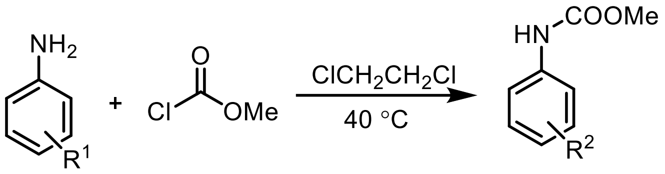

S3a (R^1 = H) | **2a** (R^2 = H)
S3i (R^1 = 4-MeOPh) | **2i** (R^2 = 4-MeOPh)
S3j (R^1 = 4-BrPh) | **2j** (R^2 = 4-BrPh)
S3k (R^1 = 4-ClPh) | **2k** (R^2 = 4-ClPh)
S3l (R^1 = 4-MePh) | **2l** (R^2 = 4-MePh)
S3m (R^1 = 3-MePh) | **2m** (R^2 = 3-MePh)
S3n (R^1 = 2-MePh) | **2n** (R^2 = 2-MePh)

General procedure: Carbamates **2a**, **2i-n** were synthesized according to a literature procedure.[5] Accordingly, sodium carbonate (20 mmol, 2.0 equiv) was added to 25 mL water and stirred until completely dissolved. Then, 25 mL of 1,2-dichloroethane was added to form a biphasic mixture. To this solution was added aniline precursor (10 mmol, 1.0 equiv), followed by methyl chloroformate (15 mmol, 1.5 equiv). This mixture was stirred for 3 h at 40 °C. The aqueous layer was then separated, and the organic layer was washed with 1 *N* HCl (2 x 50 mL) and brine (2 x 50 mL). The combined organic solution was then dried over $MgSO_4$ and filtered to yield the desired methyl aryl carbamates.

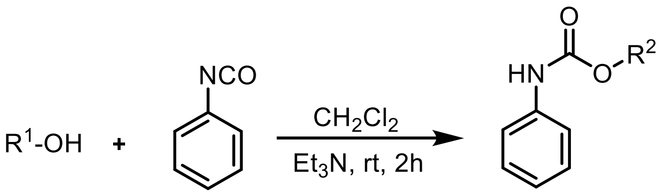

S3b (R^1 = 2-methylpent-2-eneyl) | **2b** (R^2 = 2-methylpent-2-eneyl)
S3c (R^1 = Et) | **2c** (R^2 = Et)
S3d (R^1 = *i*Pr) | **2d** (R^2 = *i*Pr)
S3e (R^1 = *t*Bu) | **2e** (R^2 = *t*Bu)
S3f (R^1 = *i*Bu) | **2f** (R^2 = *i*Bu)
S3g (R^1 = Cbz) | **2g** (R^2 = Cbz)
S3h (R^1 = CH_2CH_2Ph) | **2h** (R^2 = CH_2CH_2Ph)
S3o *L*-Menthol | **2o** *L*-Menthol

General procedure: Carbamates **2b-h**, **2o** were synthesized according to a literature procedure.[9] Accordingly, to a solution of phenyl isocyanate (596 mg, 5 mmol, 1.0 equiv) and Et_3N (5.5 mmol, 1.1 eqiuv) in CH_2Cl_2 (25 mL) was added alcohols (5 mmol, 1.0 equiv). After stirring at room

temperature for 2 h, brine was added to the solution. The aqueous layer was then separated, and the organic layer was washed with 1 *N* HCl (2 x 50 mL) and brine (2 x 50 mL). The combined solution was then dried over $MgSO_4$ and filtered to yield the desired carbamates.

3. Synthesis of the Racemic Reference Products

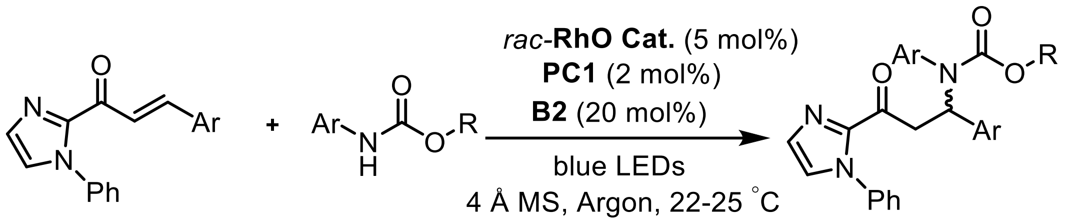

Scheme S1. Synthesis of racemic reference compounds.

General Procedure: To a solution of α,β-unsaturated 2-acyl imidazoles **1a-g**, **1i** (0.12 mmol) in fresh distilled CH_2Cl_2 (1.0 mL, 0.1 M) was added rhodium (III) complex *rac*-**RhO** (4.15 mg, 0.005 mmol, 5 mol%), **PC1** (2.29 mg, 2 mol%), **B2** (9.83 mg, 20 mol%), 4 Å MS (10.0 mg, 100 mg/mmol) in a 10 mL Schlenk tube and stirred at room temperature for 15 min. Then, carbamate **2a-h**, **2j-r** (0.10 mmol) was added and the solution was purged for 15 min with argon. The reaction was stirred in a thermostatic cabinet (the actual temperature was about 22-25 °C) under argon atmosphere while irradiating with two blue LEDs (24 W + 36 W) from opposite sites (approximately 3 cm distance from the light source). The reaction was monitored by TLC analysis. Afterwards, the mixture was diluted with CH_2Cl_2 and purified by flash chromatography on silica gel (EtOAc/*n*-hexane = 1:5-1:3) to afford *rac*-**3a-g**, **3i**, **3k-p**, **3r-w** as HPLC references for the determination of enantiomeric excess and diastereomeric ratio.

4. Δ-RhO Catalyzed Photoredox Reactions

General Procedure: To a solution of α,β-unsaturated 2-acyl imidazoles **1a-j** (0.12 mmol, 1.2 equiv) in fresh distilled CH_2Cl_2 (1.0 mL, 0.1 M) was added rhodium (III) complex Δ-**RhO** (4.15 mg, 0.005 mmol, 5 mol%), **PC1** (2.29 mg, 2 mol%), **B2** (9.83 mg, 20 mol%), and 4 Å MS (10.0 mg, 100 mg/mmol) in a 10 mL Schlenk tube and the resulting solution was stirred at room temperature for 15 min. Then, carbamate **2a-r** (0.10 mmol, 1.0 equiv) was added and the solution was purged for 15 min with argon and the reaction executed under argon atmosphere. The reaction was stirred in a thermostatic cabinet (the actual temperature was about 22-25 °C) and photolyzed with two blue LEDs (24 W+36 W) from opposite sites at distances to the Schlenk tube of approximately 3 cm. The reaction was monitored by TLC analysis. Afterwards, the mixture was diluted with CH_2Cl_2 and purified by flash chromatography on silica gel (EtOAc/*n*-hexane = 1:5-1:3) to afford **3a-g**, **3i**, **3k-p**, and **3r-w**. The compounds **3h**, **3j**, **3q**, and **3x-z** were not formed.

Compound 3a

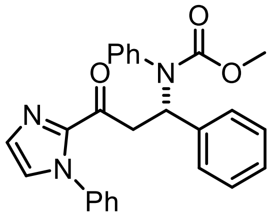

To a solution of α,β-unsaturated 2-acyl imidazole **1a** (32.90 mg, 0.12 mmol) in fresh distilled CH_2Cl_2 (1.0 mL, 0.1 M) was added rhodium (III) complex Δ-**RhO** (4.15 mg, 0.005 mmol, 5 mol%), **PC1** (2.29 mg, 2 mol%), **B2** (9.83 mg, 20 mol%), 4 Å MS (10.0 mg, 100 mg/mmol) in a 10 mL Schlenk tube and the resulting solution was stirred at room temperature for 15 min. Then, carbamate **2a** (15.10 mg, 0.10 mmol) was added and the solution was bubbled for 15 min with argon. The reaction was stirred in a thermostatic cabinet (the actual temperature was about 22-25 °C) under blue LEDs (24 W+36 W) irradiation (the Schlenk tube was kept at a distance of approximately 3 cm to the light source) for 18 h. Afterwards, the mixture was diluted with CH_2Cl_2 and purified by flash chromatography (EtOAc/*n*-hexane = 1:5-1:3) to afford **3a** (40.42 mg, yield: 95%) as a white solid. Enantiomeric excess was established by HPLC analysis using a Chiralpak IA

column, ee = 98% (HPLC: IA, 254 nm, *n*-hexane/isopropanol = 90:10, flow rate 1.0 mL/min, 25 °C, t_r(minor) = 16.45 min, t_r(major) = 17.58 min). $[\alpha]_D^{20}$ = +0.55° (*c* = 1.0, $CHCl_3$).

^{1}H NMR (500 MHz, $CDCl_3$): δ (ppm) 7.45-7.38 (m, 3H), 7.26-7.17 (m, 11H), 7.17-7.14 (m, 1H), 6.86-6.76 (m, 2H), 6.23 (t, *J* = 7.5 Hz, 1H), 3.92 (dd, *J* = 15.7, 6.5 Hz, 1H), 3.70-3.51 (m, 4H).

^{13}C NMR (126 MHz, $CDCl_3$): δ (ppm) 187.1, 155.3, 141.8, 138.4, 137.2, 129.1, 128.6, 127.9, 127.7, 127.5, 127.3, 127.2, 126.7, 126.5, 126.1, 124.8, 56.3, 51.9, 40.5, 28.7.

IR (film): ν (cm^{-1}) 2962, 2919, 2849, 1693, 1408, 1260, 1093, 1019, 828, 801, 698, 560.

HRMS (ESI, *m*/*z*) calcd for $C_{26}H_{24}N_3O_3$ (M+H)$^+$: 426.1812, found: 426.1813.

Compound 3b

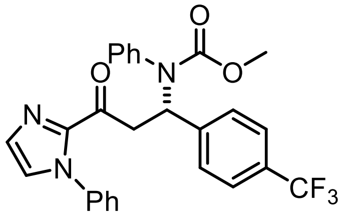

To a solution of α,β-unsaturated 2-acyl imidazole **1b** (41.10 mg, 0.12 mmol) in fresh distilled CH_2Cl_2 (1.0 mL, 0.1 M) was added rhodium (III) complex Δ-**RhO** (4.15 mg, 0.005 mmol, 5 mol%), **PC1** (2.29 mg, 2 mol%), **B2** (9.83 mg, 20 mol%), 4 Å MS (10.0 mg, 100 mg/mmol) in a 10 mL Schlenk tube and the resulting solution was stirred at room temperature for 15 min. Then, carbamate **2a** (15.10 mg, 0.10 mmol) was added and the solution was bubbled for 15 min with argon. The reaction was stirred in a thermostatic cabinet (the actual temperature was about 22-25 °C) under blue LEDs (24 W+36 W) irradiation (the Schlenk tube was kept at a distance of approximately 3 cm to the light source) for 16 h. Afterwards, the mixture was diluted with CH_2Cl_2 and purified by flash chromatography (EtOAc/*n*-hexane = 1:5-1:3) to afford **3b** (47.73 mg, yield: 97%) as a white solid. Enantiomeric excess was established by HPLC analysis using a Chiralpak IA column, ee = 98% (HPLC: IA, 254 nm, *n*-hexane/isopropanol = 90:10, flow rate 1.0 mL/min, 25 °C, t_r(minor) = 17.99 min, t_r(major) = 20.03 min). $[\alpha]_D^{20}$ = −19.86° (*c* = 1.0, $CHCl_3$).

^{1}H NMR (500 MHz, CD_2Cl_2): *δ* (ppm) 7.53 (d, *J* = 8.2 Hz, 2H), 7.48-7.42 (m, 3H), 7.38 (d, *J* = 8.1 Hz, 2H), 7.30-7.26 (m, 4H), 7.26-7.21 (m, 2H), 7.20 (s, 1H), 6.90-6.80 (m, 2H), 6.24 (t, *J* = 7.4 Hz, 1H), 3.97 (dd, *J* = 16.2, 7.0 Hz, 1H), 3.73-3.58 (m, 4H);

^{13}C NMR (126 MHz, $CDCl_3$): *δ* (ppm) 187.7, 156.4, 143.7, 142.7, 138.5, 138.2, 130.1, 129.90, 129.86, 129.1, 128.9, 128.7, 127.9, 127.5, 126.0, 125.9, 125.3 (q, *J* = 3.7 Hz) ,123.1, 57.0, 53.5, 53.1, 41.5.

IR (film): *ν* (cm^{-1}) 2962, 2922, 2851, 1702, 1596, 1493, 1444, 1406, 1325, 1260, 1095, 1019, 826, 801, 559.

HRMS (ESI, *m/z*) calcd for $C_{27}H_{23}F_3N_3O_3$ $(M+H)^+$: 494.1686, found: 494.1689.

Compound 3c

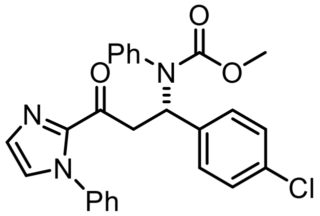

To a solution of α,β-unsaturated 2-acyl imidazole **1c** (36.96 mg, 0.12 mmol) in fresh distilled CH_2Cl_2 (1.0 mL, 0.1 M) was added rhodium (III) complex Δ-**RhO** (4.15 mg, 0.005 mmol, 5 mol%), **PC1** (2.29 mg, 2 mol%), **B2** (9.83 mg, 20 mol%), 4 Å MS (10.0 mg, 100 mg/mmol) in a 10 mL Schlenk tube and the resulting solution was stirred at room temperature for 15 min. Then, carbamate **2a** (15.10 mg, 0.10 mmol) was added and the solution was bubbled for 15 min with argon. The reaction was stirred in a thermostatic cabinet (the actual temperature was about 22-25 °C) under blue LEDs (24 W+36 W) irradiation (the Schlenk tube was kept at a distance of approximately 3 cm to the light source) for 16 h. Afterwards, the mixture was diluted with CH_2Cl_2 and purified by flash chromatography (EtOAc/*n*-hexane = 1:5-1:3) to afford **3c** (43.55 mg, yield: 95%) as a white solid. Enantiomeric excess was established by HPLC analysis using a Chiralpak IA column, ee = 98% (HPLC: IA, 254 nm, *n*-hexane/isopropanol = 90:10, flow rate 1.0 mL/min, 25 °C,

t_r(minor) = 19.35 min, t_r(major) = 21.21 min). $[\alpha]_D^{20}$ = +4.17° (*c* = 1.0, $CHCl_3$).

^{1}H NMR (500 MHz, CD_2Cl_2): *δ* (ppm) 7.39-7.33 (m, 3H), 7.19-7.10 (m, 9H), 7.08 (d, *J* = 8.5 Hz, 2H), 6.73 (dd, *J* = 7.5, 1.8 Hz, 2H), 6.01 (t, *J* = 7.6 Hz, 1H), 3.76 (dd, *J* = 16.2, 7.0 Hz, 1H), 3.55-3.46 (m, 4H).

^{13}C NMR (126 MHz, CD_2Cl_2): *δ* (ppm) 187.9, 156.2, 142.9, 138.7, 138.6, 138.5, 133.5, 130.1, 129.9, 129.8, 129.0, 128.81, 128.78, 128.4, 127.7, 127.6, 126.0, 60.4, 56.7, 41.4.

IR (film): *ν* (cm^{-1}) 2958, 2923, 2853, 1698, 1456, 1260, 1093, 1018, 864, 800, 699.

HRMS (ESI, *m*/*z*) calcd for $C_{26}H_{23}ClN_3O_3$ $(M+H)^+$: 460.1422, found: 460.1424.

Compound 3d

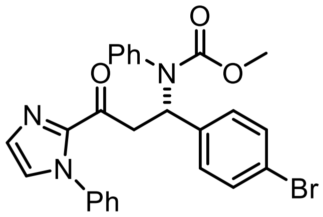

To a solution of α,β-unsaturated 2-acyl imidazole **1d** (42.24 mg, 0.12 mmol) in fresh distilled CH_2Cl_2 (1.0 mL, 0.1 M) was added rhodium (III) complex Δ-**RhO** (4.15 mg, 0.005 mmol, 5 mol%), **PC1** (2.29 mg, 2 mol%), **B2** (9.83 mg, 20 mol%), 4 Å MS (10.0 mg, 100 mg/mmol) in a 10 mL Schlenk tube and the resulting solution was stirred at room temperature for 15 min. Then, carbamate **2a** (15.10 mg, 0.10 mmol) was added and the solution was bubbled for 15 min with argon. The reaction was stirred in a thermostatic cabinet (the actual temperature was about 22-25 °C) under blue LEDs (24 W+36 W) irradiation (the Schlenk tube was kept at a distance of approximately 3 cm to the light source) for 17 h. Afterwards, the mixture was diluted with CH_2Cl_2 and purified by flash chromatography (EtOAc/*n*-hexane = 1:5-1:3) to afford **3d** (41.33 mg, yield: 82%) as a white solid. Enantiomeric excess was established by HPLC analysis using a Chiralpak IA column, ee = 98% (HPLC: IA, 254 nm, *n*-hexane/isopropanol = 90:10, flow rate 1.0 mL/min, 25 °C,

t_r(minor) = 20.89 min, t_r(major) = 22.64 min). $[\alpha]_D^{20}$ = +15.88° (c = 1.0, $CHCl_3$).

^{1}H NMR (500 MHz, CD_2Cl_2): δ (ppm) 7.40-7.33 (m, 3H), 7.30 (d, J = 8.4 Hz, 2H), 7.17 (m, 3H), 7.16-7.13 (m, 3H), 7.12 (s, 1H), 7.03 (d, J = 8.4 Hz, 2H), 6.73 (dd, J = 7.4, 1.9 Hz, 2H), 5.99 (t, J = 7.6 Hz, 1H), 3.75 (dd, J = 16.2, 7.0 Hz, 1H), 3.51 (m, 4H).

^{13}C NMR (126 MHz, CD_2Cl_2): δ (ppm) 187.9, 156.2, 142.9, 139.1, 138.7, 138.5, 131.4, 130.2, 130.1, 129.8, 129.0, 128.81, 128.78, 127.8, 127.6, 126.0, 121.7, 60.4, 56.8, 41.4.

IR (film): ν (cm^{-1}) 2961, 2917, 2849, 1692, 1594, 1490, 1403, 1259, 1092, 1017, 827, 799, 692, 559.

HRMS (ESI, m/z) calcd for $C_{26}H_{23}BrN_3O_3$ $(M+H)^+$: 504.0917, found: 504.0920.

Compound 3e

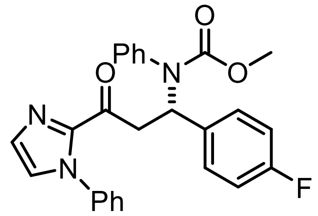

To a solution of α,β-unsaturated 2-acyl imidazole **1e** (35.10 mg, 0.12 mmol) in fresh distilled CH_2Cl_2 (1.0 mL, 0.1 M) was added rhodium (III) complex Δ-**RhO** (4.15 mg, 0.005 mmol, 5 mol%), **PC1** (2.29 mg, 2 mol%), **B2** (9.83 mg, 20 mol%), 4 Å MS (10.0 mg, 100 mg/mmol) in a 10 mL Schlenk tube and the resulting solution was stirred at room temperature for 15 min. Then, carbamate **2a** (15.10 mg, 0.10 mmol) was added and the solution was bubbled for 15 min with argon. The reaction was stirred in a thermostatic cabinet (the actual temperature was about 22-25 °C) under blue LEDs (24 W+36 W) irradiation (the Schlenk tube was kept at a distance of approximately 3 cm to the light source) for 16 h. Afterwards, the mixture was diluted with CH_2Cl_2 and purified by flash chromatography (EtOAc/n-hexane = 1:5-1:3) to afford **3e** (40.80 mg, yield: 92%) as a white solid. Enantiomeric excess was established by HPLC analysis using a Chiralpak IA column, ee = 99% (HPLC: IA, 254 nm, n-hexane/isopropanol = 90:10, flow rate 1.0 mL/min, 25 °C, t_r(minor) = 17.29 min, t_r(major) = 19.27 min). $[\alpha]_D^{20}$ = −20.81° (c = 1.0, $CHCl_3$).

^{1}H NMR (500 MHz, CD_2Cl_2): δ (ppm) 7.42-7.29 (m, 3H), 7.22-7.05 (m, 9H), 6.86 (t, J = 8.7 Hz, 2H), 6.77-6.65 (m, 2H), 6.04 (t, J = 8.7 Hz, 1H), 3.76 (dd, J = 16.0, 6.9 Hz, 1H), 3.49 (m, J = 8.4 Hz, 4H).

^{13}C NMR (126 MHz, CD_2Cl_2): δ (ppm) 187.2, 162.5, 160.5, 155.3, 142.0, 137.7 (d, J = 14.5 Hz), 134.9, 129.3, 129.2, 128.8, 128.1, 127.9, 126.8, 126.7, 125.1, 114.2, 114.1, 55.8, 52.0, 40.7.

IR (film): ν (cm^{-1}) 2961, 2918, 2849, 1697, 1596, 1509, 1493, 1404, 1306, 1260, 1094, 1018, 799, 765, 698.

HRMS (ESI, *m/z*) calcd for $C_{26}H_{23}FN_3O_3$ $(M+H)^+$: 444.1718, found: 444.1719.

Compound 3f

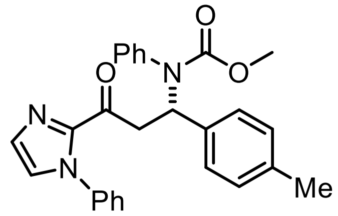

To a solution of α,β-unsaturated 2-acyl imidazole **1f** (34.60 mg, 0.12 mmol) in fresh distilled CH_2Cl_2 (1.0 mL, 0.1 M) was added rhodium (III) complex Δ-**RhO** (4.15 mg, 0.005 mmol, 5 mol%), **PC1** (2.29 mg, 2 mol%), **B2** (9.83 mg, 20 mol%), 4 Å MS (10.0 mg, 100 mg/mmol) in a 10 mL Schlenk tube and the resulting solution was stirred at room temperature for 15 min. Then, carbamate **2a** (15.10 mg, 0.10 mmol) was added and the solution was bubbled for 15 min with argon. The reaction was stirred in a thermostatic cabinet (the actual temperature was about 22-25 °C) under blue LEDs (24 W+36 W) irradiation (the Schlenk tube was kept at a distance of approximately 3 cm to the light source) for 18 h. Afterwards, the mixture was diluted with CH_2Cl_2 and purified by flash chromatography (EtOAc/*n*-hexane = 1:5-1:3) to afford **3f** (37.69 mg, yield: 86%) as a white solid. Enantiomeric excess was established by HPLC analysis using a Chiralpak IA column, ee = 97% (HPLC: IA, 254 nm, *n*-hexane/isopropanol = 99:1, flow rate 1.0 mL/min, 25 °C, t_r(minor) = 64.46 min, t_r(major) = 70.43 min). $[\alpha]_D^{20} = -5.12°$ (c = 1.0, $CHCl_3$).

^{1}H NMR (500 MHz, CD_2Cl_2): δ (ppm) 7.47-7.37 (m, 3H), 7.26-7.15 (m, 7H), 7.10-7.02 (m, 4H),

6.81 (dd, J = 7.7, 1.7 Hz, 2H), 6.08 (t, J = 7.6 Hz, 1H), 3.83 (dd, J = 16.0, 6.8 Hz, 1H), 3.65-3.45 (m, 4H), 2.30 (s, 3H).

^{13}C NMR (126 MHz, CD_2Cl_2): δ (ppm) 188.4, 156.2, 143.0, 139.0, 138.6, 137.6, 136.8, 130.2, 129.7, 129.0, 128.9, 128.7, 128.6, 128.2, 127.5, 127.4, 126.0, 57.1, 52.8, 41.6, 20.9.

IR (film): ν (cm^{-1}) 2962, 2919, 2850, 1595, 1492, 1441, 1260, 1093, 1018, 964, 799, 693, 559.

HRMS (ESI, m/z) calcd for $C_{27}H_{25}N_3NaO_3$ $(M+Na)^+$: 462.1788, found: 462.1789.

Compound 3g

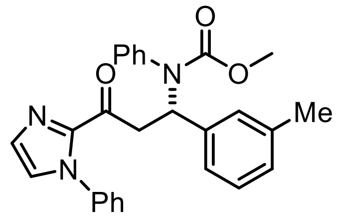

To a solution of α,β-unsaturated 2-acyl imidazole **1g** (34.60 mg, 0.12 mmol) in fresh distilled CH_2Cl_2 (1.0 mL, 0.1 M) was added rhodium (III) complex Δ-**RhO** (4.15 mg, 0.005 mmol, 5 mol%), **PC1** (2.29 mg, 2 mol%), **B2** (9.83 mg, 20 mol%), 4 Å MS (10.0 mg, 100 mg/mmol) in a 10 mL Schlenk tube and the resulting solution was stirred at room temperature for 15 min. Then, carbamate **2a** (15.10 mg, 0.10 mmol) was added and the solution was bubbled for 15 min with argon. The reaction was stirred in a thermostatic cabinet (the actual temperature was about 22-25 °C) under blue LEDs (24 W+36 W) irradiation (the Schlenk tube was kept at a distance of approximately 3 cm to the light source) for 18 h. Afterwards, the mixture was diluted with CH_2Cl_2 and purified by flash chromatography (EtOAc/n-hexane = 1:5-1:3) to afford **3g** (38.96 mg, yield: 89%) as a white solid. Enantiomeric excess was established by HPLC analysis using a Chiralpak IA column, ee = 94% (HPLC: IA, 254 nm, n-hexane/isopropanol = 90:10, flow rate 1.0 mL/min, 25 °C, t_r(minor) = 12.64 min, t_r(major) = 13.81 min). $[\alpha]_D^{20}$ = −4.96° (c = 1.0, $CHCl_3$).

^1H NMR (500 MHz, CD_2Cl_2): δ (ppm) 7.48-7.39 (m, 3H), 7.26-7.16 (m, 7H), 7.11 (t, J = 7.5 Hz, 1H), 7.08-7.02 (m, 2H), 6.95 (d, J = 7.5 Hz, 1H), 6.80 (d, J = 6.2 Hz, 2H), 6.09 (t, J = 7.5 Hz, 1H), 3.83 (dd, J = 16.0, 6.6 Hz, 1H), 3.64-3.50 (m, 4H), 2.27 (s, 3H).

^{13}C NMR (126 MHz, CD_2Cl_2): δ (ppm) 188.4, 156.3, 143.0, 139.7, 138.9, 138.6, 138.1, 130.2, 129.7, 129.2, 129.0, 128.7, 128.6, 128.5, 128.1, 127.5, 127.4, 126.0, 125.2, 57.3, 52.8, 41.5, 21.2.

IR (film): ν (cm^{-1}) 2961, 2919, 2849, 1698, 1443, 1407, 1260, 1093, 1019, 829, 699, 559.

HRMS (ESI, *m/z*) calcd for $C_{27}H_{25}N_3NaO_3$ $(M+Na)^+$: 462.1788, found: 462.1789.

Compound 3h

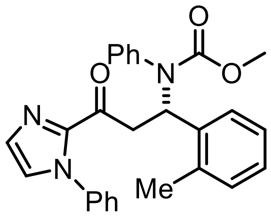

To a solution of α,β-unsaturated 2-acyl imidazole **1h** (34.60 mg, 0.12 mmol) in fresh distilled CH_2Cl_2 (1.0 mL, 0.1 M) was added rhodium (III) complex Δ-**RhO** (4.15 mg, 0.005 mmol, 5 mol%), **PC1** (2.29 mg, 2 mol%), **B2** (9.83 mg, 20 mol%), 4 Å MS (10.0 mg, 100 mg/mmol) in a 10 mL Schlenk tube and the resulting solution was stirred at room temperature for 15 min. Then, carbamate **2a** (15.10 mg, 0.10 mmol) was added and the solution was bubbled for 15 min with argon. The reaction was stirred in a thermostatic cabinet (the actual temperature was about 22-25 °C) under blue LEDs (24 W+36 W) irradiation (the Schlenk tube was kept at a distance of approximately 3 cm to the light source) for 18 h. Afterwards, the mixture was monitored by crude ^1H NMR. Compound **3h** was only formed in trace amounts. The main product was a cyclobutane side product (<10 % yield).

Compound 3i

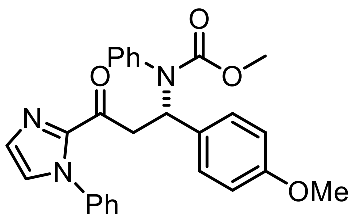

To a solution of α,β-unsaturated 2-acyl imidazole **1i** (36.50 mg, 0.12 mmol) in fresh distilled CH_2Cl_2 (1.0 mL, 0.1 M) was added rhodium (III) complex Δ-**RhO** (4.15 mg, 0.005 mmol, 5 mol%),

PC1 (2.29 mg, 2 mol%), **B2** (9.83 mg, 20 mol%), 4 Å MS (10.0 mg, 100 mg/mmol) in a 10 mL Schlenk tube and the resulting solution was stirred at room temperature for 15 min. Then, carbamate **2a** (15.10 mg, 0.10 mmol) was added and the solution was bubbled for 15 min with argon. The reaction was stirred in a thermostatic cabinet (the actual temperature was about 22-25 °C) under blue LEDs (24 W+36 W) irradiation (the Schlenk tube was kept at a distance of approximately 3 cm to the light source) for 72 h. Afterwards, the mixture was diluted with CH_2Cl_2 and purified by flash chromatography (EtOAc/*n*-hexane = 1:5-1:3) to afford **3i** (39.55 mg, yield: 87%) as a white solid. Enantiomeric excess was established by HPLC analysis using a Chiralpak IA column, ee = 97% (HPLC: IA, 254 nm, *n*-hexane/isopropanol = 90:10, flow rate 1.0 mL/min, 25 °C, t_r(minor) = 24.86 min, t_r(major) = 26.85 min). $[\alpha]_D^{20}$ = +1.28° (*c* = 1.0, $CHCl_3$).

^{1}H NMR (500 MHz, $CDCl_3$): δ (ppm) 7.45-7.38 (m, 3H), 7.26-7.17 (m, 6H), 7.15 (d, *J* = 0.9 Hz, 1H), 7.11 (t, *J* = 5.7 Hz, 2H), 6.81 (dd, *J* = 7.5, 1.8 Hz, 2H), 6.78-6.72 (m, 2H), 6.19 (t, *J* = 7.5 Hz, 1H), 3.88 (dd, *J* = 15.6, 6.7 Hz, 1H), 3.77 (s, 3H), 3.61 (s, 3H), 3.54 (dd, *J* = 15.6, 8.5 Hz, 1H).

^{13}C NMR (126 MHz, $CDCl_3$): δ (ppm) 188.3, 159.1, 156.4, 143.0, 138.4, 131.7, 130.3, 129.7, 129.7, 129.0, 128.7, 128.6, 127.6, 127.2, 125.9, 113.8, 113.5, 56.9, 55.3, 53.0, 41.8.

IR (film): ν (cm^{-1}) 2962, 2920, 2850, 1693, 1441, 1404, 1260, 1093, 1019, 826, 800, 699, 560.

HRMS (ESI, *m*/*z*) calcd for $C_{27}H_{25}N_3NaO_4$ $(M+Na)^+$: 478.1737, found: 478.1738.

Compound 3j

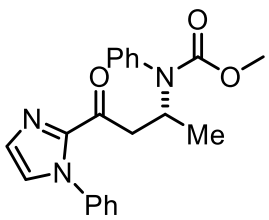

To a solution of α,β-unsaturated 2-acyl imidazole **1j** (25.44 mg, 0.12 mmol) in fresh distilled CH_2Cl_2 (1.0 mL, 0.1 M) was added rhodium (III) complex Δ-**RhO** (4.15 mg, 0.005 mmol, 5 mol%), **PC1** (2.29 mg, 2 mol%), **B2** (9.83 mg, 20 mol%), 4 Å MS (10.0 mg, 100 mg/mmol) in a 10 mL

Schlenk tube and the resulting solution was stirred at room temperature for 15 min. Then, carbamate **2a** (15.10 mg, 0.10 mmol) was added and the solution was bubbled for 15 min with argon. The reaction was stirred in a thermostatic cabinet (the actual temperature was about 22-25 °C) under blue LEDs (24 W+36 W) irradiation (the Schlenk tube was kept at a distance of approximately 3 cm to the light source). After 18 h, the mixture was monitored by crude ^{1}H NMR. The compound **3j** was only formed in trace amounts.

Compound 3k

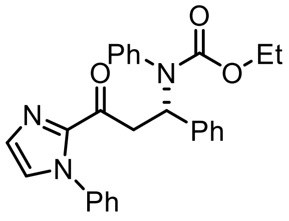

To a solution of α,β-unsaturated 2-acyl imidazole **1a** (32.90 mg, 0.12 mmol) in fresh distilled CH_2Cl_2 (1.0 mL, 0.1 M) was added rhodium (III) complex Δ-**RhO** (4.15 mg, 0.005 mmol, 5 mol%), **PC1** (2.29 mg, 2 mol%), **B2** (9.83 mg, 20 mol%), 4 Å MS (10.0 mg, 100 mg/mmol) in a 10 mL Schlenk tube and the resulting solution was stirred at room temperature for 15 min. Then, carbamate **2c** (16.50 mg, 0.10 mmol) was added and the solution was bubbled for 15 min with argon. The reaction was stirred in a thermostatic cabinet (the actual temperature was about 22-25 °C) under blue LEDs (24 W+36 W) irradiation (the Schlenk tube was kept at a distance of approximately 3 cm to the light source) for 24 h. Afterwards, the mixture was diluted with CH_2Cl_2 and purified by flash chromatography (EtOAc/*n*-hexane = 1:5-1:3) to afford **3k** (38.16 mg, yield: 87%) as a white solid. Enantiomeric excess was established by HPLC analysis using a Chiralpak IC column, ee = 96% (HPLC: IC, 254 nm, *n*-hexane/isopropanol = 85:15, flow rate 1.0 mL/min, 25 °C, t_r(minor) = 29.26 min, t_r(major) = 55.39 min). $[\alpha]_D^{20}$ = −5.89 (*c* = 1.0, $CHCl_3$).

^{1}H NMR (500 MHz, $CDCl_3$): δ (ppm) 7.45-7.38 (m, 3H), 7.25 (d, J = 0.9 Hz, 1H), 7.24-7.18 (m, 10H), 7.16 (d, J = 0.9 Hz, 1H), 6.82 (dd, J = 4.9, 2.8 Hz, 2H), 6.23 (t, J = 7.5 Hz, 1H), 4.17-4.01 (m, 2H), 3.92 (dd, J = 15.6, 6.5 Hz, 1H), 3.58 (dd, J = 15.6, 8.6 Hz, 1H), 1.10 (t, J = 6.8 Hz, 3H).

^{13}C NMR (126 MHz, $CDCl_3$): δ (ppm) 187.2, 154.9, 141.9, 138.7, 137.7, 137.3, 129.1, 128.6, 127.9, 127.6, 127.4, 127.3, 127.1, 126.6, 126.3, 126.1, 124.8, 60.6, 56.3, 40.6, 13.5.

IR (film): ν (cm^{-1}) 2962, 2920, 2859, 1687, 1597, 1493, 1445, 1404, 1305, 1260, 1091, 1019, 799, 695.

HRMS (ESI, *m/z*) calcd for $C_{27}H_{25}N_3NaO_3$ $(M+Na)^+$: 462.1788, found: 462.1788

Compound 3l

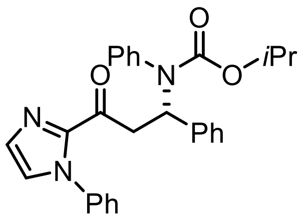

To a solution of α,β-unsaturated 2-acyl imidazole **1a** (32.90 mg, 0.12 mmol) in fresh distilled CH_2Cl_2 (1.0 mL, 0.1 M) was added rhodium (III) complex Δ-**RhO** (4.15 mg, 0.005 mmol, 5 mol%), **PC1** (2.29 mg, 2 mol%), **B2** (9.83 mg, 20 mol%), 4 Å MS (10.0 mg, 100 mg/mmol) in a 10 mL Schlenk tube and the resulting solution was stirred at room temperature for 15 min. Then, carbamate **2d** (17.90 mg, 0.10 mmol) was added and the solution was bubbled for 15 min with argon. The reaction was stirred in a thermostatic cabinet (the actual temperature was about 22-25 °C) under blue LEDs (24 W+36 W) irradiation (the Schlenk tube was kept at a distance of approximately 3 cm to the light source) for 22 h. Afterwards, the mixture was diluted with CH_2Cl_2 and purified by flash chromatography (EtOAc/*n*-hexane = 1:5-1:3) to afford **3l** (41.24 mg, yield: 91%) as a white solid. Enantiomeric excess was established by HPLC analysis using a Chiralpak IC column, ee = 97% (HPLC: IC, 254 nm, *n*-hexane/isopropanol = 80:20, flow rate 1.0 mL/min, 25 °C, t_r(minor) = 15.02 min, t_r(major) = 27.42 min). $[\alpha]_D^{20}$ = −9.74° (*c* = 1.0, $CHCl_3$).

^1H NMR (500 MHz, CD_2Cl_2): δ (ppm) 7.46-7.38 (m, 3H), 7.31-7.13 (m, 12H), 6.81 (dd, *J* = 6.4, 3.1 Hz, 2H), 6.16-6.08 (m, 1H), 4.87 (hept, *J* = 6.2 Hz, 1H), 3.86 (dd, *J* = 15.9, 6.8 Hz, 1H), 3.57 (dd, *J* = 15.9, 8.3 Hz, 1H), 1.08 (dd, *J* = 21.7, 6.0 Hz, 6H).

^{13}C NMR (126 MHz, CD_2Cl_2): δ (ppm) 188.7, 155.7, 143.3, 140.5, 138.8, 130.3, 129.9, 129.2, 128.9, 128.7, 128.5, 128.5, 127.9, 127.7, 127.4, 126.3, 126.2, 69.4, 57.6, 42.0, 22.1.

IR (film): v (cm^{-1}) 2961, 2920, 2859, 1688, 1493, 1405, 1260, 1092, 1019, 830, 697, 560.

HRMS (ESI, *m*/*z*) calcd for $C_{28}H_{27}N_3NaO_3$ $(M+Na)^+$: 476.1945, found: 476.1946.

Compound 3m

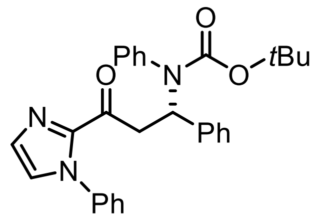

To a solution of α,β-unsaturated 2-acyl imidazole **1a** (32.90 mg, 0.12 mmol) in fresh distilled CH_2Cl_2 (1.0 mL, 0.1 M) was added rhodium (III) complex Δ-**RhO** (4.15 mg, 0.005 mmol, 5 mol%), **PC1** (2.29 mg, 2 mol%), **B2** (9.83 mg, 20 mol%), 4 Å MS (10.0 mg, 100 mg/mmol) in a 10 mL Schlenk tube and the resulting solution was stirred at room temperature for 15 min. Then, carbamate **2e** (19.30 mg, 0.10 mmol) was added and the solution was bubbled for 15 min with argon. The reaction was stirred in a thermostatic cabinet (the actual temperature was about 22-25 °C) under blue LEDs (24 W+36 W) irradiation (the Schlenk tube was kept at a distance of approximately 3 cm to the light source) for 18 h. Afterwards, the mixture was diluted with CH_2Cl_2 and purified by flash chromatography (EtOAc/*n*-hexane = 1:5-1:3) to afford **3m** (42.89 mg, yield: 92%) as a white solid. Enantiomeric excess was established by HPLC analysis using a Chiralpak IC column, ee = 98% (HPLC: IC, 254 nm, *n*-hexane/isopropanol = 90:10, flow rate 1.0 mL/min, 25 °C, t_r(minor) = 17.45 min, t_r(major) = 26.98 min). $[\alpha]_D^{20} = -12.15°$ (c = 1.0, $CHCl_3$).

^1H NMR (500 MHz, CD_2Cl_2): δ (ppm) 7.47-7.37 (m, 3H), 7.30-7.14 (m, 12H), 6.89-6.76 (m, 2H), 6.10 (t, J = 7.5 Hz, 1H), 3.86 (dd, J = 15.6, 6.7 Hz, 1H), 3.55 (dd, J = 15.6, 8.3 Hz, 1H), 1.30 (s, 9H).

^{13}C NMR (126 MHz, CD_2Cl_2): δ (ppm) 188.2, 154.5, 142.7, 140.2, 138.2, 129.7, 129.3, 128.6, 128.3, 128.0, 127.9, 127.8, 127.2, 127.0, 126.5, 125.8, 125.6, 79.8, 56.8, 41.6, 27.7.

IR (film): ν (cm^{-1}) 2963, 2920, 2859, 1692, 1404, 1260, 1093, 1020, 829, 699, 559.

HRMS (ESI, *m*/*z*) calcd for $C_{29}H_{29}N_3NaO_3$ $(M+Na)^+$: 490.2101, found: 490.2110.

Compound 3n

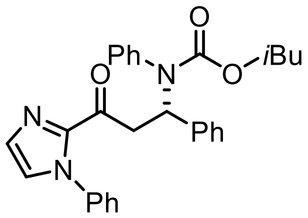

To a solution of α,β-unsaturated 2-acyl imidazole **1a** (32.90 mg, 0.12 mmol) in fresh distilled CH_2Cl_2 (1.0 mL, 0.1 M) was added rhodium (III) complex Δ-**RhO** (4.15 mg, 0.005 mmol, 5 mol%), **PC1** (2.29 mg, 2 mol%), **B2** (9.83 mg, 20 mol%), 4 Å MS (10.0 mg, 100 mg/mmol) in a 10 mL Schlenk tube and the resulting solution was stirred at room temperature for 15 min. Then, carbamate **2f** (19.30 mg, 0.10 mmol) was added and the solution was bubbled for 15 min with argon. The reaction was stirred in a thermostatic cabinet (the actual temperature was about 22-25 °C) under blue LEDs (24 W+36 W) irradiation (the Schlenk tube was kept at a distance of approximately 3 cm to the light source) for 36 h. Afterwards, the mixture was diluted with CH_2Cl_2 and purified by flash chromatography (EtOAc/*n*-hexane = 1:5-1:3) to afford **3n** (45.72 mg, yield: 98%) as a white solid. Enantiomeric excess was established by HPLC analysis using a Chiralpak IC column, ee = 94% (HPLC: IC, 254 nm, *n*-hexane/isopropanol = 85:15, flow rate 1.0 mL/min, 25 °C, t_r(minor) = 21.74 min, t_r(major) = 38.86 min). $[\alpha]_D^{20} = -28.81°$ (c = 1.0, $CHCl_3$).

^{1}H NMR (500 MHz, $CDCl_3$): δ (ppm) 7.46-7.36 (m, 3H), 7.26-7.13 (m, 12H), 6.88-6.76 (m, 2H), 6.24 (dd, J = 8.4, 6.6 Hz, 1H), 3.92 (dd, J = 15.6, 6.4 Hz, 1H), 3.80 (dt, J = 16.4, 10.0 Hz, 2H), 3.58 (dd, J = 15.5, 8.7 Hz, 1H), 0.92-0.83 (m, 1H), 0.79-0.61 (m, 6H).

^{13}C NMR (126 MHz, $CDCl_3$): δ (ppm) 188.3, 156.1, 143.0, 139.8, 138.4, 130.2, 129.7, 129.0, 128.7,

128.5, 128.4, 128.4, 128.3, 127.7, 127.4, 127.2, 125.9, 71.8, 57.2, 41.7, 27.9, 19.0.

IR (film): *ν* (cm^{-1}) 2961, 2925, 2854, 1692, 1596, 1493, 1447, 1305, 1261, 1098, 1019, 824, 699, 559.

HRMS (ESI, *m/z*) calcd for $C_{29}H_{29}N_3NaO_3$ $(M+Na)^+$: 490.2101, found: 490.2106.

Compound 3o

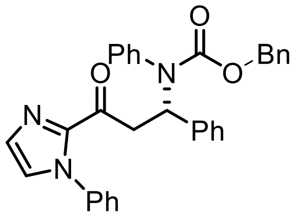

To a solution of α,β-unsaturated 2-acyl imidazole **1a** (32.90 mg, 0.12 mmol) in fresh distilled CH_2Cl_2 (1.0 mL, 0.1 M) was added rhodium (III) complex Δ-**RhO** (4.15 mg, 0.005 mmol, 5 mol%), **PC1** (2.29 mg, 2 mol%), **B2** (9.83 mg, 20 mol%), 4 Å MS (10.0 mg, 100 mg/mmol) in a 10 mL Schlenk tube and the resulting solution was stirred at room temperature for 15 min. Then, carbamate **2g** (22.70 mg, 0.10 mmol) was added and the solution was bubbled for 15 min with argon. The reaction was stirred in a thermostatic cabinet (the actual temperature was about 22-25 °C) under blue LEDs (24 W+36 W) irradiation (the Schlenk tube was kept at a distance of approximately 3 cm to the light source) for 36 h. Afterwards, the mixture was diluted with CH_2Cl_2 and purified by flash chromatography (EtOAc/*n*-hexane = 1:5-1:3) to afford **3o** (42.62 mg, yield: 85%) as a white solid. Enantiomeric excess was established by HPLC analysis using a Chiralpak IA column, ee = 94% (HPLC: IA, 254 nm, *n*-hexane/isopropanol = 90:10, flow rate 1.0 mL/min, 25 °C, t_r(major) = 23.43 min, t_r(minor) = 29.26 min). $[\alpha]_D^{20}$ = −2.58° (*c* = 1.0, $CHCl_3$).

^1H NMR (500 MHz, CD_2Cl_2): *δ* (ppm) 7.39-7.29 (m, 3H), 7.25-7.09 (m, 15H), 7.04 (d, *J* = 7.0 Hz, 2H), 6.76 (d, *J* = 6.1 Hz, 2H), 6.08 (t, *J* = 7.5 Hz, 1H), 4.99 (q, *J* = 12.8 Hz, 2H), 3.79 (dd, *J* = 16.0, 6.8 Hz, 1H), 3.54 (dd, *J* = 16.0, 8.4 Hz, 1H).

^{13}C NMR (126 MHz, CD_2Cl_2): δ (ppm) 188.3, 155.6, 143.0, 139.9, 138.6, 137.0, 130.1, 129.7, 129.0, 128.7, 128.6, 128.5, 128.4, 128.3, 127.8, 127.7, 127.6, 127.5, 127.4, 127.2, 126.0, 67.1, 57.5, 41.5.

IR (film): ν (cm^{-1}) 2961, 2920, 2851, 1631, 1408, 1260, 1093, 1019, 829, 559.

HRMS (ESI, *m/z*) calcd for $C_{32}H_{27}N_3NaO_3$ $(M+Na)^+$: 524.1945, found: 524.1949.

Compound 3p

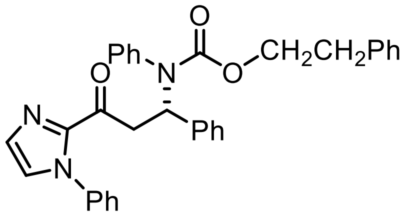

To a solution of α,β-unsaturated 2-acyl imidazole **1a** (32.90 mg, 0.12 mmol) in fresh distilled CH_2Cl_2 (1.0 mL, 0.1 M) was added rhodium (III) complex Δ-**RhO** (4.15 mg, 0.005 mmol, 5 mol%), **PC1** (2.29 mg, 2 mol%), **B2** (9.83 mg, 20 mol%), 4 Å MS (10.0 mg, 100 mg/mmol) in a 10 mL Schlenk tube and the resulting solution was stirred at room temperature for 15 min. Then, carbamate **2h** (24.10 mg, 0.10 mmol) was added and the solution was bubbled for 15 min with argon. The reaction was stirred in a thermostatic cabinet (the actual temperature was about 22-25 °C) under blue LEDs (24 W+36 W) irradiation (the Schlenk tube was kept at a distance of approximately 3 cm to the light source) for 40 h. Afterwards, the mixture was diluted with CH_2Cl_2 and purified by flash chromatography (EtOAc/*n*-hexane = 1:5-1:3) to afford **3p** (39.18 mg, yield: 76%) as a white solid. Enantiomeric excess was established by HPLC analysis using a Chiralpak IC column, ee = 96% (HPLC: IC, 254 nm, *n*-hexane/isopropanol = 85:15, flow rate 1.0 mL/min, 25 °C, t_r(minor) = 33.76 min, t_r(major) = 65.36 min). $[\alpha]_D^{20} = -19.08°$ (*c* = 1.0, $CHCl_3$).

^1H NMR (500 MHz, $CDCl_3$): δ (ppm) 7.47-7.34 (m, 3H), 7.26-7.11 (m, 15H), 6.94 (d, *J* = 7.7 Hz, 2H), 6.75 (d, *J* = 7.4 Hz, 2H), 6.21 (t, *J* = 7.4 Hz, 1H), 4.21 (m, 2H), 3.89 (dd, *J* = 15.6, 6.5 Hz, 1H), 3.57 (dd, *J* = 15.6, 8.7 Hz, 1H), 2.75 (m, 2H).

^{13}C NMR (126 MHz, $CDCl_3$): δ (ppm) 187.2, 154.7, 141.9, 138.5, 137.2, 137.1, 129.2, 128.6, 128.0, 127.9, 127.67, 127.4, 127.3, 127.2, 127.2, 126.7, 126.4, 126.3, 126.1, 125.2, 124.8, 65.2, 56.2, 40.5, 34.3.

IR (film): ν (cm^{-1}) 2962, 2918, 2849, 1595, 1493, 1446, 1403, 1303, 1261, 1089, 1019, 799, 698.

HRMS (ESI, *m/z*) calcd for $C_{33}H_{29}N_3NaO_3$ $(M+Na)^+$: 538.2101, found: 538.2104.

Compound 3q

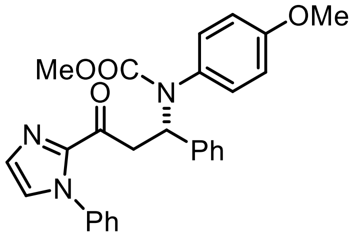

To a solution of α,β-unsaturated 2-acyl imidazole **1a** (32.90 mg, 0.12 mmol) in fresh distilled CH_2Cl_2 (1.0 mL, 0.1 M) was added rhodium (III) complex Δ-**RhO** (4.15 mg, 0.005 mmol, 5 mol%), **PC1** (2.29 mg, 2 mol%), **B2** (9.83 mg, 20 mol%), 4 Å MS (10.0 mg, 100 mg/mmol) in a 10 mL Schlenk tube and the resulting solution was stirred at room temperature for 15 min. Then, carbamate **2i** (18.10 mg, 0.10 mmol) was added and the solution was bubbled for 15 min with argon. The reaction was stirred in a thermostatic cabinet (the actual temperature was about 22-25 °C) under blue LEDs (24 W+36 W) irradiation (the Schlenk tube was kept at a distance of approximately 3 cm to the light source). After 18 h, the mixture was monitored by crude ^1H NMR. Compound **3q** was not formed.

Compound 3r

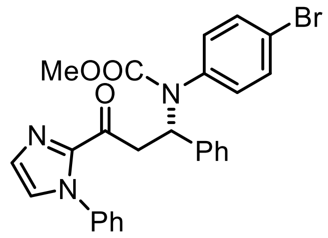

To a solution of α,β-unsaturated 2-acyl imidazole **1a** (32.90 mg, 0.12 mmol) in fresh distilled CH_2Cl_2 (1.0 mL, 0.1 M) was added rhodium (III) complex Δ-**RhO** (4.15 mg, 0.005 mmol, 5 mol%), **PC1** (2.29 mg, 2 mol%), **B2** (9.83 mg, 20 mol%), 4 Å MS (10.0 mg, 100 mg/mmol) in a 10 mL Schlenk tube and the resulting solution was stirred at room temperature for 15 min. Then, carbamate **2j** (22.90 mg, 0.10 mmol) was added and the solution was bubbled for 15 min with argon. The reaction was stirred in a thermostatic cabinet (the actual temperature was about 22-25 °C) under blue LEDs (24 W+36 W) irradiation (the Schlenk tube was kept at a distance of approximately 3 cm to the light source) for 40 h. Afterwards, the mixture was diluted with CH_2Cl_2 and purified by flash chromatography (EtOAc/*n*-hexane = 1:5-1:3) to afford **3r** (42.22 mg, yield: 84%) as a white solid. Enantiomeric excess was established by HPLC analysis using a Chiralpak IA column, ee = 96% (HPLC: IA, 254 nm, *n*-hexane/isopropanol = 90:10, flow rate 1.0 mL/min, 25 °C, t_r(minor) = 18.01 min, t_r(major) = 21.04 min). $[\alpha]_D^{20}$ = −8.54° (*c* = 1.0, $CHCl_3$).

^{1}H NMR (500 MHz, CD_2Cl_2): δ (ppm) 7.39-7.33 (m, 3H), 7.29-7.22 (m, 2H), 7.21-7.04 (m, 9H), 6.66-6.57 (m, 2H), 6.05 (dd, *J* = 8.8, 6.4 Hz, 1H), 3.78 (dd, *J* = 15.8, 6.3 Hz, 1H), 3.53-3.43 (m, 4H).

^{13}C NMR (126 MHz, CD_2Cl_2): δ (ppm) 188.5, 156.2, 143.2, 139.8, 138.8, 138.2, 132.2, 132.1, 130.0, 129.2, 129.0, 128.7, 128.5, 128.3, 127.8, 126.3, 121.7, 57.6, 53.2, 41.5.

IR (film): ν (cm^{-1}) 2962, 2920, 2850, 1596, 1489, 1444, 1403, 1260, 1095, 1020, 800, 693, 559.

HRMS (ESI, *m*/*z*) calcd for $C_{26}H_{22}BrN_3NaO_3$ $(M+Na)^+$: 526.0737, found: 526.0742.

Compound 3s

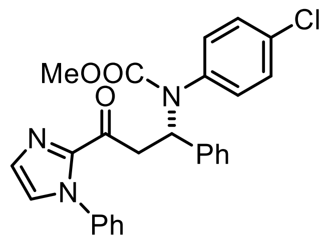

To a solution of α,β-unsaturated 2-acyl imidazole **1a** (32.90 mg, 0.12 mmol) in fresh distilled CH_2Cl_2 (1.0 mL, 0.1 M) was added rhodium (III) complex Δ-**RhO** (4.15 mg, 0.005 mmol, 5 mol%), **PC1** (2.29 mg, 2 mol%), **B2** (9.83 mg, 20 mol%), 4 Å MS (10.0 mg, 100 mg/mmol) in a 10 mL Schlenk tube and the resulting solution was stirred at room temperature for 15 min. Then, carbamate **2k** (18.50 mg, 0.10 mmol) was added and the solution was bubbled for 15 min with argon. The reaction was stirred in a thermostatic cabinet (the actual temperature was about 22-25 °C) under blue LEDs (24 W+36 W) irradiation (the Schlenk tube was kept at a distance of approximately 3 cm to the light source) for 40 h. Afterwards, the mixture was diluted with CH_2Cl_2 and purified by flash chromatography (EtOAc/*n*-hexane = 1:5-1:3) to afford **3s** (40.79 mg, yield: 89%) as a white solid. Enantiomeric excess was established by HPLC analysis using a Chiralpak IA column, ee = 97% (HPLC: IA, 254 nm, *n*-hexane/isopropanol = 90:10, flow rate 1.0 mL/min, 25 °C, t_r(minor) = 16.82 min, t_r(major) = 19.60 min). $[\alpha]_D^{20}$ = −19.89° (*c* = 1.0, $CHCl_3$).

^{1}H NMR (500 MHz, CD_2Cl_2): δ (ppm) 7.39-7.32 (m, 3H), 7.21-7.13 (m, 6H), 7.13-7.06 (m, 5H), 6.71-6.63 (m, 2H), 6.05 (dd, *J* = 8.7, 6.4 Hz, 1H), 3.78 (dd, *J* = 15.8, 6.3 Hz, 1H), 3.53-3.43 (m, 4H).

^{13}C NMR (126 MHz, CD_2Cl_2): δ (ppm) 188.5, 156.3, 143.2, 139.8, 138.8, 137.6, 133.5, 131.9, 130.0, 129.2, 129.0, 129.0, 128.7, 128.5, 128.2, 127.8, 126.3, 57.7, 53.2, 41.5.

IR (film): ν (cm^{-1}) 2961, 2920, 2850, 1681, 1614, 1492, 1443, 1403, 1260, 1091, 1019, 799, 697.

HRMS (ESI, *m*/*z*) calcd for $C_{26}H_{22}ClN_3NaO_3$ $(M+Na)^+$: 482.1242, found: 482.1243.

Compound 3t

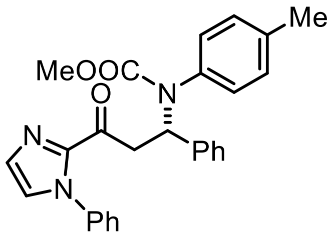

To a solution of α,β-unsaturated 2-acyl imidazole **1a** (32.90 mg, 0.12 mmol) in fresh distilled CH_2Cl_2 (1.0 mL, 0.1 M) was added rhodium (III) complex Δ-**RhO** (4.15 mg, 0.005 mmol, 5 mol%), **PC1** (2.29 mg, 2 mol%), **B2** (9.83 mg, 20 mol%), 4 Å MS (10.0 mg, 100 mg/mmol) in a 10 mL Schlenk tube and the resulting solution was stirred at room temperature for 15 min. Then, carbamate **2l** (16.50 mg, 0.10 mmol) was added and the solution was bubbled for 15 min with argon. The reaction was stirred in a thermostatic cabinet (the actual temperature was about 22-25 °C) under blue LEDs (24 W+36 W) irradiation (the Schlenk tube was kept at a distance of approximately 3 cm to the light source) for 18 h. Afterwards, the mixture was diluted with CH_2Cl_2 and purified by flash chromatography (EtOAc/*n*-hexane = 1:5-1:3) to afford **3t** (39.02 mg, yield: 89%) as a white solid. Enantiomeric excess was established by HPLC analysis using a Chiralpak IA column, ee = 99% (HPLC: IA, 254 nm, *n*-hexane/isopropanol = 90:10, flow rate 1.0 mL/min, 25 °C, t_r(minor) = 15.69 min, t_r(major) = 17.53 min). $[\alpha]_D^{20}$ = −25.21° (*c* = 1.0, $CHCl_3$).

^{1}H NMR (500 MHz, CD_2Cl_2): δ (ppm) 7.42-7.29 (m, 3H), 7.21-7.06 (m, 9H), 6.94 (d, *J* = 8.0 Hz, 2H), 6.58 (d, *J* = 8.1 Hz, 2H), 6.04 (t, *J* = 7.5 Hz, 1H), 3.76 (dd, *J* = 16.0, 6.7 Hz, 1H), 3.55-3.41 (m, 4H), 2.22 (s, 3H).

^{13}C NMR (126 MHz, CD_2Cl_2): δ (ppm) 188.3, 156.4, 143.0, 140.0, 138.6, 137.6, 136.0, 129.9, 129.7, 129.3, 129.0, 128.7, 128.4, 128.3, 127.8, 127.5, 126.0, 57.2, 52.8, 41.4, 20.9.

IR (film): ν (cm^{-1}) 2962, 2919, 2950, 1692, 1597, 1512, 1443, 1404, 1306, 1260, 1091, 1019, 799, 697.

HRMS (ESI, *m*/*z*) calcd for $C_{27}H_{25}N_3NaO_3$ $(M+Na)^+$: 462.1788, found: 462.1787.

Compound 3u

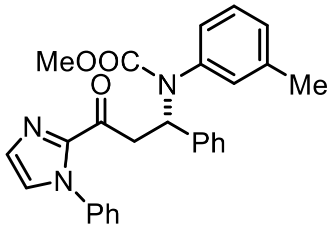

To a solution of α,β-unsaturated 2-acyl imidazole **1a** (32.90 mg, 0.12 mmol) in fresh distilled CH_2Cl_2 (1.0 mL, 0.1 M) was added rhodium (III) complex Δ-**RhO** (4.15 mg, 0.005 mmol, 5 mol%), **PC1** (2.29 mg, 2 mol%), **B2** (9.83 mg, 20 mol%), 4 Å MS (10.0 mg, 100 mg/mmol) in a 10 mL Schlenk tube and the resulting solution was stirred at room temperature for 15 min. Then, carbamate **2m** (16.50 mg, 0.10 mmol) was added and the solution was bubbled for 15 min with argon. The reaction was stirred in a thermostatic cabinet (the actual temperature was about 22-25 °C) under blue LEDs (24 W+36 W) irradiation (the Schlenk tube was kept at a distance of approximately 3 cm to the light source) for 18 h. Afterwards, the mixture was diluted with CH_2Cl_2 and purified by flash chromatography (EtOAc/*n*-hexane = 1:5-1:3) to afford **3u** (39.58 mg, yield: 90%) as a white solid. Enantiomeric excess was established by HPLC analysis using a Chiralpak IA column, ee = 96% (HPLC: IA, 254 nm, *n*-hexane/isopropanol = 90:10, flow rate 1.0 mL/min, 25 °C, t_r(minor) =14.71 min, t_r(major) = 16.19 min). $[\alpha]_D^{20}$ = −13.60° (*c* = 1.0, $CHCl_3$).

^{1}H NMR (500 MHz, CD_2Cl_2): δ (ppm) 7.40-7.32 (m, 3H), 7.21-7.09 (m, 9H), 7.06-6.94 (m, 2H), 6.51 (m, 2H), 6.02 (t, *J* = 7.5 Hz, 1H), 3.74 (dd, *J* = 16.1, 6.7 Hz, 1H), 3.58-3.44 (m, 4H), 2.12 (s, 3H).

^{13}C NMR (126 MHz, CD_2Cl_2): δ (ppm) 188.6, 156.5, 143.3, 140.2, 138.9, 138.8, 131.0, 129.9, 129.2, 129.0, 128.61, 128.57, 128.52, 128.48, 128.0, 127.7, 127.3, 126.2, 57.6, 53.1, 41.7, 21.3.

IR (film): ν (cm^{-1}) 2962, 2917, 2849, 1693, 1443, 1404, 1260, 1092, 1018, 825, 799, 698, 559.

HRMS (ESI, *m*/*z*) calcd for $C_{27}H_{25}N_3NaO_3$ $(M+Na)^+$: 462.1788, found: 462.1788.

Compound 3v

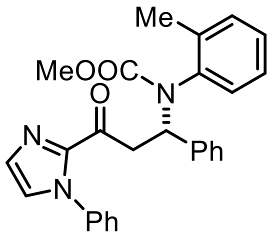

To a solution of α,β-unsaturated 2-acyl imidazole **1a** (32.90 mg, 0.12 mmol) in fresh distilled CH_2Cl_2 (1.0 mL, 0.1 M) was added rhodium (III) complex Δ-**RhO** (4.15 mg, 0.005 mmol, 5 mol%), **PC1** (2.29 mg, 2 mol%), **B2** (9.83 mg, 20 mol%), 4 Å MS (10.0 mg, 100 mg/mmol) in a 10 mL Schlenk tube and the resulting solution was stirred at room temperature for 15 min. Then, carbamate **2n** (16.50 mg, 0.10 mmol) was added and the solution was bubbled for 15 min with argon. The reaction was stirred in a thermostatic cabinet (the actual temperature was about 22-25 °C) under blue LEDs (24 W+36 W) irradiation (the Schlenk tube was kept at a distance of approximately 3 cm to the light source) for 18 h. Afterwards, the mixture was diluted with CH_2Cl_2 and purified by flash chromatography (EtOAc/*n*-hexane = 1:5-1:3) to afford **3v** (40.42 mg, yield: 92%) as a white solid. Enantiomeric excess was established by HPLC analysis using a Chiralpak IA column, ee = 97% (HPLC: IA, 254 nm, *n*-hexane/isopropanol = 90:10, flow rate 1.0 mL/min, 25 °C, t_r(minor) = 13.76 min, t_r(major) = 17.10 min). $[\alpha]_D^{20}$ = −10.14° (*c* = 1.0, $CHCl_3$).

^{1}H NMR (500 MHz, CD_2Cl_2): δ (ppm) 7.37-7.28 (m, 3H), 7.25 (d, *J* = 5.3 Hz, 1H), 7.24-6.99 (m, 10H), 6.93 (dd, *J* = 13.9, 6.5 Hz, 2H), 5.95 (m, 1H), 3.99 (dt, *J* = 13.1, 6.6 Hz, 1H), 3.60 (dd, *J* = 15.5, 7.4 Hz, 1H), 3.49 (s, 3H), 1.94 (s, 1H), 1.32 (s, 2H).（With a rotation ratio = 2:1）

^{13}C NMR (126 MHz, CD_2Cl_2): δ (ppm) 188.4, 143.1, 140.7, 138.7, 138.6, 138.3, 130.8, 130.1, 129.7, 129.1, 128.9, 128.6, 128.4, 128.1, 128.0, 127.8, 127.4, 126.4, 126.0, 58.4, 52.9, 43.2, 17.1.

IR (film): ν (cm^{-1}) 2957, 2918, 2849, 1697, 1492, 1442, 1404, 1305, 1260, 1087, 1022, 800, 764, 699.

HRMS (ESI, *m*/*z*) calcd for $C_{27}H_{25}N_3NaO_3$ $(M+Na)^+$: 462.1788, found: 462.1796.

Compound 3w

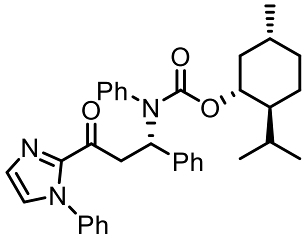

To a solution of α,β-unsaturated 2-acyl imidazole **1a** (32.90 mg, 0.12 mmol) in fresh distilled CH_2Cl_2 (1.0 mL, 0.1 M) was added rhodium (III) complex Δ-**RhO** (4.15 mg, 0.005 mmol, 5 mol%), **PC1** (2.29 mg, 2 mol%), **B2** (9.83 mg, 20 mol%), 4 Å MS (10.0 mg, 100 mg/mmol) in a 10 mL Schlenk tube and the resulting solution was stirred at room temperature for 15 min. Then, carbamate **2o** (27.50 mg, 0.10 mmol) was added and the solution was bubbled for 15 min with argon. The reaction was stirred in a thermostatic cabinet (the actual temperature was about 22-25 °C) under blue LEDs (24 W+36 W) irradiation (the Schlenk tube was kept at a distance of approximately 3 cm to the light source), which was monitored by TLC analysis. Afterwards, the mixture was diluted with CH_2Cl_2 and purified by flash chromatography (EtOAc/*n*-hexane = 1:5-1:3) to afford **3w** (46.72 mg, yield: 85%) as a white solid. Diastereomeric ratio was established by HPLC analysis using a Chiralpak IA column, >99:1 d.r. (HPLC: IA, 254 nm, *n*-hexane/isopropanol = 90:10, flow rate 1.0 mL/min, 25 °C, t_r(minor) = 9.87 min, t_r(major) = 8.96 min). $[\alpha]_D^{20}$ = −23.55° (*c* = 1.0, $CHCl_3$).

^{1}H NMR (500 MHz, $CDCl_3$): δ (ppm) 7.40 (dd, *J* = 9.8, 5.3 Hz, 3H), 7.26-7.14 (m, 12H), 6.80 (s, 2H), 6.23 (t, 1H), 4.57-4.44 (m, 1H), 3.95-3.85 (m, 1H), 3.57 (dd, *J* = 15.5, 8.8 Hz, 1H), 2.01 (d, *J* = 8.2 Hz, 1H), 1.42 (d, *J* = 2.7 Hz, 1H), 1.16-1.03 (m, 1H), 0.97 (dd, *J* = 22.9, 13.0 Hz, 2H), 0.86 (dd, 5H), 0.80-0.71 (m, 3H), 0.68 (dd, *J* = 12.5, 7.1 Hz, 5H).

^{13}C NMR (126 MHz, $CDCl_3$): δ (ppm) 188.5, 155.9, 143.1, 140.9, 140.2, 138.5, 130.3, 129.8, 129.1, 128.8, 128.5, 128.3, 127.7, 127.3, 126.0, 75.9, 57.3, 47.2, 41.7, 41.3, 34.5, 31.6, 26.2, 22.3, 20.8, 16.6, 14.3.

IR (film): ν (cm^{-1}) 2957, 2924, 2867, 2358, 1689, 1596, 1493, 1448, 1404, 1328, 1303, 1262, 1205, 1179, 1097, 1035, 1018, 980, 964, 913, 800, 763, 699, 599, 559.

HRMS (ESI, *m*/*z*) calcd for $C_{35}H_{40}N_3O_3$ $(M+H)^+$: 550.3064, found: 550.3068.

Compound 3x

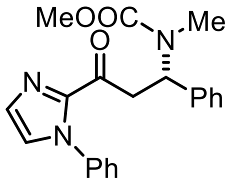

To a solution of α,β-unsaturated 2-acyl imidazole **1a** (32.90 mg, 0.12 mmol) in fresh distilled CH_2Cl_2 (1.0 mL, 0.1 M) was added rhodium (III) complex Δ-**RhO** (4.15 mg, 0.005 mmol, 5 mol%), **PC1** (2.29 mg, 2 mol%), **B2** (9.83 mg, 20 mol%), 4 Å MS (10.0 mg, 100 mg/mmol) in a 10 mL Schlenk tube and the resulting solution was stirred at room temperature for 15 min. Then, carbamate **2p** (8.90 mg, 0.10 mmol) was added and the solution was bubbled for 15 min with argon atmosphere. The reaction was stirred in a thermostatic cabinet (the actual temperature was about 22-25 °C) under blue LEDs (24 W+36 W) irradiation (the Schlenk tube was kept at a distance of approximately 3 cm to the light source). After 18 h, the mixture was monitored by crude ^{1}H NMR. The compound **3x** was not formed (TLC and crude ^{1}H NMR analysis). The main product was a cyclobutane side product (32% yield by crude ^{1}H NMR analysis).

Compound 3y

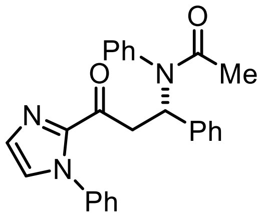

To a solution of α,β-unsaturated 2-acyl imidazole **1a** (32.90 mg, 0.12 mmol) in fresh distilled CH_2Cl_2 (1.0 mL, 0.1 M) was added rhodium (III) complex Δ-**RhO** (4.15 mg, 0.005 mmol, 5 mol%), **PC1** (2.29 mg, 2 mol%), **B2** (9.83 mg, 20 mol%), 4 Å MS (10.0 mg, 100 mg/mmol) in a 10 mL Schlenk tube and the resulting solution was stirred at room temperature for 15 min. Then, carbamate **2q** (13.50 mg, 0.10 mmol) was added and the solution was bubbled for 15 min with argon. The reaction was stirred in a thermostatic cabinet (the actual temperature was about 22-25 °C)

under blue LEDs (24 W+36 W) irradiation (the Schlenk tube was kept at a distance of approximately 3 cm to the light source). After 18 h, the mixture was monitored by ^{1}H NMR. The compound **3y** was not formed. The main product was a cyclobutane side product (28% yield by crude ^{1}H NMR analysis).

Compound 3z

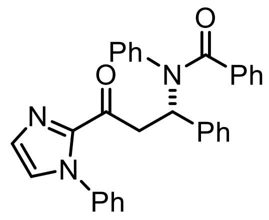

To a solution of α,β-unsaturated 2-acyl imidazole **1a** (32.90 mg, 0.12 mmol) in fresh distilled CH_2Cl_2 (1.0 mL, 0.1 M) was added rhodium (III) complex Δ-**RhO** (4.15 mg, 0.005 mmol, 5 mol%), **PC1** (2.29 mg, 2 mol%), **B2** (9.83 mg, 20 mol%), 4 Å MS (10.0 mg, 100 mg/mmol) in a 10 mL Schlenk tube and the resulting solution was stirred at room temperature for 15 min. Then, carbamate **2r** (19.71 mg, 0.10 mmol) was added and the solution was bubbled for 15 min with argon. The reaction was stirred in a thermostatic cabinet (the actual temperature was about 22-25 °C) under blue LEDs (24 W+36 W) irradiation (the Schlenk tube was kept at a distance of approximately 3 cm to the light source). After 18 h, the mixture was monitored by ^{1}H NMR. The compound **3z** was only formed in small amounts (6% yield according to crude ^{1}H NMR analysis) and not isolated. The main product was a cyclobutane side product (23% yield by crude ^{1}H NMR analysis).

5. Control Experiments

5.1 Control experiments with a strong base

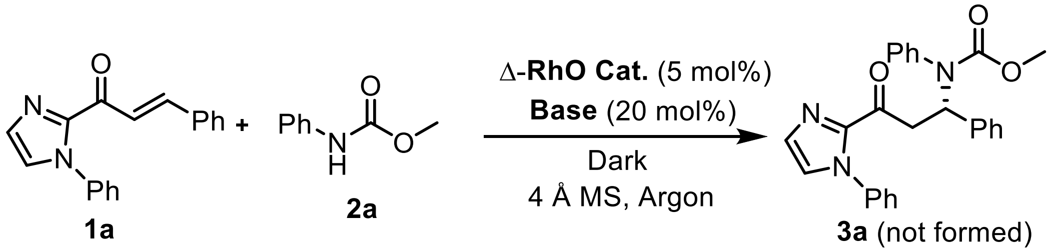

General procedure: To a solution of α,β-unsaturated 2-acyl imidazole **1a** (32.90 mg, 0.12 mmol) in fresh distilled CH_2Cl_2 (1.0 mL) was added rhodium (III) complex Δ-**RhO** (4.15 mg, 0.005 mmol) 4 Å MS (10.0 mg, 100 mg/mmol) in a 10 mL Schlenk tube and the resulting solution was stirred in a thermostatic cabinet (the temperature was set at 20 °C) for 30 min. EtONa (1.46 mg, 0.02 mmol) [20% in EtOH] and carbamate **2a** (15.10 mg, 0.10 mmol) was added to the solution. The resulting solution was stirred at room temperature for 18 h under dark. Result: compound **3a** was not formed (TLC and crude ^{1}H NMR analysis).

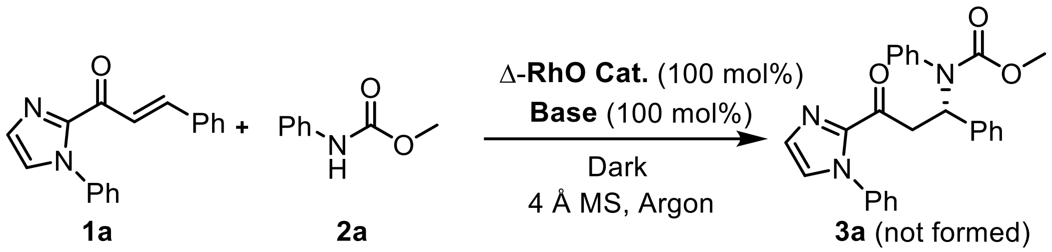

General procedure: To a solution of α,β-unsaturated 2-acyl imidazole **1a** (13.70 mg, 0.05 mmol) in fresh distilled CH_2Cl_2 (0.5 mL) was added rhodium (III) complex Δ-**RhO** (41.5 mg, 0.05 mmol) 4 Å MS (5.0 mg, 100 mg/mmol) in a 10 mL Schlenk tube and the resulting solution was stirred in a thermostatic cabinet (the temperature was set at 20 °C) for 30 min. To a solution of EtONa (3.40 mg, 0.05 mmol) [20% in EtOH] was added carbamate **2a** (7.50 mg, 0.05 mmol) and the resulting solution was stirred at 50 °C for 30 min with argon atmosphere. After cooling to room temperature, the predeprotonated carbamate's EtOH solution was added to rhodium (III) bounded α,β-unsaturated 2-acyl imidazole **1a**. The resulting solution was stirred in a thermostatic cabinet for 18 h under dark. Result: compound **3a** was not formed (TLC and crude ^{1}H NMR analysis).

5.2 Control experiment with electron-rich alkene to capture *N*-radical

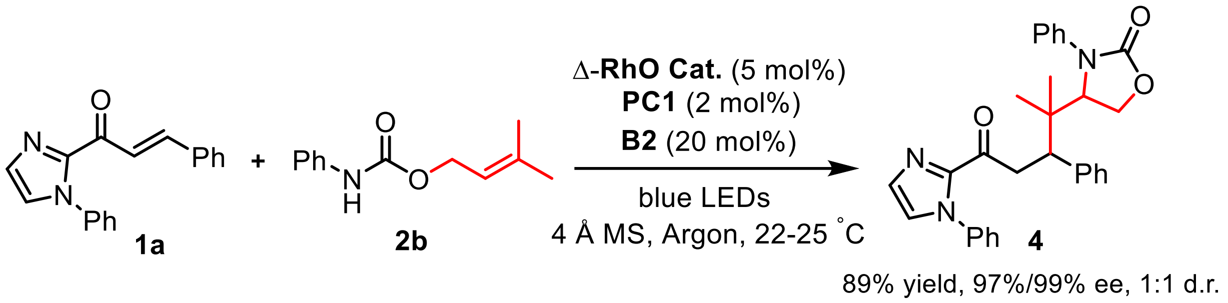

Compound 4

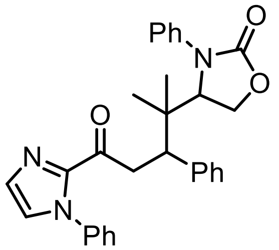

To a solution of α,β-unsaturated 2-acyl imidazole **1a** (32.90 mg, 0.12 mmol) in fresh distilled CH_2Cl_2 (1.0 mL, 0.1 M) was added rhodium (III) complex Δ-**RhO** (4.15 mg, 0.005 mmol, 5 mol%), **PC1** (2.29 mg, 2 mol%), **B2** (9.83 mg, 20 mol%), 4 Å MS (10.0 mg, 100 mg/mmol) in a 10 mL Schlenk tube and the resulting solution was stirred at room temperature for 15 min. Then, carbamate **2b** (20.51 mg, 0.10 mmol) was added and the solution was bubbled for 15 min with argon. The reaction was stirred in a thermostatic cabinet (the actual temperature was about 22-25 °C) under blue LEDs (24 W+36 W) irradiation (the Schlenk tube was kept at a distance of approximately 3 cm to the light source) for 24 h. Afterwards, the mixture was diluted with CH_2Cl_2 and purified by flash chromatography (EtOAc/*n*-hexane = 1:5-1:3) to afford **4** (42.60 mg, yield: 89%) as a white solid. Enantiomeric excess and diastereomeric ratio were established by HPLC analysis using a Chiralpak IA column, ee = 97%/99% dr = 1:1 (HPLC: IA, 254 nm, *n*-hexane/isopropanol = 90:10, flow rate 1.0 mL/min, 25 °C, Diastereomer 1 : t_r(minor) = 41.43 min, t_r(major) = 26.78 min; Diastereomer 2 : t_r(minor) = 74.84 min, t_r(major) = 32.66 min). $[\alpha]_D^{20}$ = −15.60° (*c* = 1.0, $CHCl_3$).

^{1}H NMR (500 MHz, $CDCl_3$): δ (ppm) 7.34-7.29 (m, 5H), 7.17-7.09 (m, 7H), 7.02 (m, 3H),

6.74-6.68 (m, 2H), 4.55 (dd, *J* = 8.1, 3.7 Hz, 1H), 4.50 (dd, *J* = 9.1, 3.0 Hz, 1H), 3.99 (dd, *J* = 8.7, 2.9 Hz, 1H), 3.72 (dd, *J* = 16.5, 10.3 Hz, 1H), 3.53 (dd, *J* = 16.3, 11.1 Hz, 1H), 3.23 (dd, *J* = 16.5, 4.0 Hz, 1H), 0.65 (d, *J* = 12.5 Hz, 6H).

^{13}C NMR (126 MHz, $CDCl_3$): δ (ppm) 188.6, 156.0, 142.0, 139.2, 138.3, 137.0, 128.6, 128.1, 127.9, 127.6, 127.1, 126.0, 126.0, 124.9, 124.5, 123.0, 63.3, 61.8, 46.1, 41.4, 38.8, 21.2, 18.6.

IR (film): ν (cm^{-1}) 2961, 2916, 2849, 1594, 1493, 1446, 1403, 1303, 1261, 1089, 1018, 800, 698, 525.

HRMS (ESI, *m/z*) calcd for $C_{30}H_{30}N_3O_3$ $(M+H)^+$: 480.2282, found: 480.2283.

5.3 Control experiment without Δ-RhO catalyst

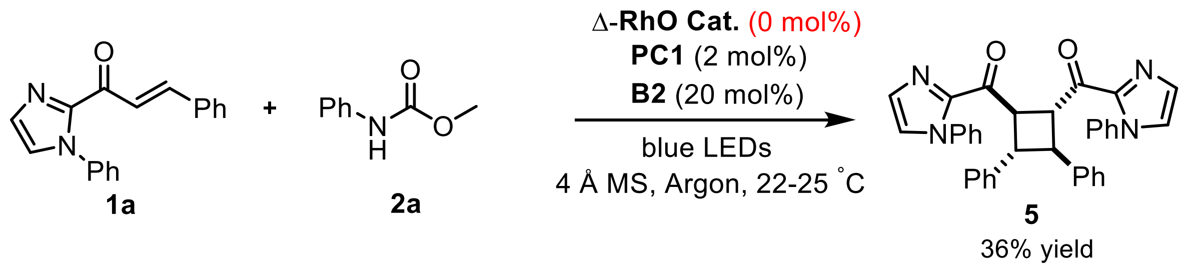

Compound 5

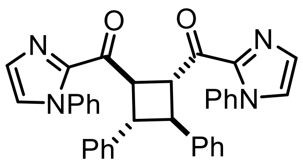

To a solution of α,β-unsaturated 2-acyl imidazole **1a** (27.40 mg, 0.1 mmol) in fresh distilled CH_2Cl_2 (1.0 mL, 0.1 M) was added rhodium (III) complex Δ-**RhO** (4.15 mg, 0.005 mmol, 5 mol%), **PC1** (2.29 mg, 2 mol%), **B2** (9.83 mg, 20 mol%), 4 Å MS (10.0 mg, 100 mg/mmol) in a 10 mL Schlenk tube and the resulting solution was stirred at room temperature for 15 min then purged with argon for 15 min. The reaction was stirred in a thermostatic cabinet (the actual temperature was about 22-25 °C) under blue LEDs (24 W+36 W) irradiation (the Schlenk tube was kept at a distance of approximately 3 cm to the light source) for 18 h. Afterwards, the mixture was diluted with CH_2Cl_2 and purified by flash chromatography (EtOAc/*n*-hexane = 1:5-1:3) to afford **5** (9.86 mg, yield: 36%)

as a white solid. (only one diastereomer was formed, >25:1 d.r. from ^{1}H NMR analysis)

^{1}H NMR (500 MHz, CD_2Cl_2): δ (ppm) 7.57-7.44 (m, 5H), 7.37-7.31 (m, 2H), 7.27 (t, J = 7.5 Hz, 2H), 7.19 (t, J = 7.2 Hz, 1H), 7.12 (s, 1H), 6.98 (s, 1H), 4.65 (dd, J = 5.7, 3.5 Hz, 1H), 4.06 (dd, J = 5.8, 3.5 Hz, 1H).

^{13}C NMR (126 MHz, CD_2Cl_2): δ (ppm) 189.2, 143.3, 142. 5, 139.0, 129.8, 129.3, 129.0, 128.7, 127.6, 127.4, 127.0, 126.3, 50. 5, 44.5.

IR (film): ν (cm^{-1}) 3410, 3059, 3028, 2962, 2919, 2849, 1678, 1650, 1597, 1492, 1419, 1416, 1310, 1260, 1073, 1032, 975, 893, 791, 759, 697, 666.

HRMS (ESI, *m*/*z*) calcd for $C_{36}H_{29}N_4O_2$ $(M+H)^+$: 549.2285, found: 549.2286

5.4 Control experiment with TEMPO as an additive

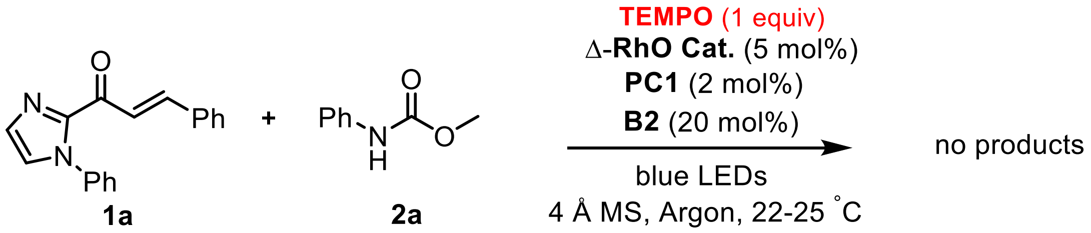

To a solution of α,β-unsaturated 2-acyl imidazole **1a** (32.90 mg, 0.12 mmol) in fresh distilled CH_2Cl_2 (1.0 mL, 0.1 M) was added rhodium (III) complex Δ-**RhO** (4.15 mg, 0.005 mmol, 5 mol%), **PC1** (2.29 mg, 2 mol%), **B2** (9.83 mg, 20 mol%), 4 Å MS (10.0 mg, 100 mg/mmol) in a 10 mL Schlenk tube and the resulting solution was stirred at room temperature for 15 min. Then, carbamate **2a** (15.10 mg, 0.10 mmol) and TEMPO (15.62 mg, 0.1 mmol) were added and the solution was bubbled for 15 min with argon. The reaction was stirred in a thermostatic cabinet (the actual temperature was about 22-25 °C) under blue LEDs (24 W+36 W) irradiation (the Schlenk tube was kept at a distance of approximately 3 cm to the light source) for 18 h. Result: no product formed (TLC and crude ^{1}H NMR analysis).

6. Follow-up Reaction of the Radical Coupling Product 3m

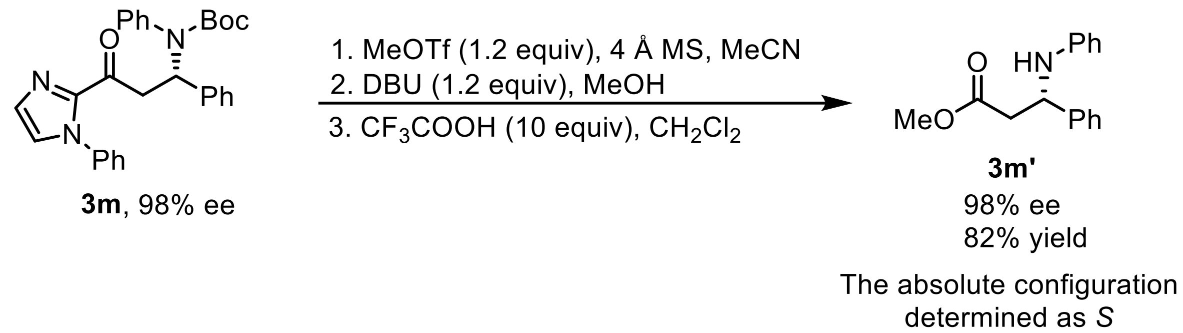

Scheme S2. Synthesis of chiral amino ester derivative.

General procedure: To a solution of radical-radical coupling product **3m** (160 mg, 0.34 mmol, 1.0 equiv) in fresh distilled MeCN (3.4 mL, 0.1 M) was added 4 Å MS (340.0 mg, 100 mg/mmol) in a brown Schlenk tube and the resulting solution was stirred at room temperature for 30 min. MeOTf (67.3 mg, 0.41 mmol, 1.2 equiv) was added to the solution and stirred at room temperature for another 30 min. After cooling to 0 °C, DBU (62.11 mg, 0.41 mmol, 1.2 equiv) in MeOH (2 mL) was added dropwise at 0 °C. The resulting solution was stirred at 0 °C for 30 min. Organic solvent was removed in *vacuo*. The resulting residue was dissolved in fresh distilled CH_2Cl_2 (3.4 mL, 0.1 M). CF_3COOH (387.7 mg, 3.4 mmol) was added in one portion. After stirring at room temperature for 2 h. The solvent was removed in *vacuo* and the resulting residue was partitioned between saturated aqueous $NaHCO_3$ and CH_2Cl_2 (2 x 5 ml). The organic layers were combined and the volatiles were removed in *vacuo*. The residue was purified by flash chromatography (EtOAc/*n*-hexane = 1:5-1:3) to afford **3m'** (71.30 mg, yield: 82%) as a white solid.[8] Enantiomeric excess was established by HPLC analysis using a Chiralpak AD-H column, *ee* = 98% (HPLC: AD-H, 254 nm, *n*-hexane/isopropanol = 95:5, flow rate 1.0 mL/min, 25 °C, t_r(minor) = 9.74 min, t_r(major) = 11.12 min). $[\alpha]_D^{20}$ = +23.78° (*c* = 1.0, $CHCl_3$). The absolute configuration was assigned as ***S*** by comparing the optical rotation.[8]

^{1}H NMR (400 MHz, $CDCl_3$): δ (ppm) 7.43-7.28 (m, 4H), 7.24 (t, J = 4.7 Hz, 1H), 7.14-7.06 (m, 2H), 6.71-6.63 (m, 1H), 6.61-6.49 (m, 2H), 4.84-4.53 (m, 2H), 3.65 (s, 3H), 2.89-2.74 (m, 2H).

All other data were in agreement with the published literature.[8]

7. Chiral Chromatography

Optical purities of the compounds **3a-g**, **3i**, **3k-p**, **3r-w**, **4**, and **3m'** were determined with a Daicel Chiralpak IA and IC HPLC column on an Agilent 1260 Series HPLC System. UV-absorption was measured at 254 nm.

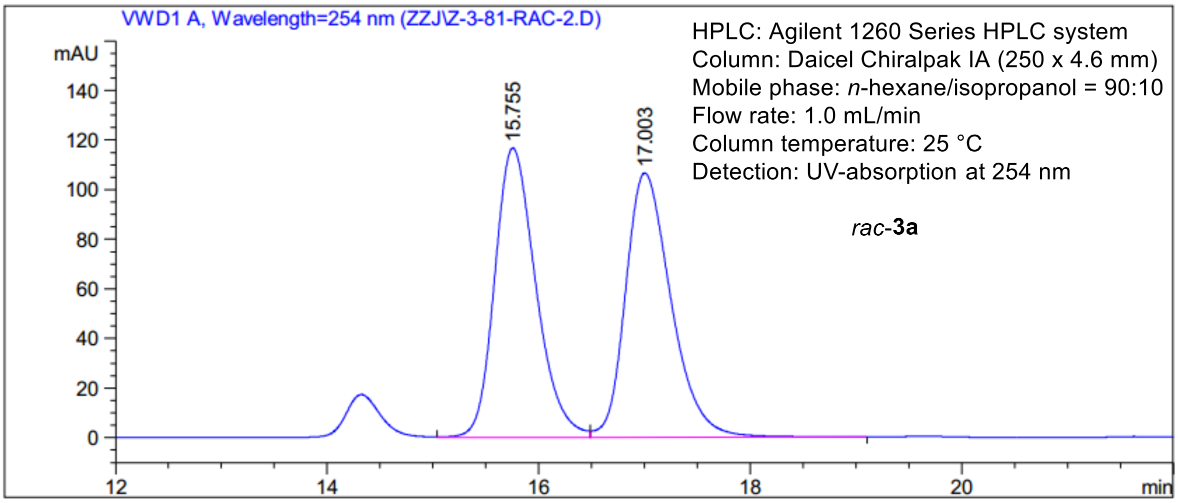

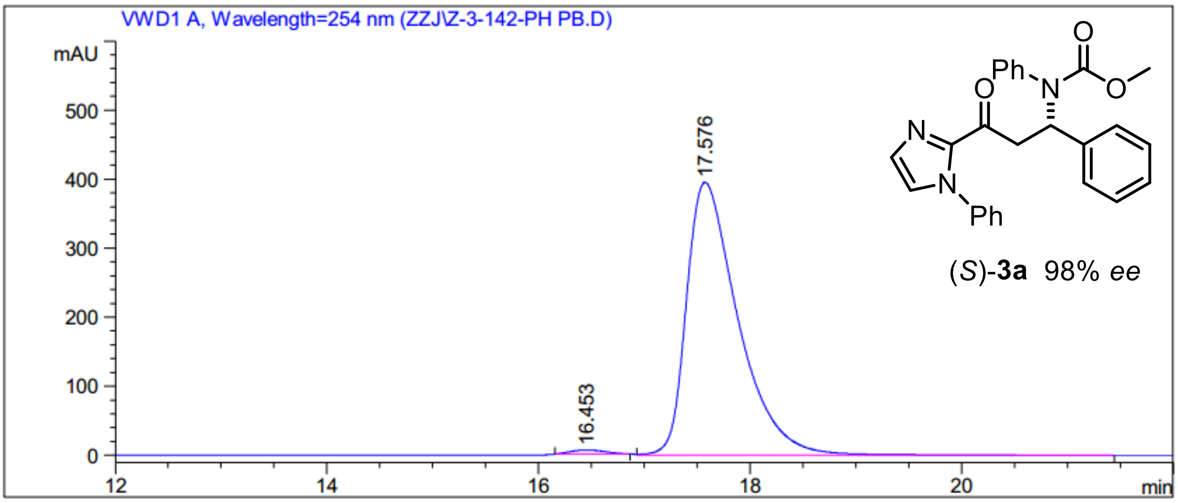

#	[min]		[min]	[mAU*s]	[mAU]	%
1	16.453	MP R	0.3805	137.35995	6.01738	1.0130
2	17.576	VV	0.5122	1.34228e4	395.21924	98.9870

Figure S1. HPLC trace for the racemic reference *rac*-**3a**, and non-racemic product (*S*)-**3a** generated from the asymmetric reaction catalyzed by Δ-**RhO**.

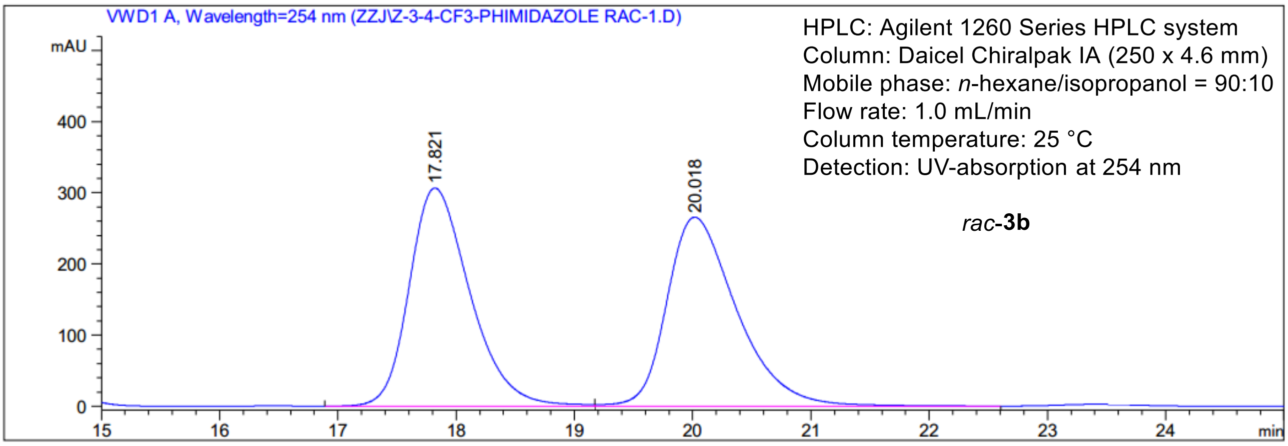

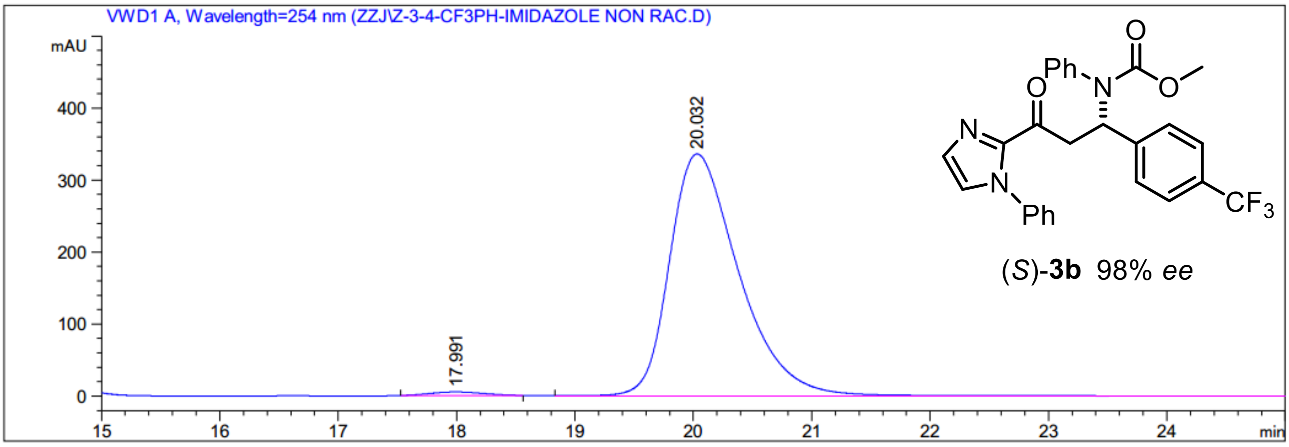

#	[min]		[min]	[mAU*s]	[mAU]	%
1	17.991	PM R	0.4023	158.13922	5.05491	1.1411
2	20.032	VB	0.6236	1.36998e4	336.07904	98.8589

Figure S2. HPLC trace for the racemic reference *rac*-**3b**, and non-racemic product (*S*)-**3b** generated from the asymmetric reaction catalyzed by Δ-**RhO**.

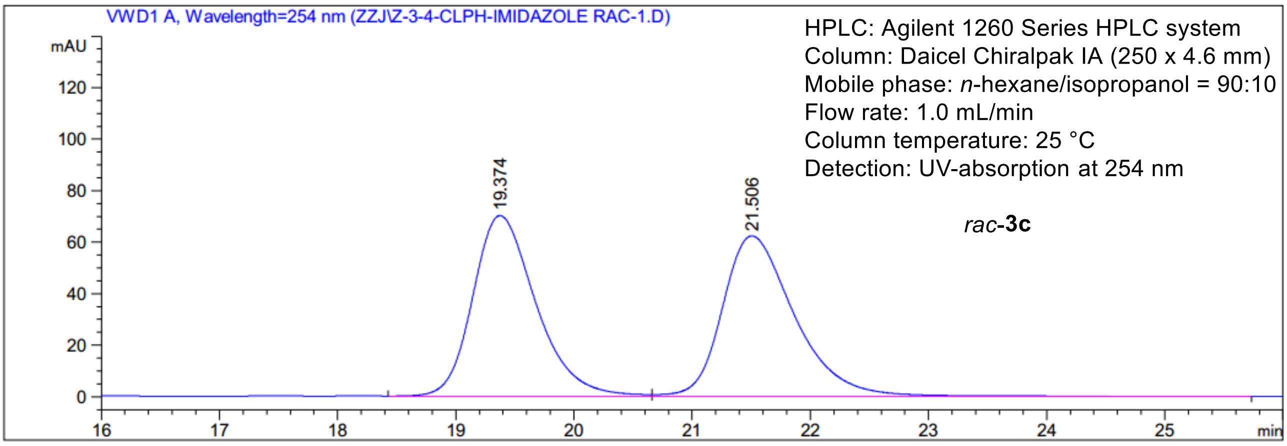

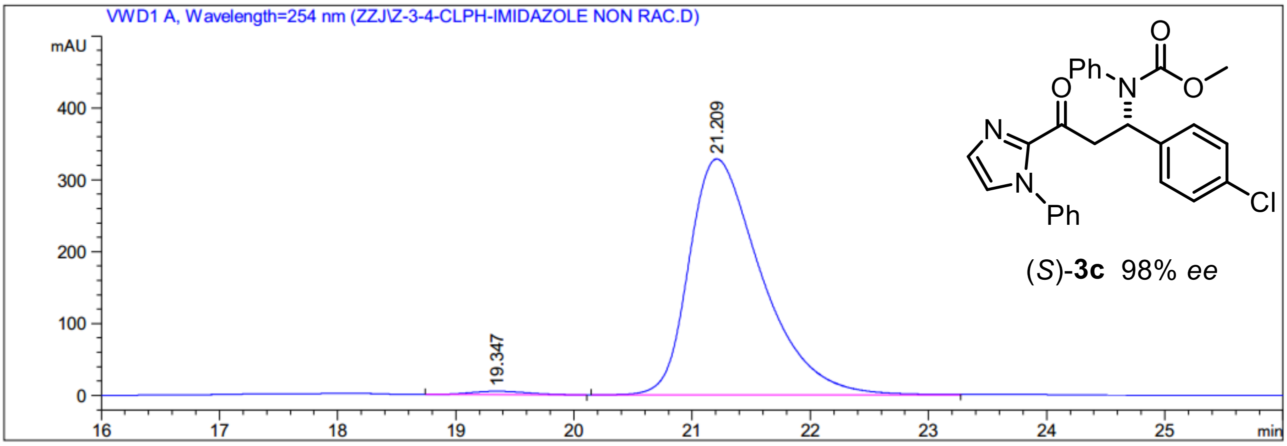

#	[min]		[min]	[mAU*s]	[mAU]	%
1	19.347	BB	0.5325	161.84428	4.81019	1.1385
2	21.209	BV	0.6515	1.40542e4	328.26813	98.8615

Figure S3. HPLC trace for the racemic reference *rac*-**3c**, and non-racemic product (*S*)-**3c** generated from the asymmetric reaction catalyzed by Δ-**RhO**.

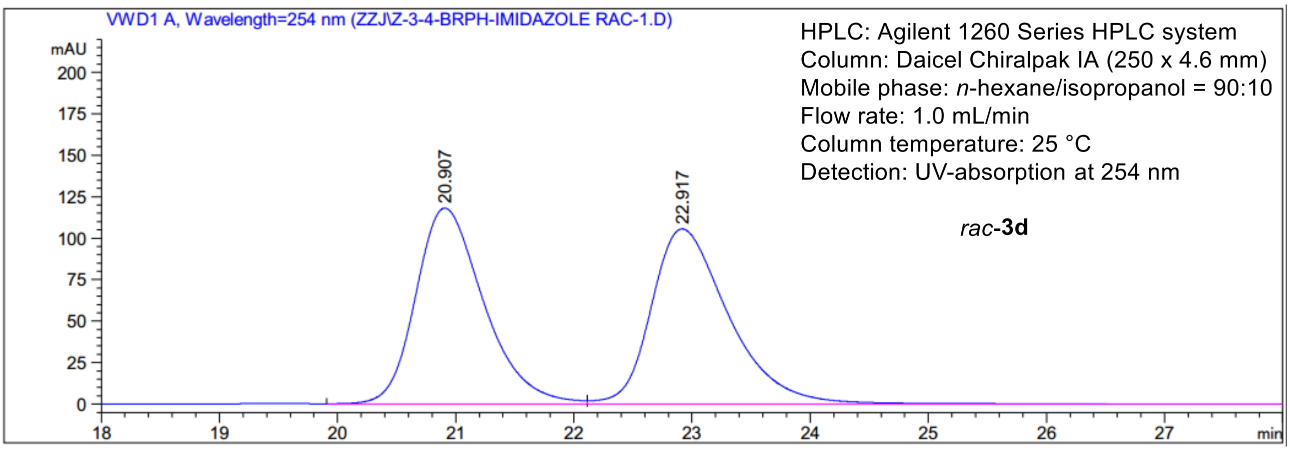

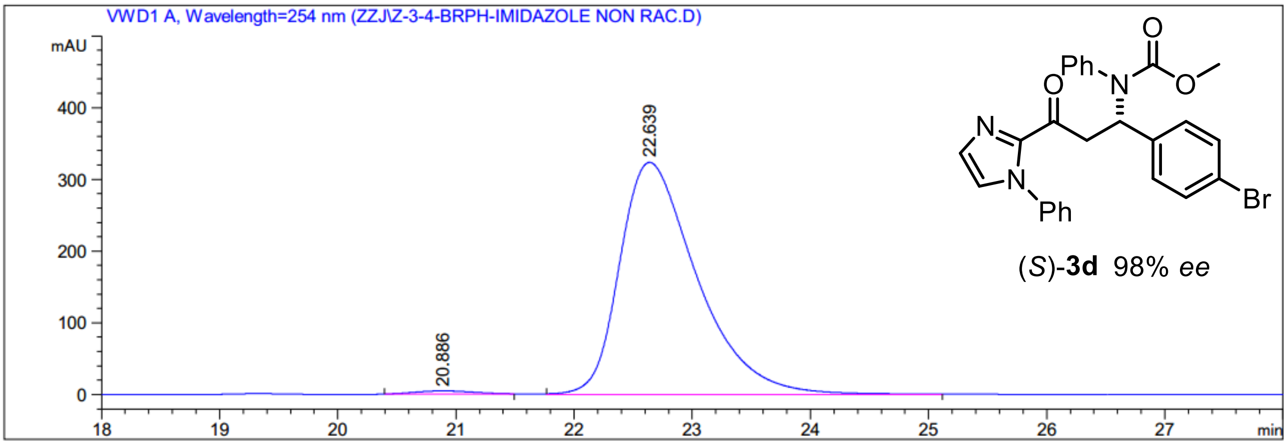

#	[min]		[min]	[mAU*s]	[mAU]	%
1	20.886	MM R	0.5409	154.45181	4.51117	1.0323
2	22.639	MM R	0.5268	1.48080e4	323.59824	98.9677

Figure S4. HPLC trace for the racemic reference *rac*-**3d**, and non-racemic product (*S*)-**3d** generated from the asymmetric reaction catalyzed by Δ-**RhO**.

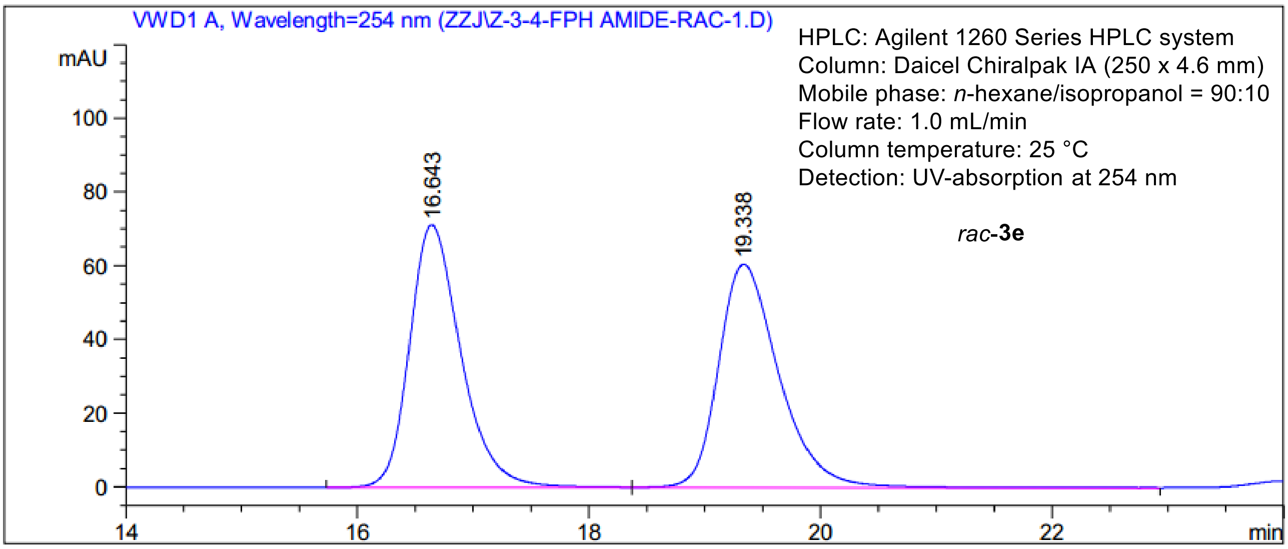

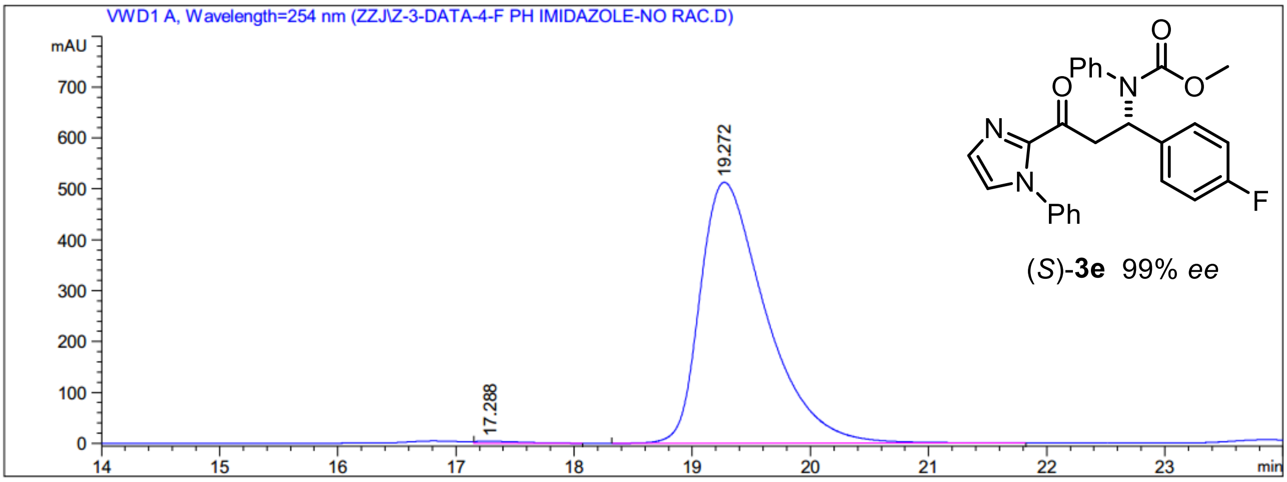

#	[min]		[min]	[mAU*s]	[mAU]	%
1	17.288	MP R	0.3554	93.29623	3.52924	0.4697
2	19.272	BB	0.5820	1.97677e4	512.51788	99.5303

Figure S5. HPLC trace for the racemic reference *rac*-**3e**, and non-racemic product (*S*)-**3e** generated from the asymmetric reaction catalyzed by Δ-**RhO**.

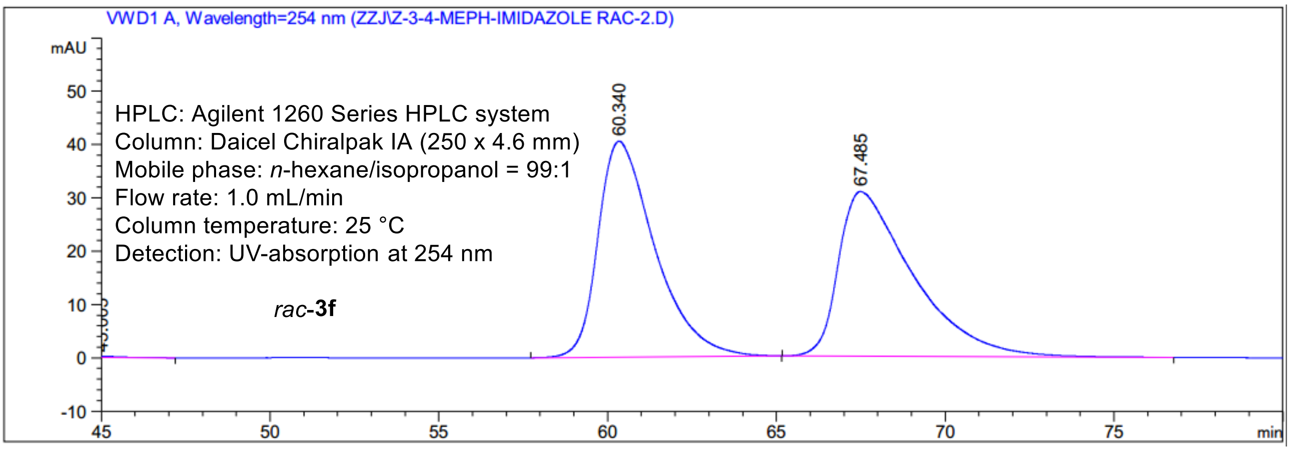

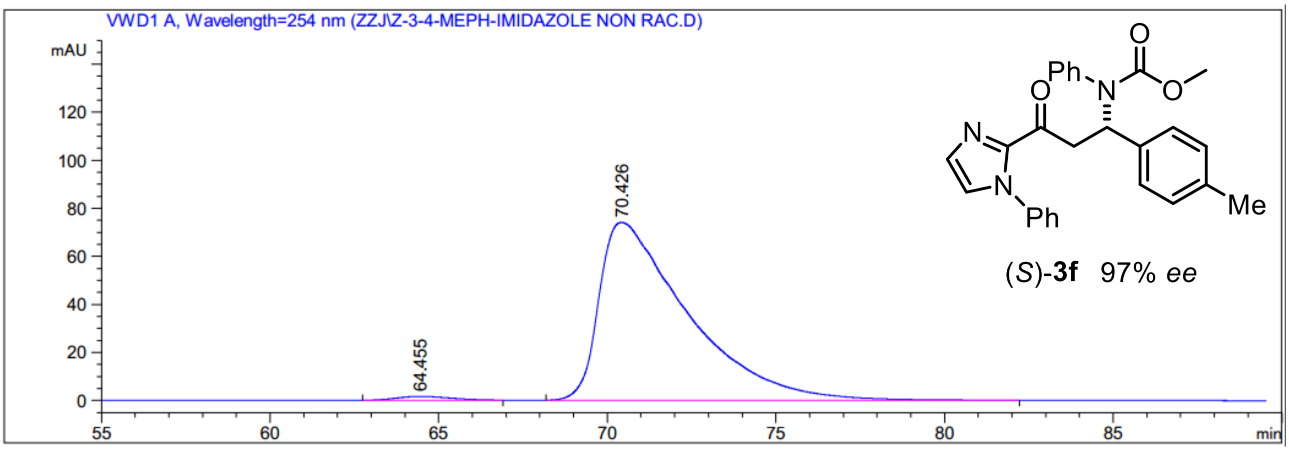

#	[min]		[min]	[mAU*s]	[mAU]	%
1	64.455	MM R	1.9024	191.91647	1.68134	1.3879
2	70.426	MM R	2.3648	1.36361e4	74.18615	98.6121

Figure S6. HPLC trace for the racemic reference *rac*-**3f**, and non-racemic product (*S*)-**3f** generated from the asymmetric reaction catalyzed by Δ-**RhO**.

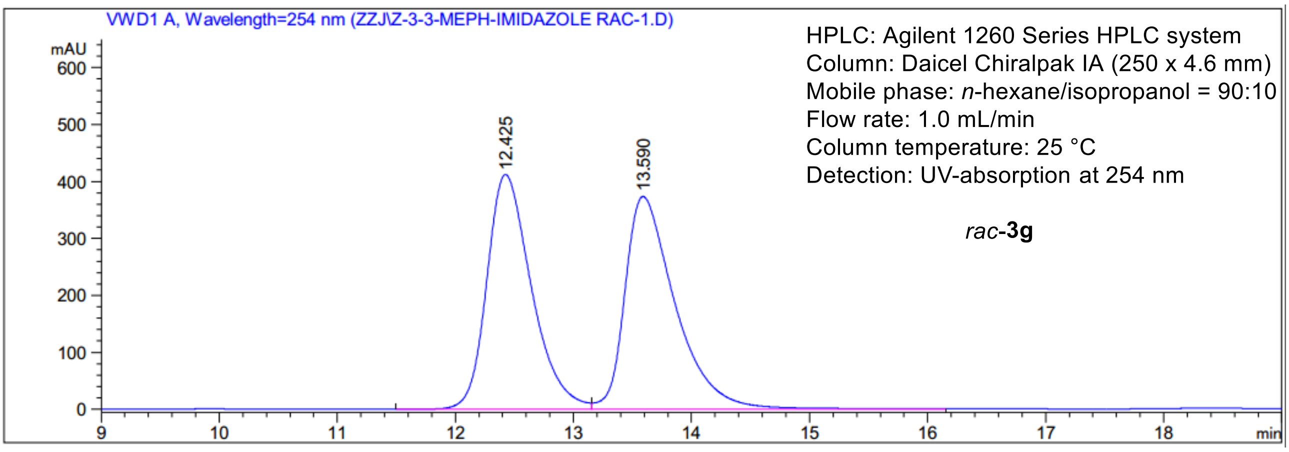

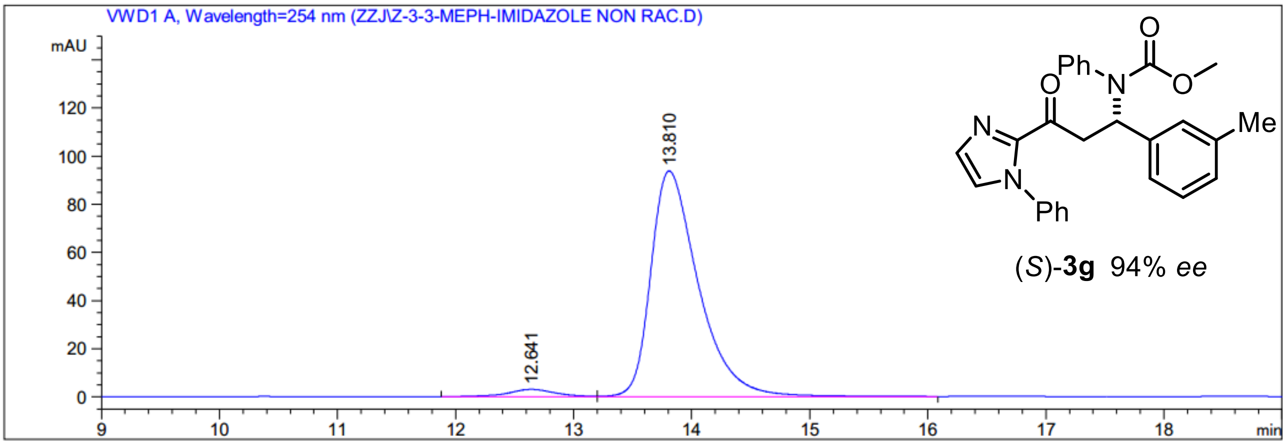

#	[min]		[min]	[mAU*s]	[mAU]	%
1	12.641	BV	0.4165	83.53828	3.00303	3.0753
2	13.810	VV	0.4236	2632.85400	93.75816	96.9247

Figure S7. HPLC trace for the racemic reference *rac*-**3g**, and non-racemic product (*S*)-**3g** generated from the asymmetric reaction catalyzed by Δ-**RhO**.

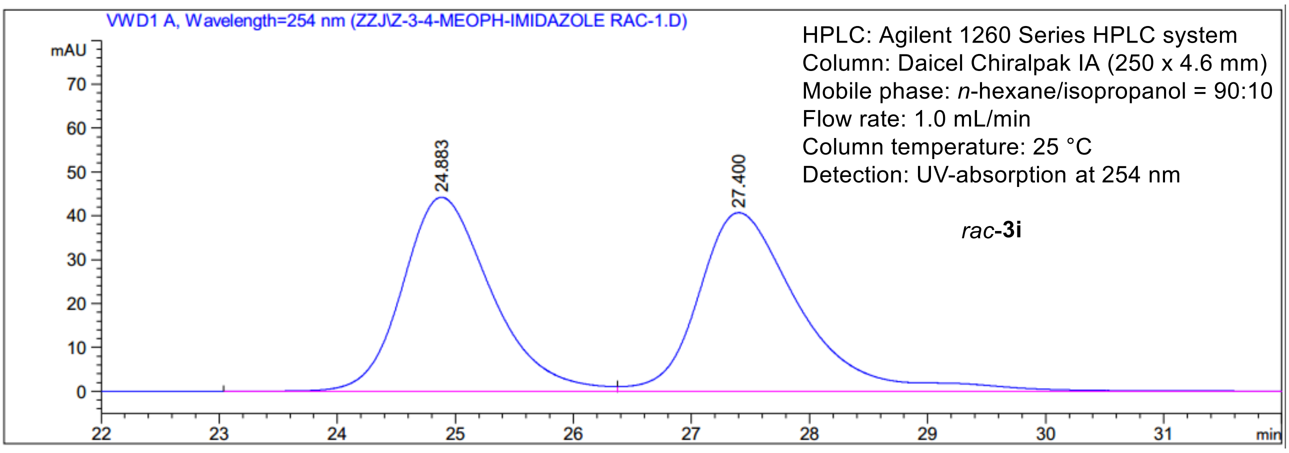

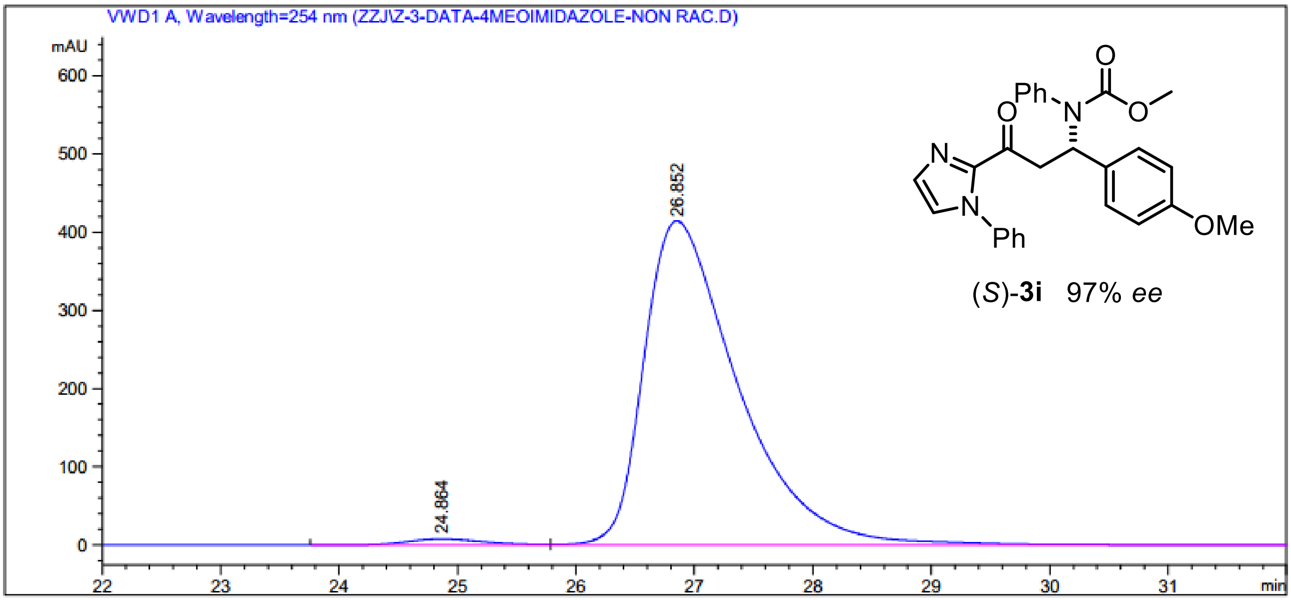

#	[min]		[min]	[mAU*s]	[mAU]	%
1	24.864	BV	0.7224	364.75046	7.78741	1.5637
2	26.852	VB	0.8346	2.29609e4	414.29520	98.4363

Figure S8. HPLC trace for the racemic reference *rac*-**3i**, and non-racemic product (*S*)-**3i** generated from the asymmetric reaction catalyzed by Δ-**RhO**.

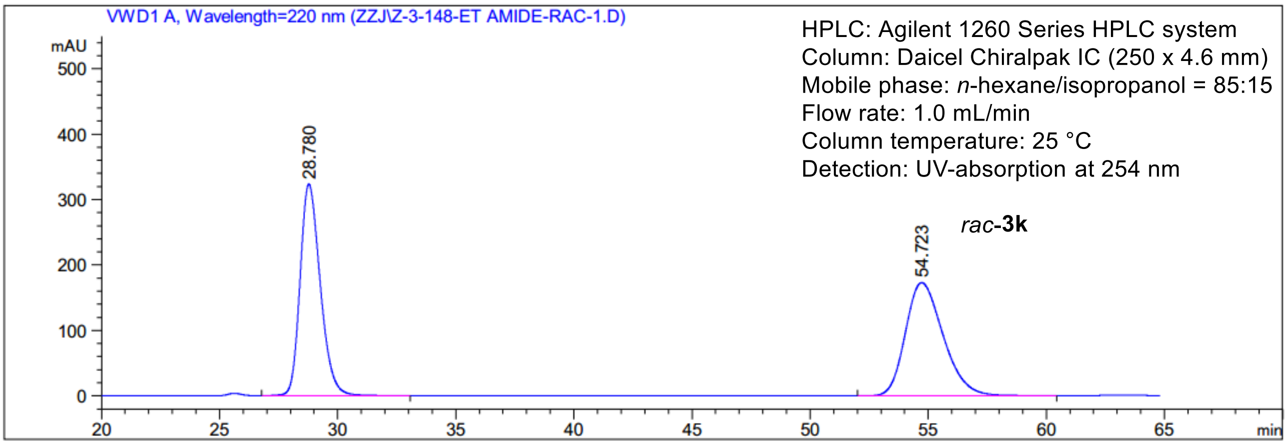

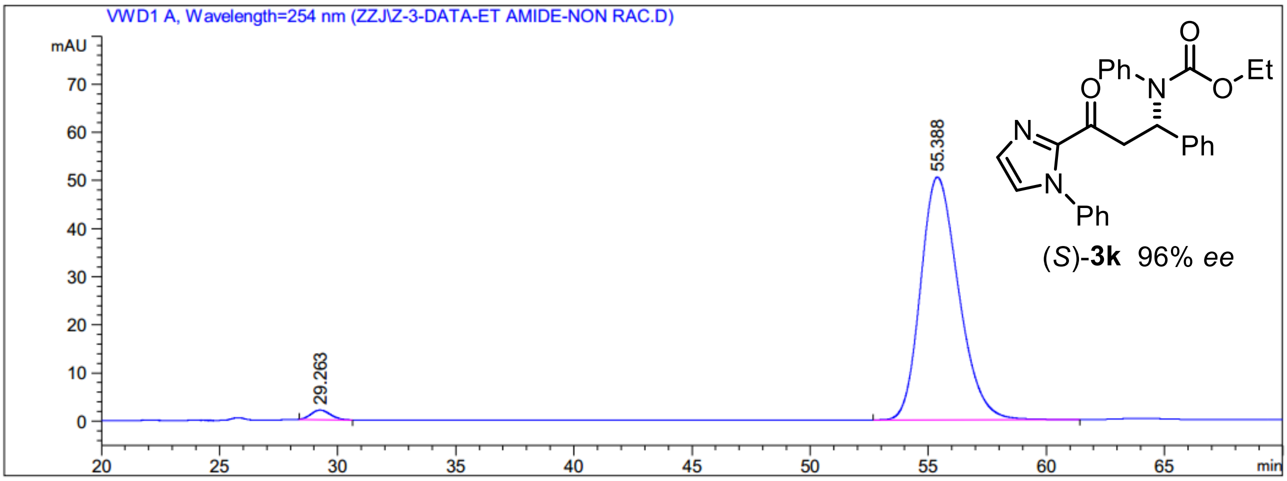

#	[min]		[min]	[mAU*s]	[mAU]	%
1	29.263	MP R	0.9406	112.29086	1.98972	1.9516
2	55.388	BB	1.7212	5641.60449	50.42730	98.0484

Figure S9. HPLC trace for the racemic reference *rac*-**3k**, and non-racemic product (*S*)-**3k** generated from the asymmetric reaction catalyzed by Δ-**RhO**.

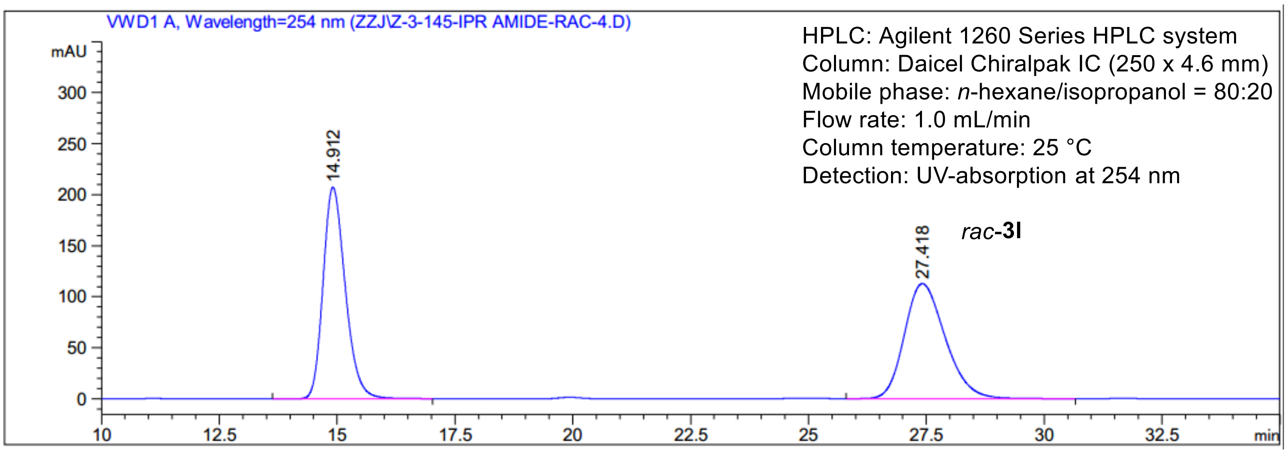

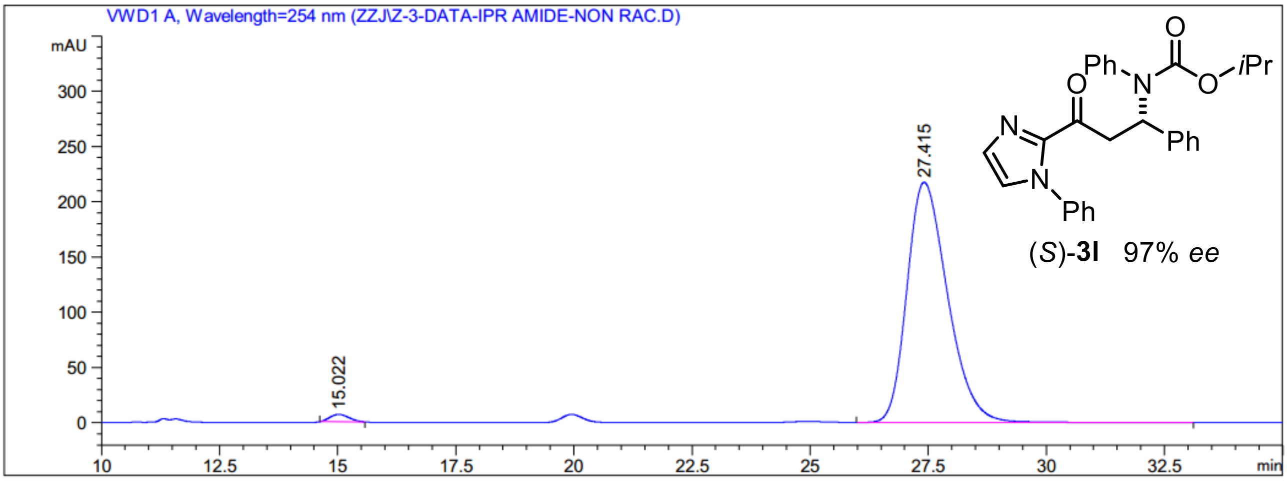

#	[min]		[min]	[mAU*s]	[mAU]	%
1	15.022	MP R	0.4718	190.91461	6.74476	1.4436
2	27.415	VB	0.9276	1.30342e4	217.36940	98.5564

Figure S10. HPLC trace for the racemic reference *rac*-**3l** and non-racemic product (*S*)-**3l** generated from the asymmetric reaction catalyzed by Δ-**RhO**.

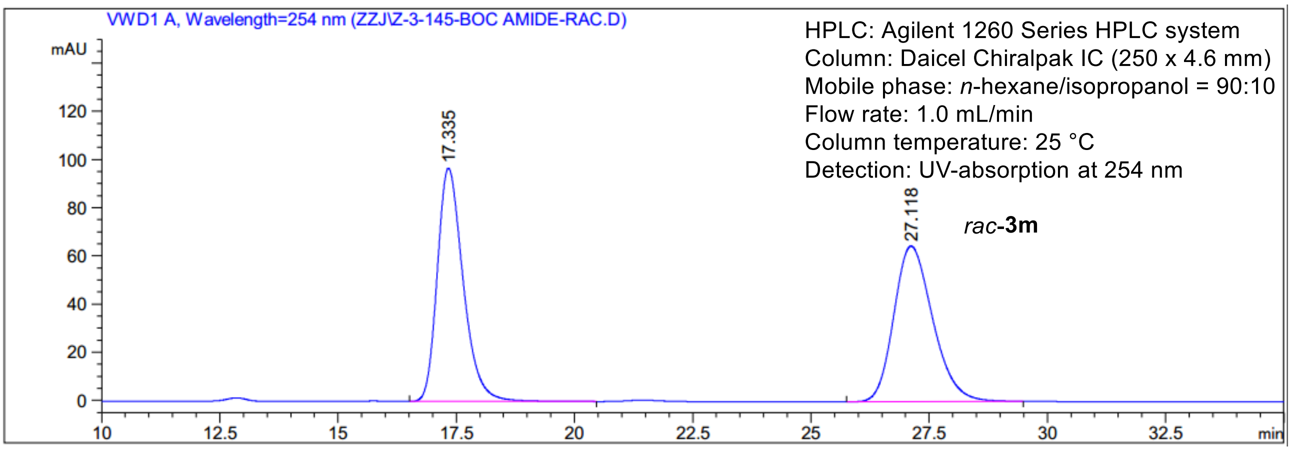

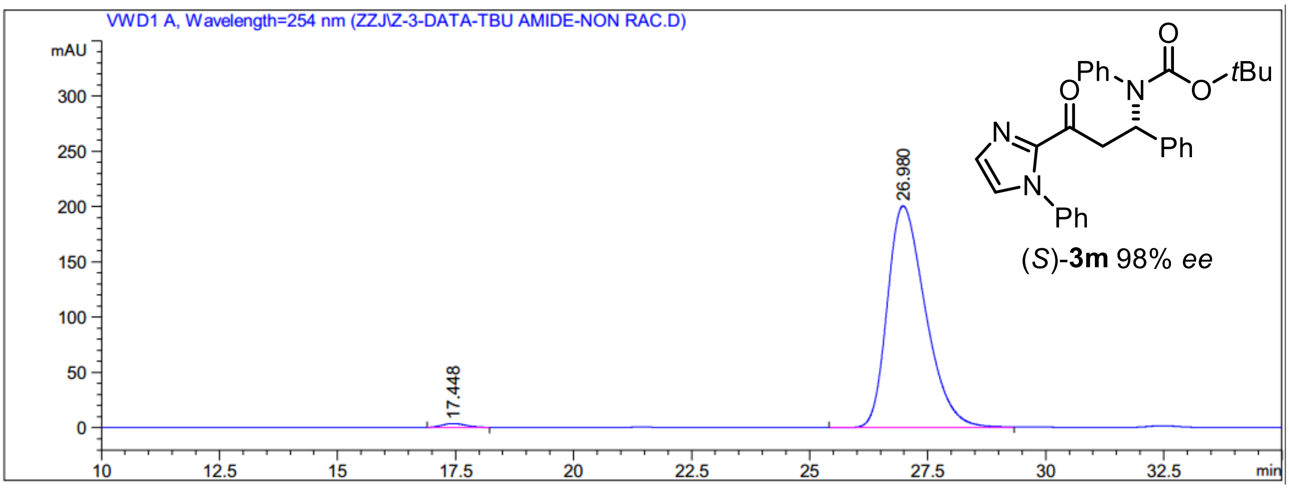

#	[min]		[min]	[mAU*s]	[mAU]	%
1	17.448	MP R	0.4323	129.72385	3.67492	1.1374
2	26.980	BV	0.8643	1.12756e4	200.44366	98.8626

Figure S11. HPLC trace for the racemic reference *rac*-**3m** and non-racemic product (*S*)-**3m** generated from the asymmetric reaction catalyzed by Δ-**RhO**.

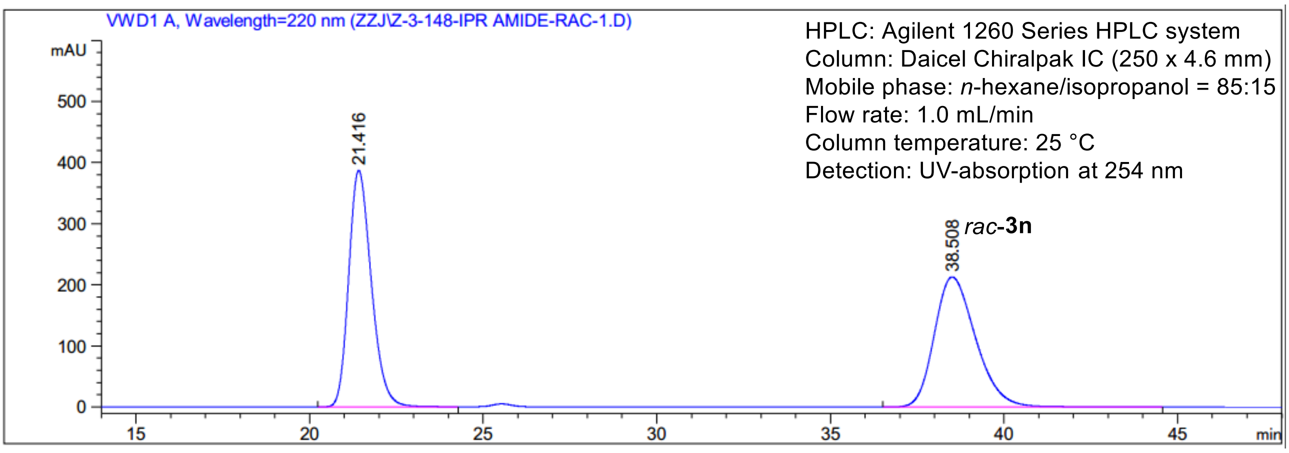

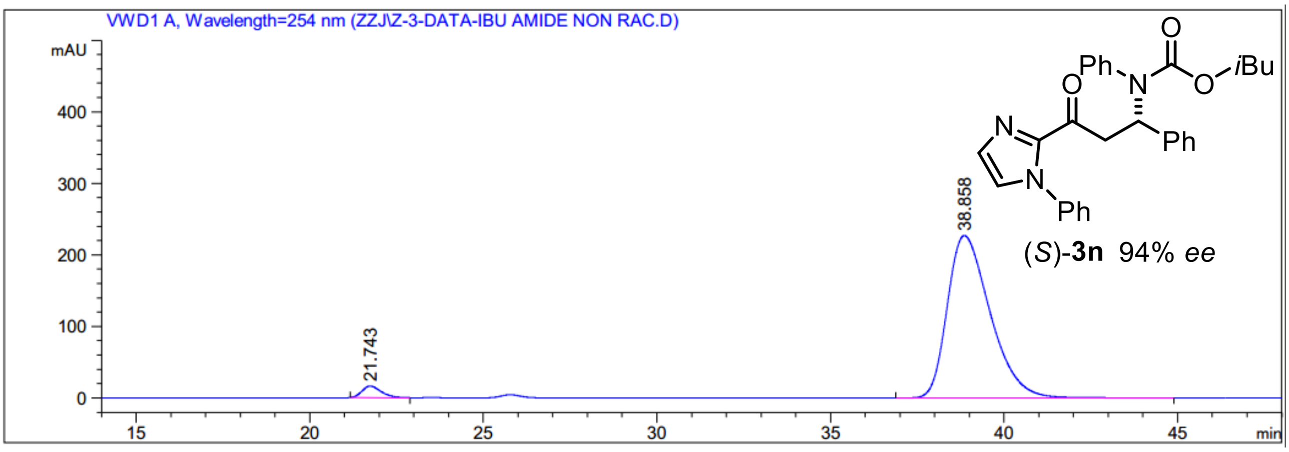

#	[min]		[min]	[mAU*s]	[mAU]	%
1	21.743	MM R	0.5607	641.16498	16.39578	3.1921
2	38.858	BB	1.3234	1.94448e4	227.00038	96.8079

Figure S12. HPLC trace for the racemic reference *rac*-**3n** and non-racemic product (*S*)-**3n** generated from the asymmetric reaction catalyzed by Δ-**RhO**.

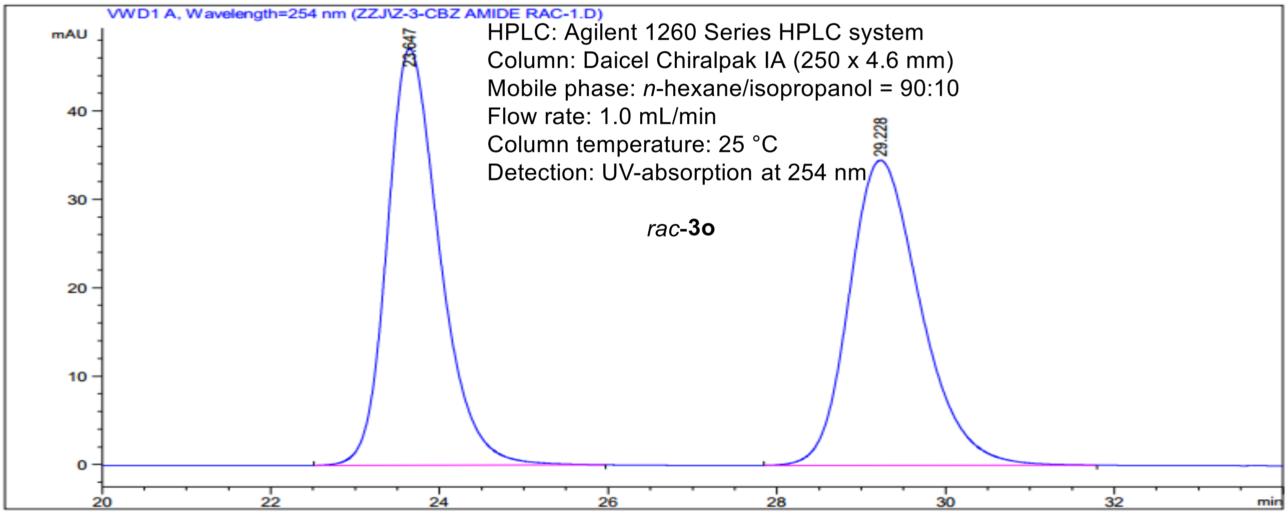

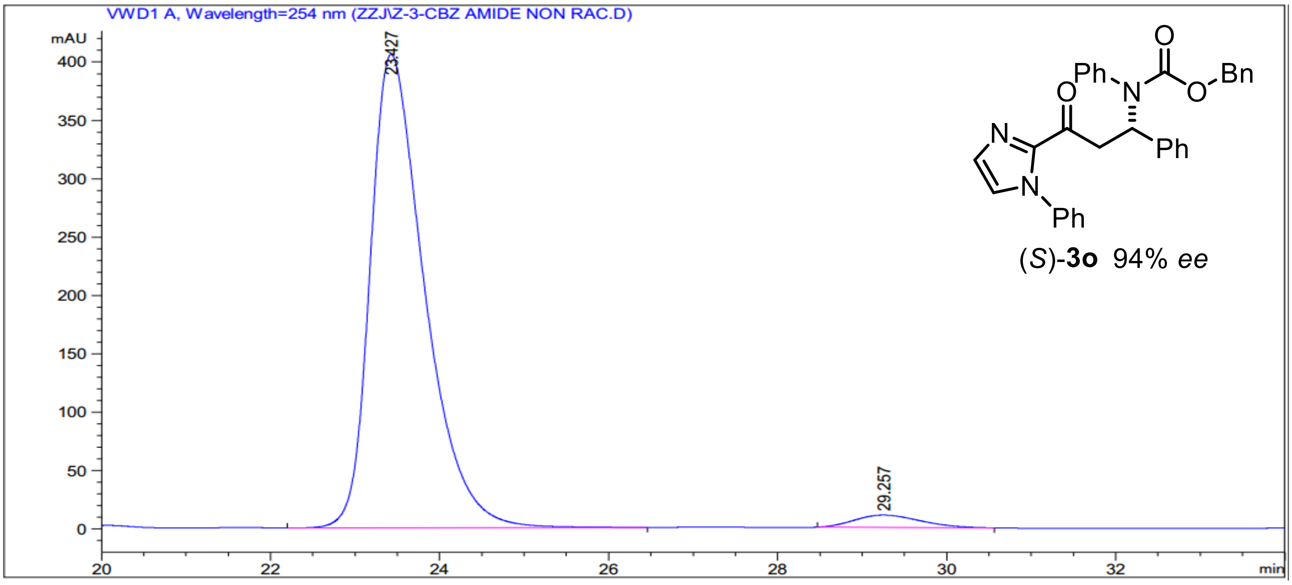

#	[min]		[min]	[mAU*s]	[mAU]	%
1	23.427	BB	0.6753	1.80945e4	405.75488	96.8778
2	29.257	MM R	0.8251	583.15161	10.63230	3.1222

Figure S13. HPLC trace for the racemic reference *rac*-**3o** and non-racemic product (*S*)-**3o** generated from the asymmetric reaction catalyzed by Δ-**RhO**.

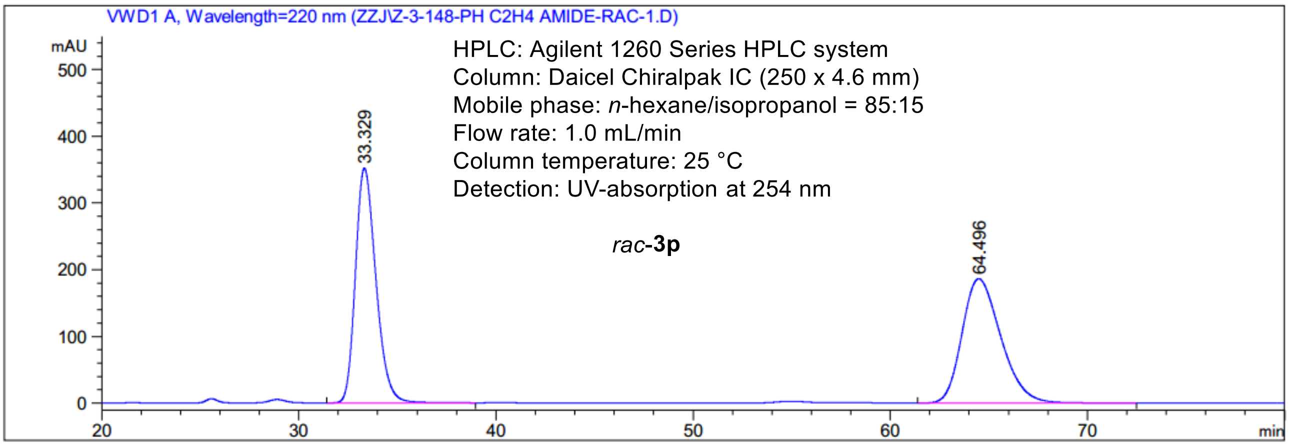

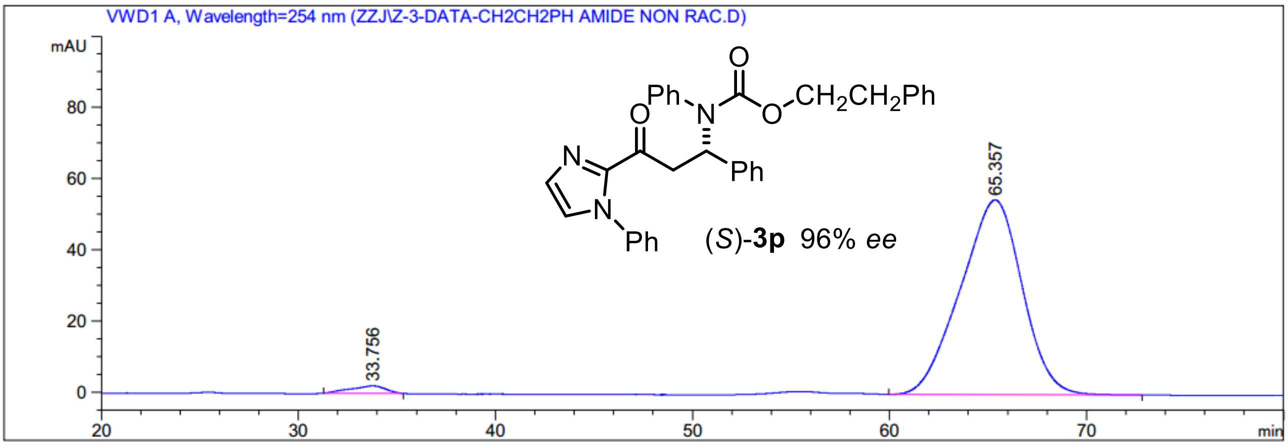

#	[min]		[min]	[mAU*s]	[mAU]	%
1	33.756	MM R	2.0811	264.30676	2.11670	2.1680
2	65.357	BB	3.0981	1.19270e4	54.73856	97.8320

Figure S14. HPLC trace for the racemic reference *rac*-**3p** and non-racemic product (*S*)-**3p** generated from the asymmetric reaction catalyzed by Δ-**RhO**.

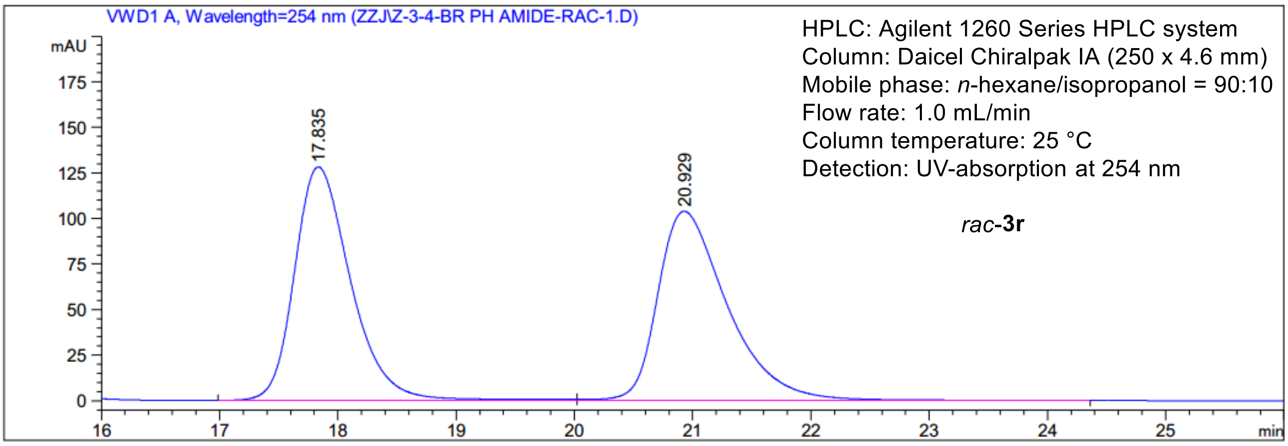

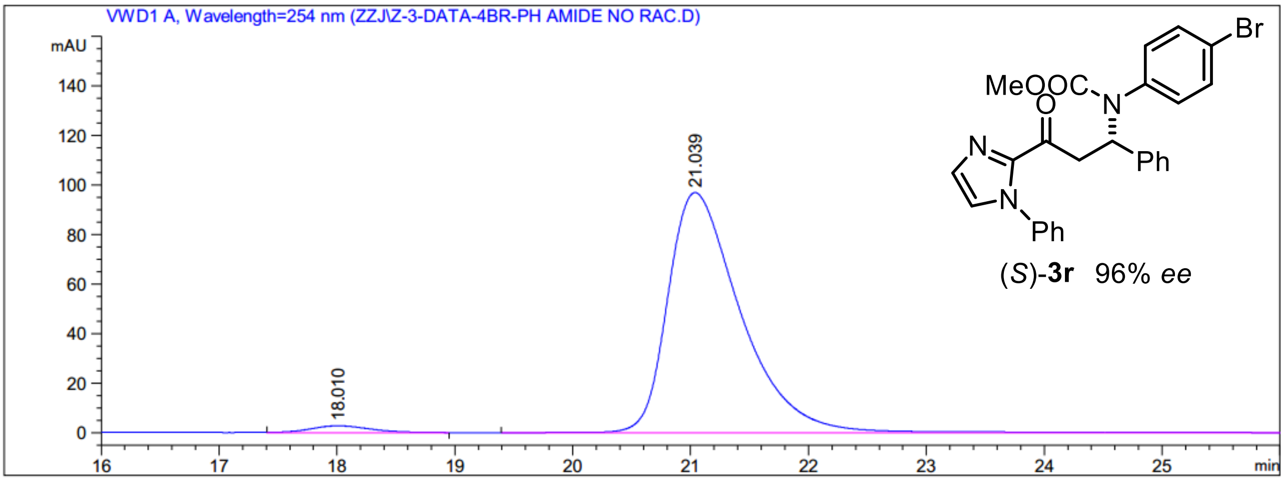

#	[min]		[min]	[mAU*s]	[mAU]	%
1	18.010	MP R	0.4254	95.02179	2.72084	2.2198
2	21.039	BB	0.6523	4185.65186	96.82748	97.7802

Figure S15. HPLC trace for the racemic reference *rac*-**3r** and non-racemic product (*S*)-**3r** generated from the asymmetric reaction catalyzed by Δ-**RhO**.

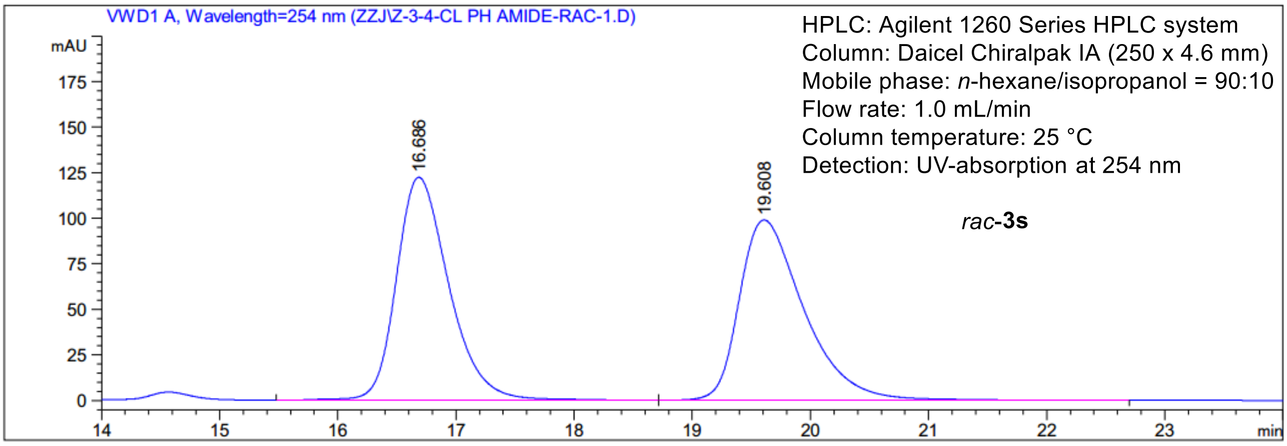

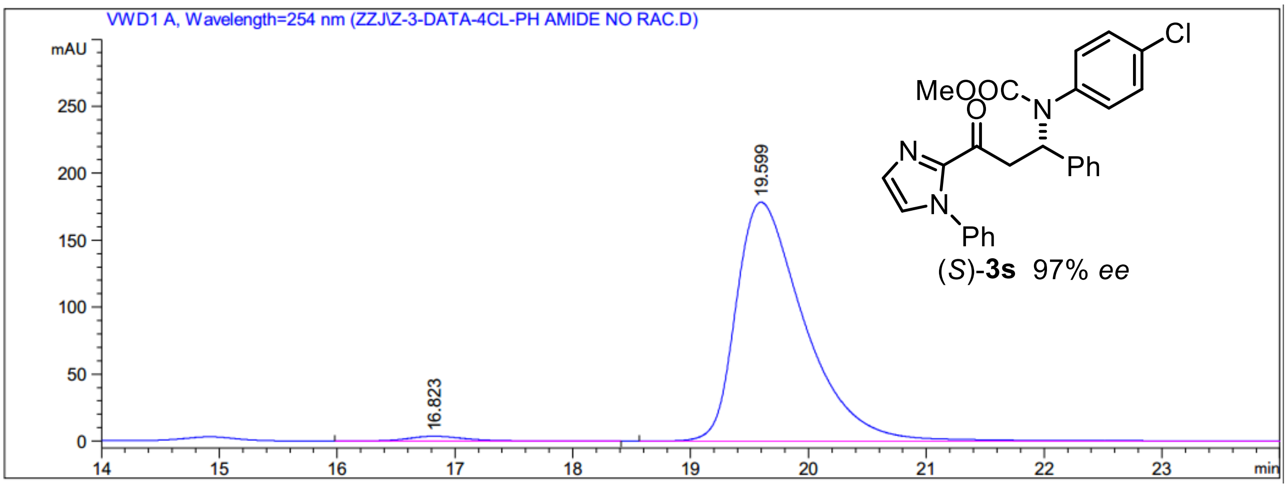

#	[min]		[min]	[mAU*s]	[mAU]	%
1	16.823	BB	0.5123	120.90552	3.59540	1.6497
2	19.599	BBA	0.6116	7208.20703	178.33112	98.3503

Figure S16. HPLC trace for the racemic reference *rac*-**3s** and non-racemic product (*S*)-**3s** generated from the asymmetric reaction catalyzed by Δ-**RhO**.

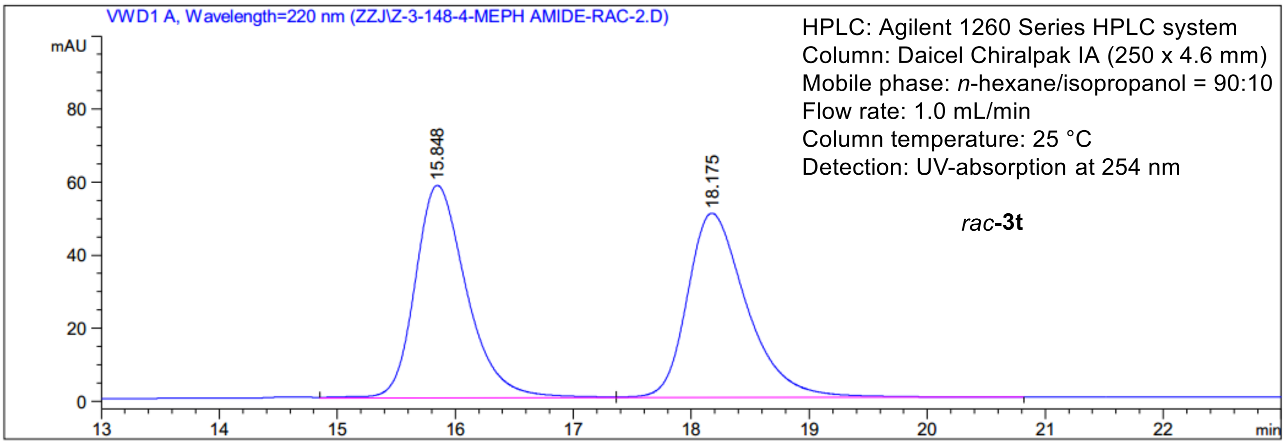

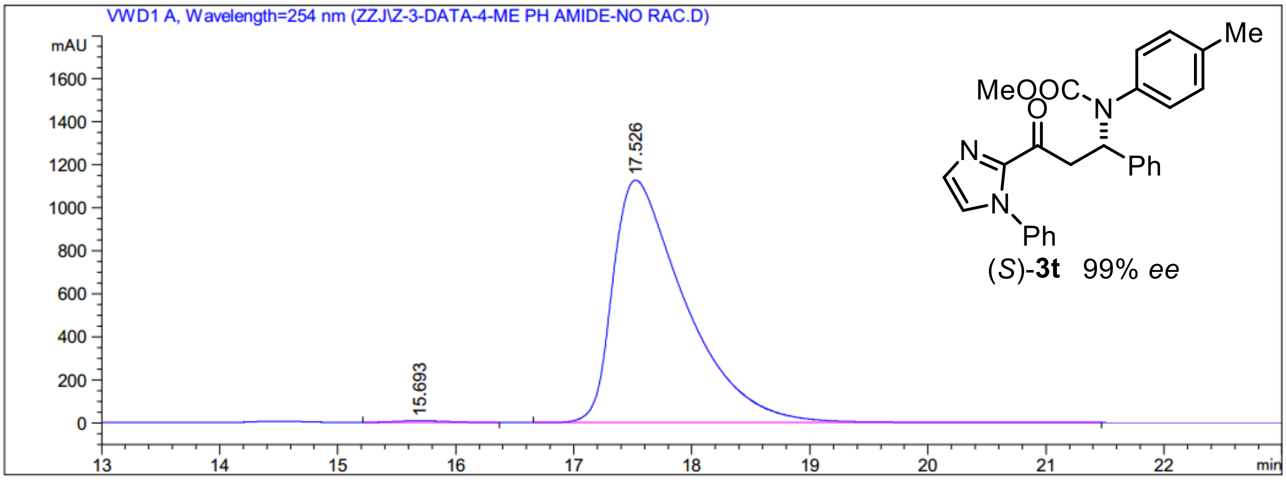

#	[min]		[min]	[mAU*s]	[mAU]	%
1	15.693	MM R	0.5186	263.17435	8.14199	0.5425
2	17.526	VV	0.6309	4.82459e4	1128.27869	99.4575

Figure S17. HPLC trace for the racemic reference *rac*-**3t** and non-racemic product (*S*)-**3t** generated from the asymmetric reaction catalyzed by Δ-**RhO**.

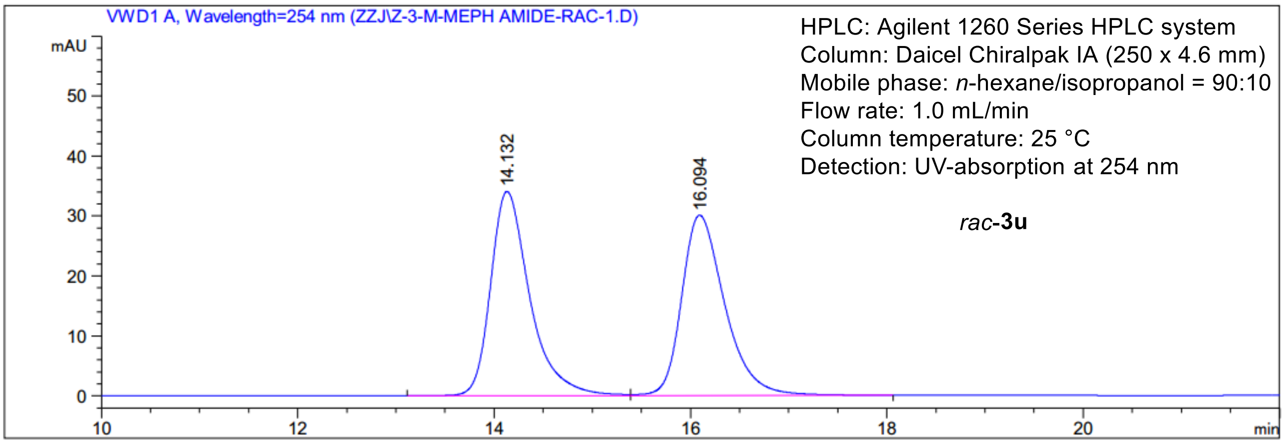

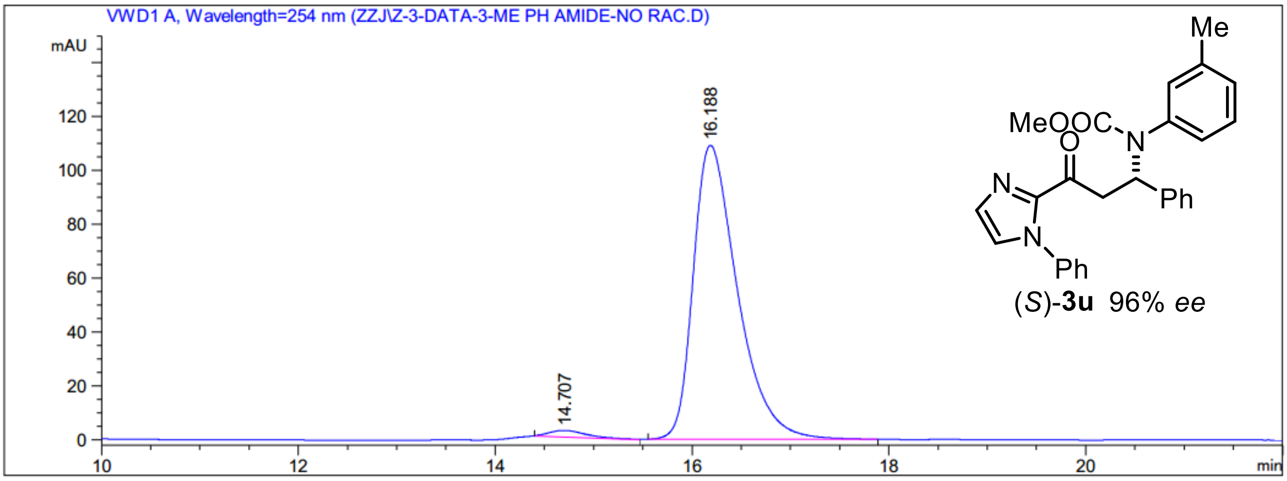

#	[min]		[min]	[mAU*s]	[mAU]	%
1	14.707	PM R	0.4475	66.59849	2.48050	1.9167
2	16.188	MM R	0.3638	3408.03052	109.24108	98.0833

Figure S18. HPLC trace for the racemic reference *rac*-**3u** and non-racemic product (*S*)-**3u** generated from the asymmetric reaction catalyzed by Δ-**RhO**.

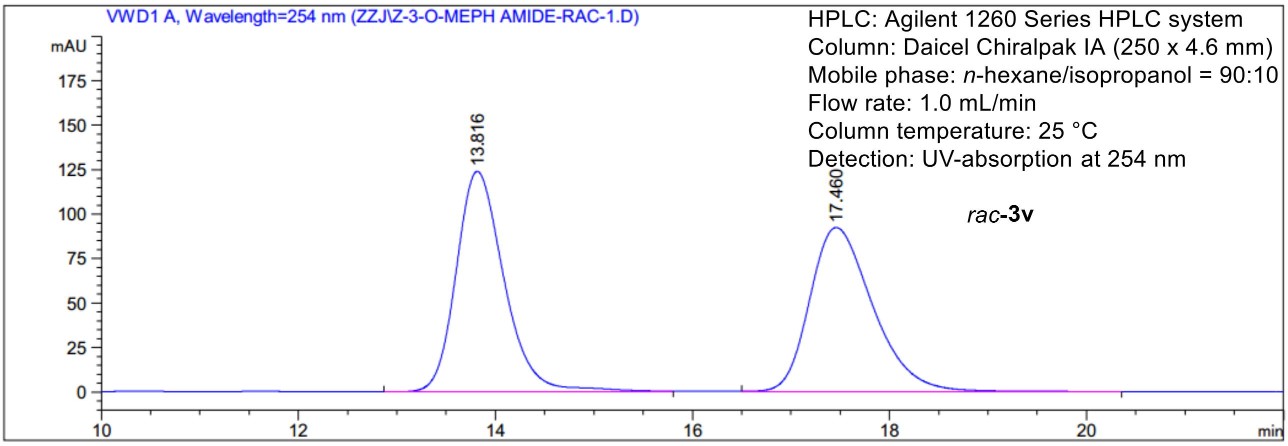

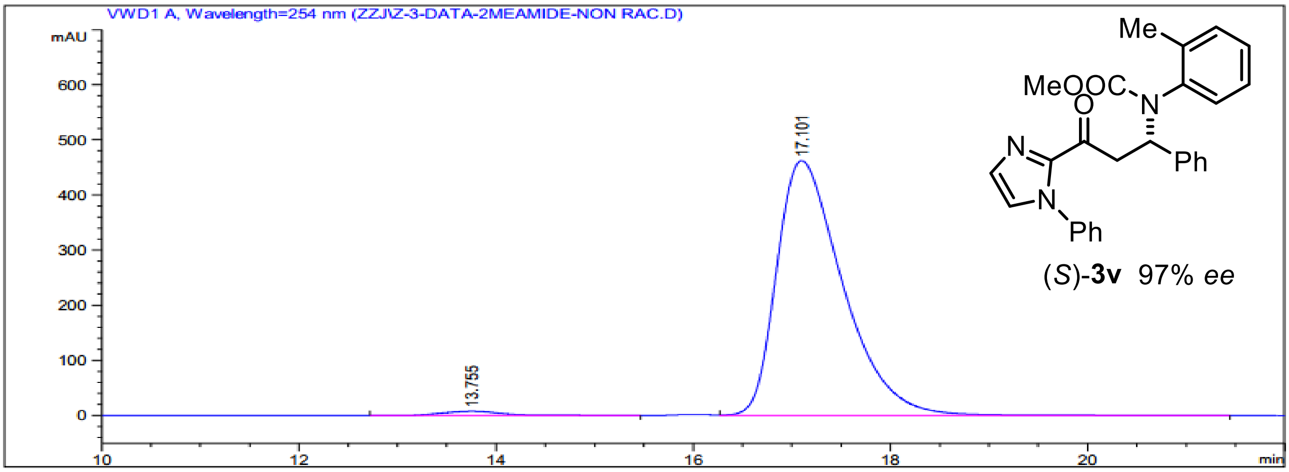

#	[min]		[min]	[mAU*s]	[mAU]	%
1	13.755	BV	0.7154	349.31885	7.61155	1.5881
2	17.101	VV	0.7146	2.16471e4	462.06723	98.4119

Figure S19. HPLC trace for the racemic reference *rac*-**3v** and non-racemic product (*S*)-**3v** generated from the asymmetric reaction catalyzed by Δ-**RhO**.

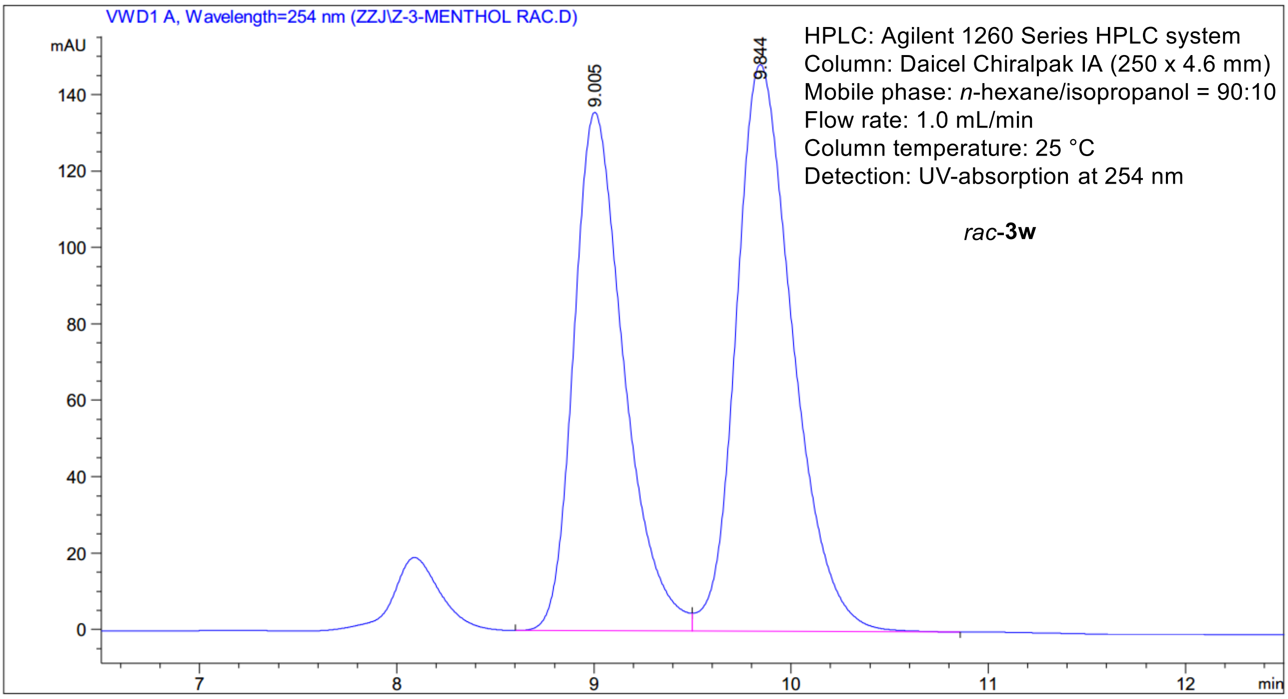

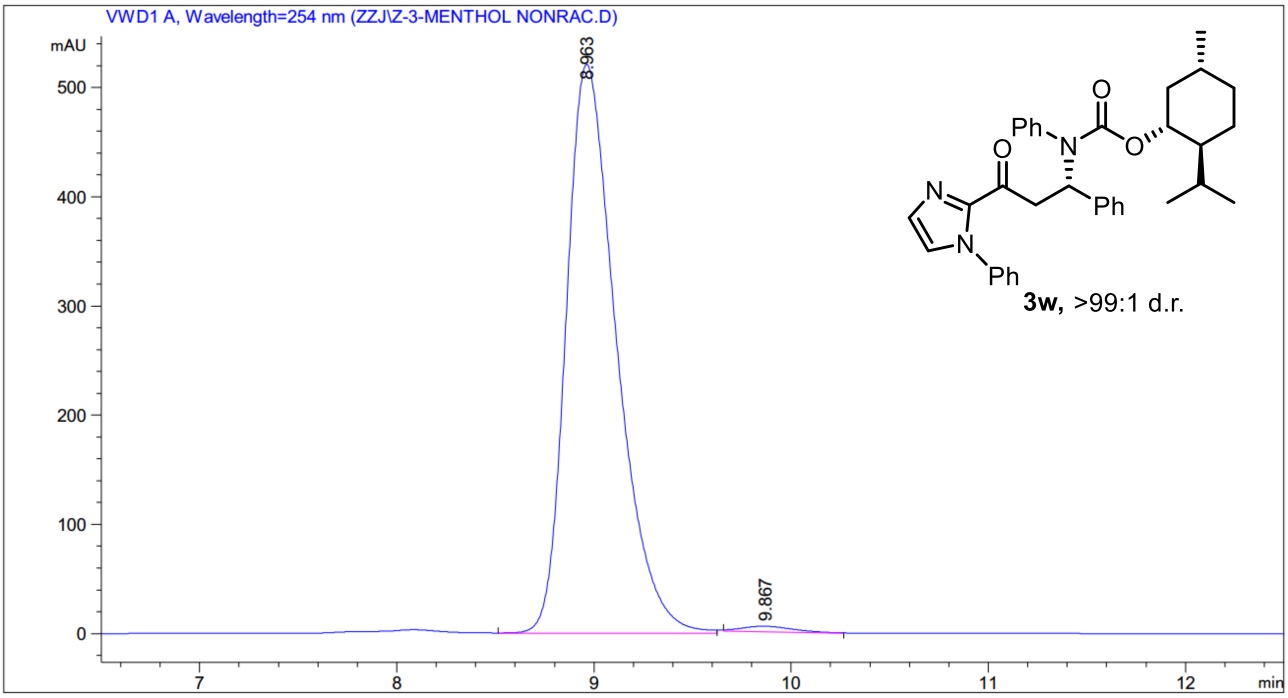

#	[min]		[min]	[mAU*s]	[mAU]	%
1	8.963	VV	0.2706	9225.29395	520.97357	99.0209
2	9.867	MM R	0.3036	91.21444	5.00658	0.9791

Figure S20. HPLC trace for the racemic reference *rac*-**3w** and non-racemic product **3w** generated from the asymmetric reaction catalyzed by Δ-**RhO**.

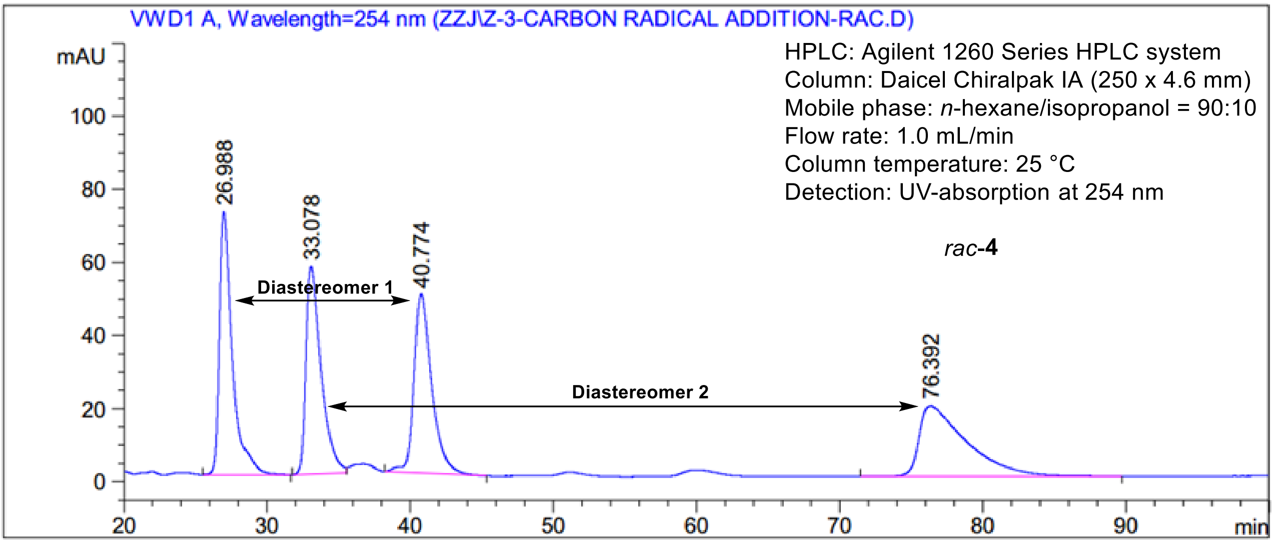

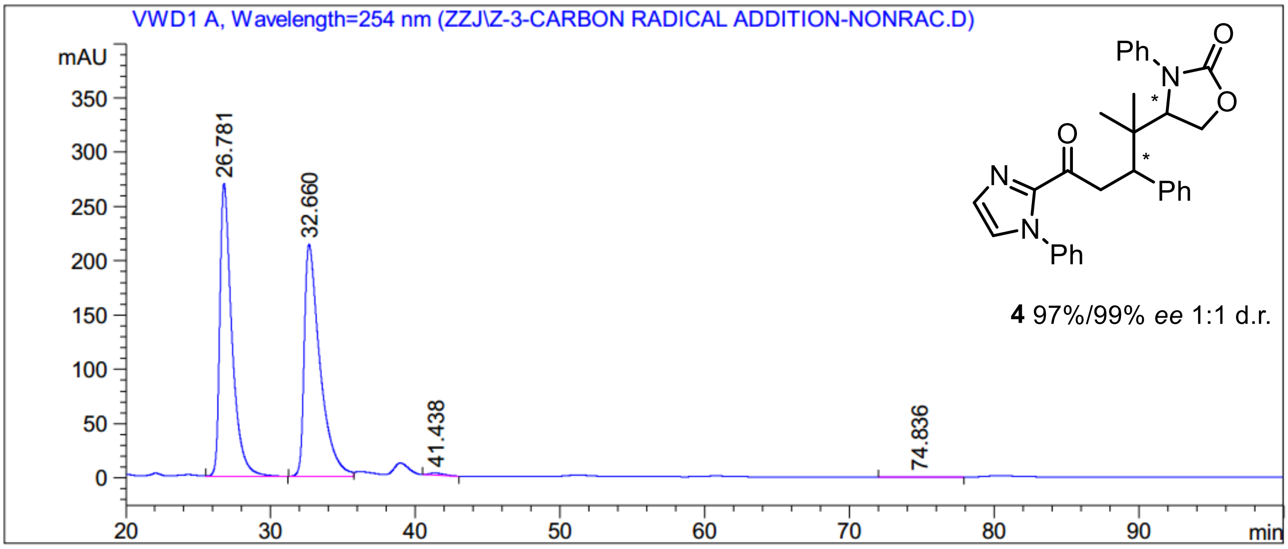

#	[min]		[min]	[mAU*s]	[mAU]	%
1	26.781	VB	0.8646	1.56328e4	269.55789	48.9129
2	32.660	BV	1.0984	1.61515e4	213.55939	50.5359
3	41.438	MP R	1.1555	130.44128	1.88150	0.4081
4	74.836	BV	2.3434	45.73684	2.31654e-1	0.1431

Figure S21. HPLC trace for the racemic reference *rac*-**4** and non-racemic product **4** generated from the asymmetric reaction catalyzed by Δ-**RhO**.

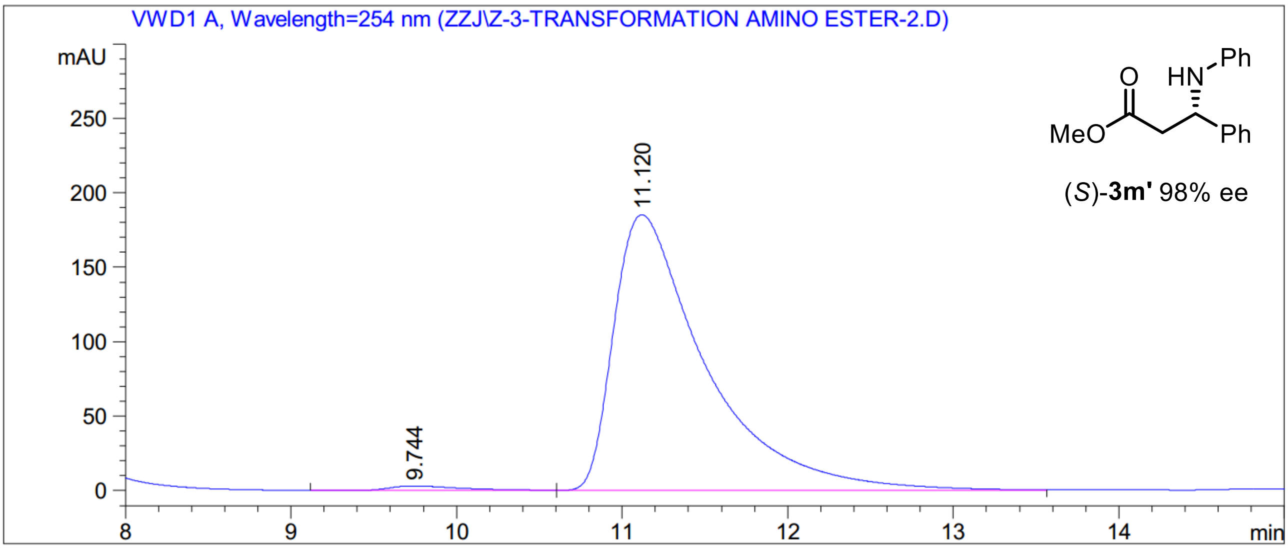

#	[min]		[min]	[mAU*s]	[mAU]	%
1	9.744	BB	0.4317	84.40687	2.89929	1.1682
2	11.120	BV	0.5625	7141.25586	184.95351	98.8318

Figure S22. HPLC trace for the non-racemic product (*S*)-**3m'**. (Racemic reference see Ref. 8)

8. Photoreaction in the Absence of a Photoredox Mediator

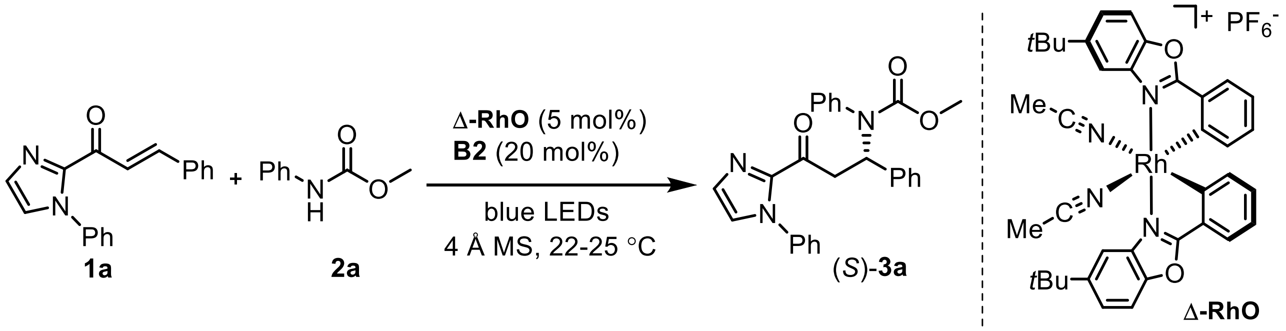

General procedure:

To a solution of α,β-unsaturated 2-acyl imidazole **1a** (32.90 mg, 0.12 mmol) in fresh distilled CH_2Cl_2 (1.0 mL, 0.1 M) was added rhodium (III) complex Δ-**RhO** (4.15 mg, 0.005 mmol, 5 mol%), **B2** (9.83 mg, 20 mol%), 4 Å MS (10.0 mg, 100 mg/mmol) in a 10 mL Schlenk tube and the resulting solution was stirred at room temperature for 15 min. Then, carbamate **2a** (15.10 mg, 0.10 mmol) was added and the solution was bubbled for 15 min with argon. The reaction was stirred in a thermostatic cabinet (the actual temperature was about 22-25 °C) under blue LEDs (24 W + 36 W) irradiation (the Schlenk tube was about 3 cm to the light source). After 18 h, **3a** was afforded in 39% yield (analyzed by crude ^{1}H NMR).

Color change experiment:

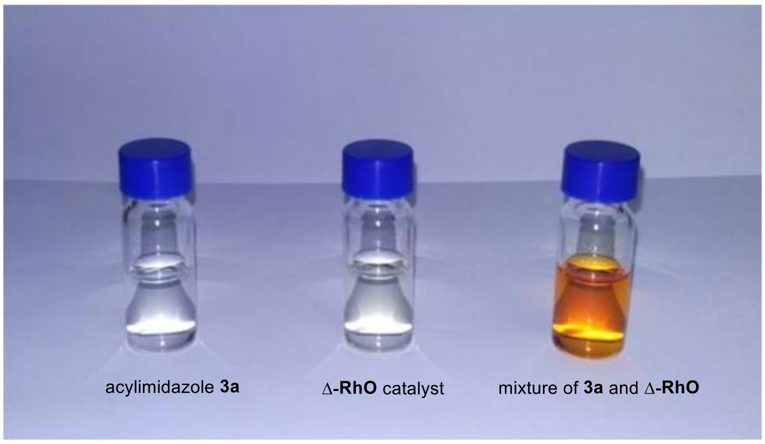

Figure S23. Result of color change experiment. From left to right : 0.1mmol **3a** (in 1 mL CH_2Cl_2); 0.005mmol Δ-**RhO** (in 1 mL CH_2Cl_2); 0.1mmol **3a** coordinated with 0.005mmol Δ-**RhO** (in 1 mL CH_2Cl_2); The color change result somehow indicated the substrate-coordinated rhodium (III) complex become a potential photooxidant in the C-N radical coupling reaction.

Proposed mechanism (in the absence of **PC1**):

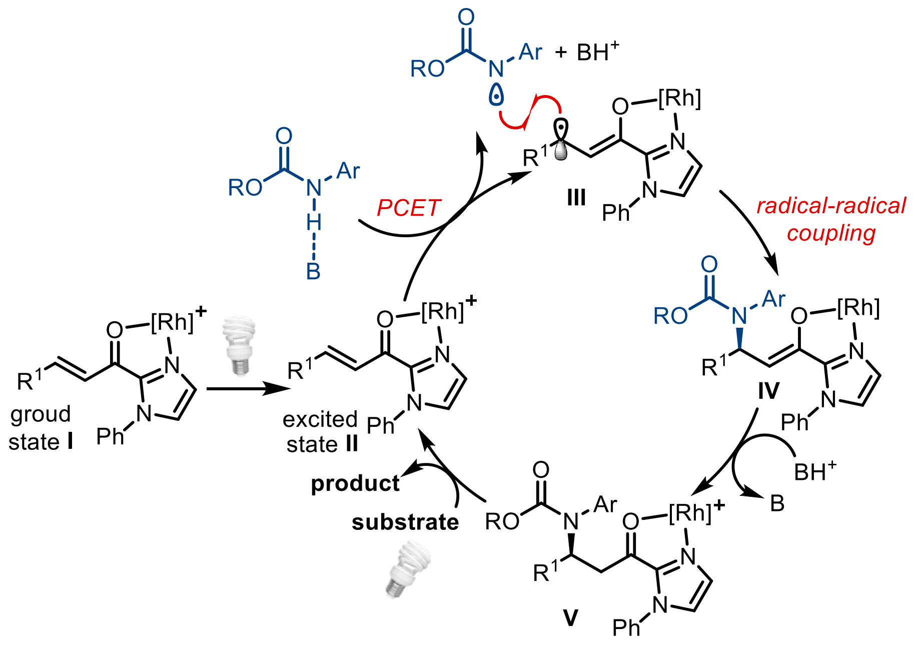

Figure S24. Proposed mechanism (in the absence of **PC1**).

9. References

1. C. Wang, L.-A. Chen, H. Huo, X. Shen, K. Harms, L. Gong and E. Meggers, *Chem. Sci.*, 2015, **5**, 1094-1100.

2. J. Ma, X. Shen, K. Harms and E. Meggers, *Dalton Trans.*, 2016, **45**, 8320-8323.

3. H. Huo, C. Fu, K. Harms and E. Meggers, *J. Am. Chem. Soc.*, 2014, **136**, 2990-2993.

4. H. Huo, K. Harms and E. Meggers, *J. Am. Chem. Soc.*, 2016, **138**, 6936-6939.

5. N. Uhlig and C.-J. Li, *Chem.-Eur. J.*, 2014, **20**, 12066-12070.

6. G. J. Choi, Q. Zhu, D. C. Miller, C. J. Gu and R. R. Knowles, *Nature*, 2016, **539**, 268-271.

7. E. L. Tyson, E. P. Farney and T. P. Yoon, *Org. Lett.*, 2013, **14**, 1110-1113.

8. H.-J. Zheng, *Chem.-Eur. J.*, 2008, **14**, 9864-9867.

9. G. J. Choi and R. R. Knowles, *J. Am. Chem. Soc.*, 2015, **137**, 9226-9229.

10. ^{1}H & ^{13}C NMR Spectra

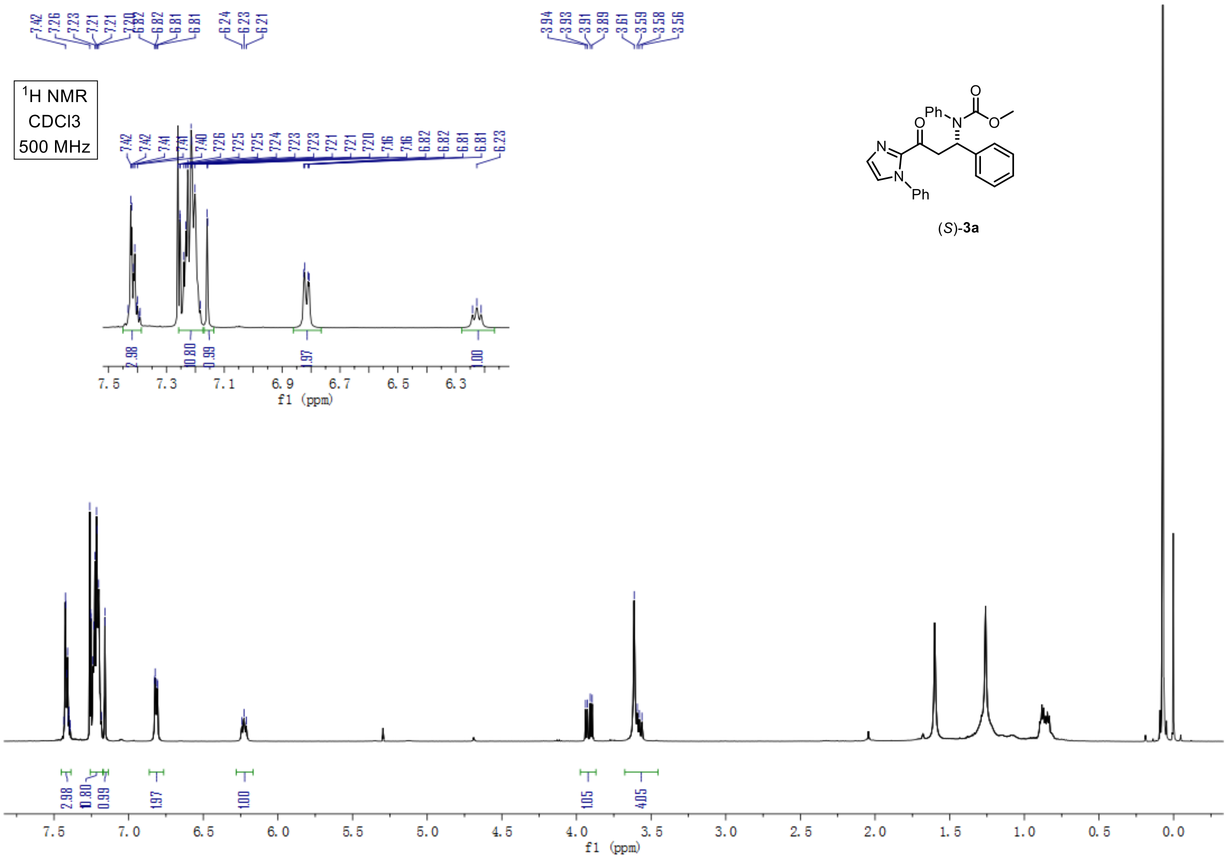

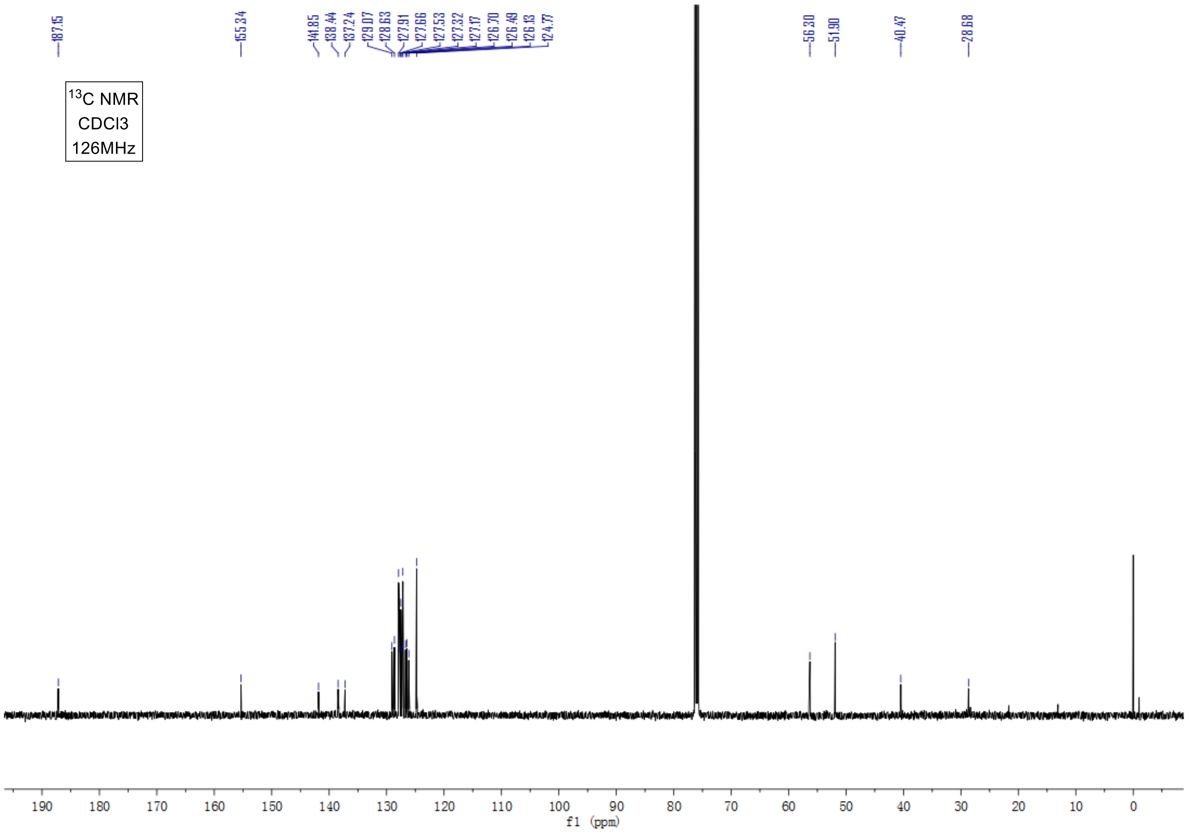

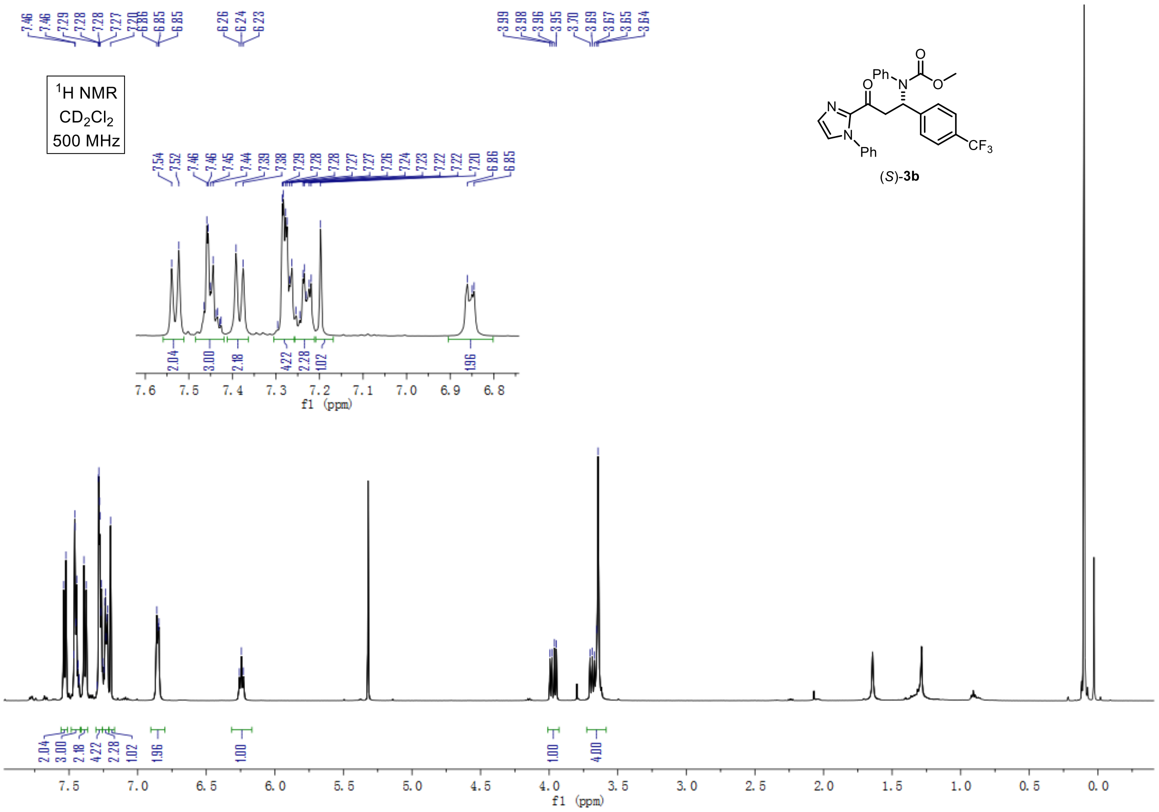

1H NMR
CD2Cl2
500 MHz
(S)-3b
f1 (ppm)

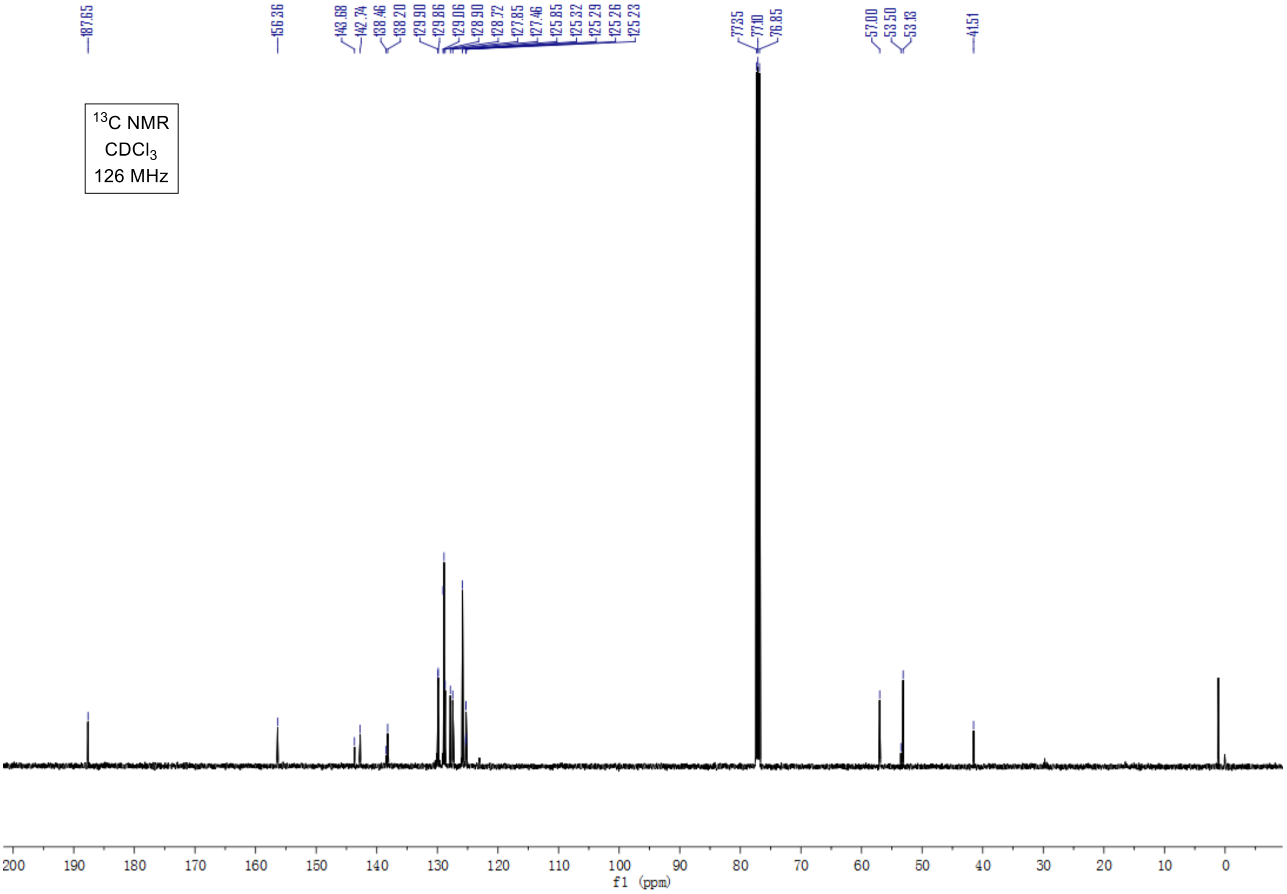

13C NMR
CDCl3
126 MHz
f1 (ppm)

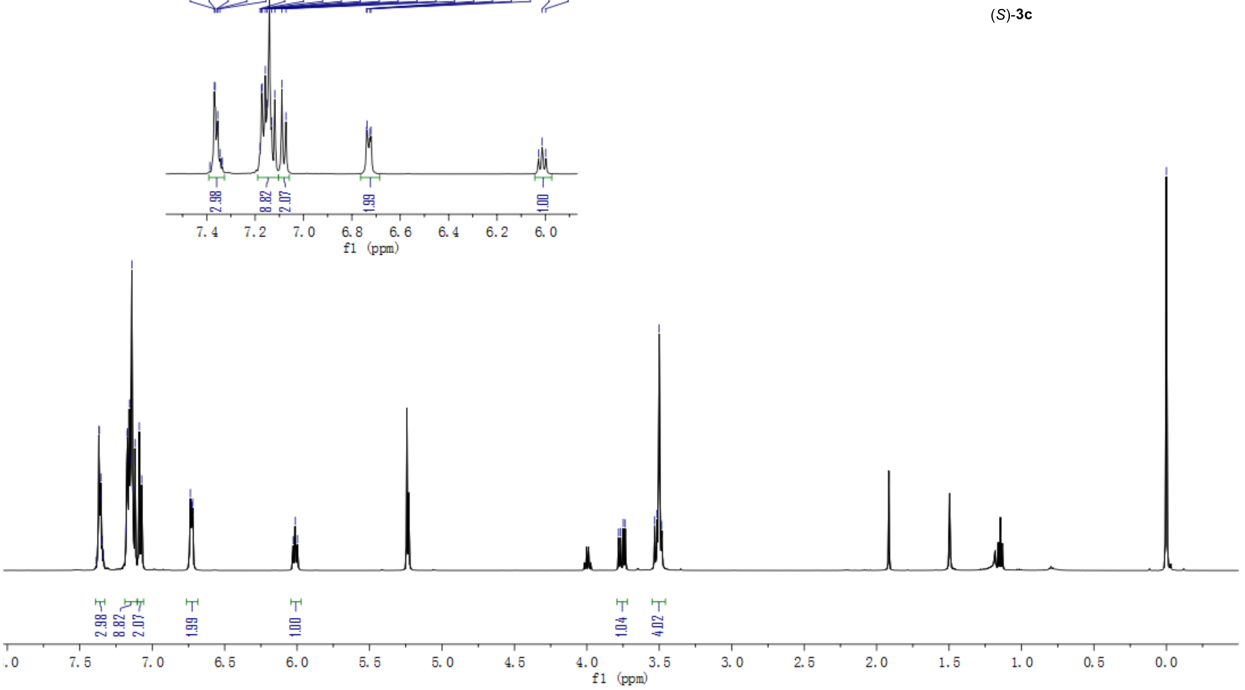

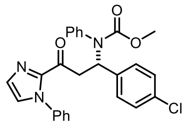

(*S*)-**3c**

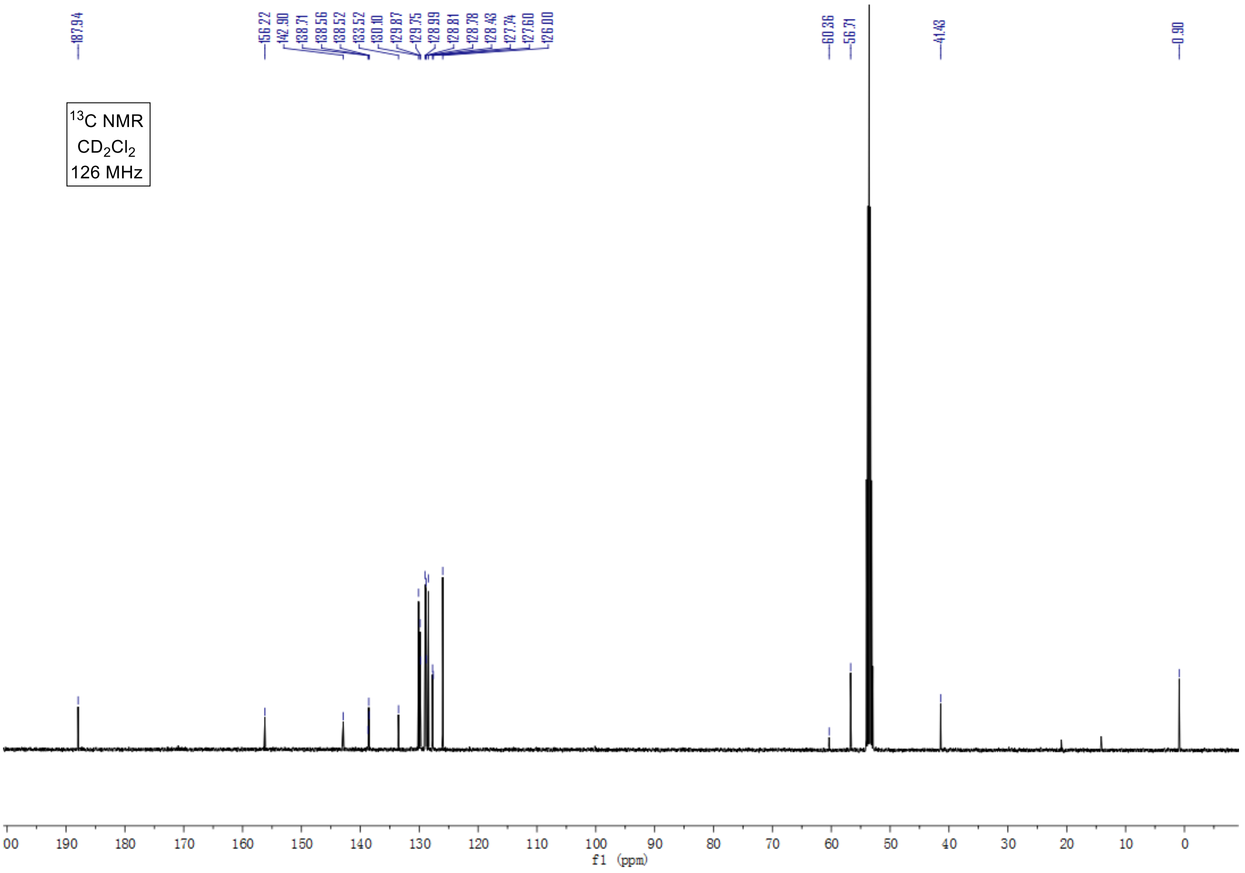

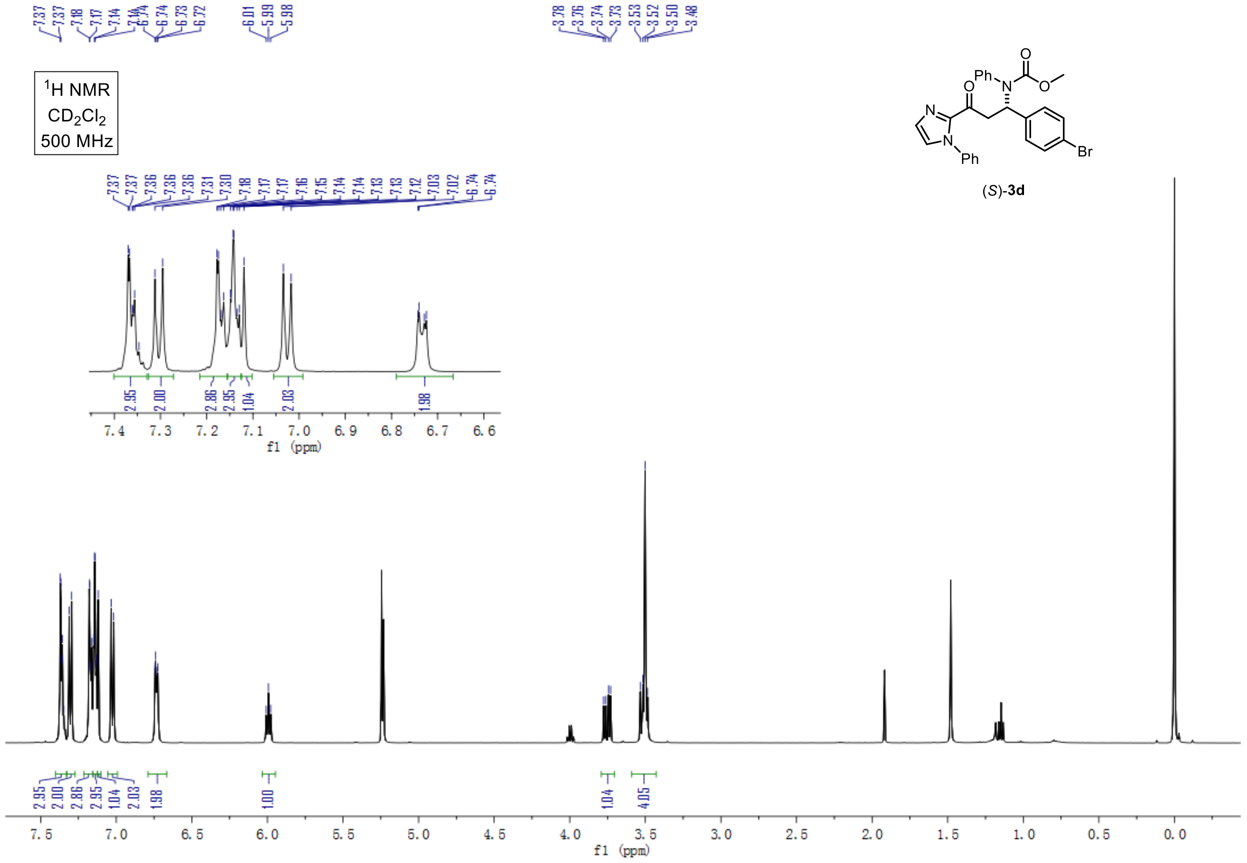

^{1}H NMR
CD_2Cl_2
500 MHz
f1 (ppm)
Ph
N
O
O
N
N
Ph
Br

(*S*)-**3d**

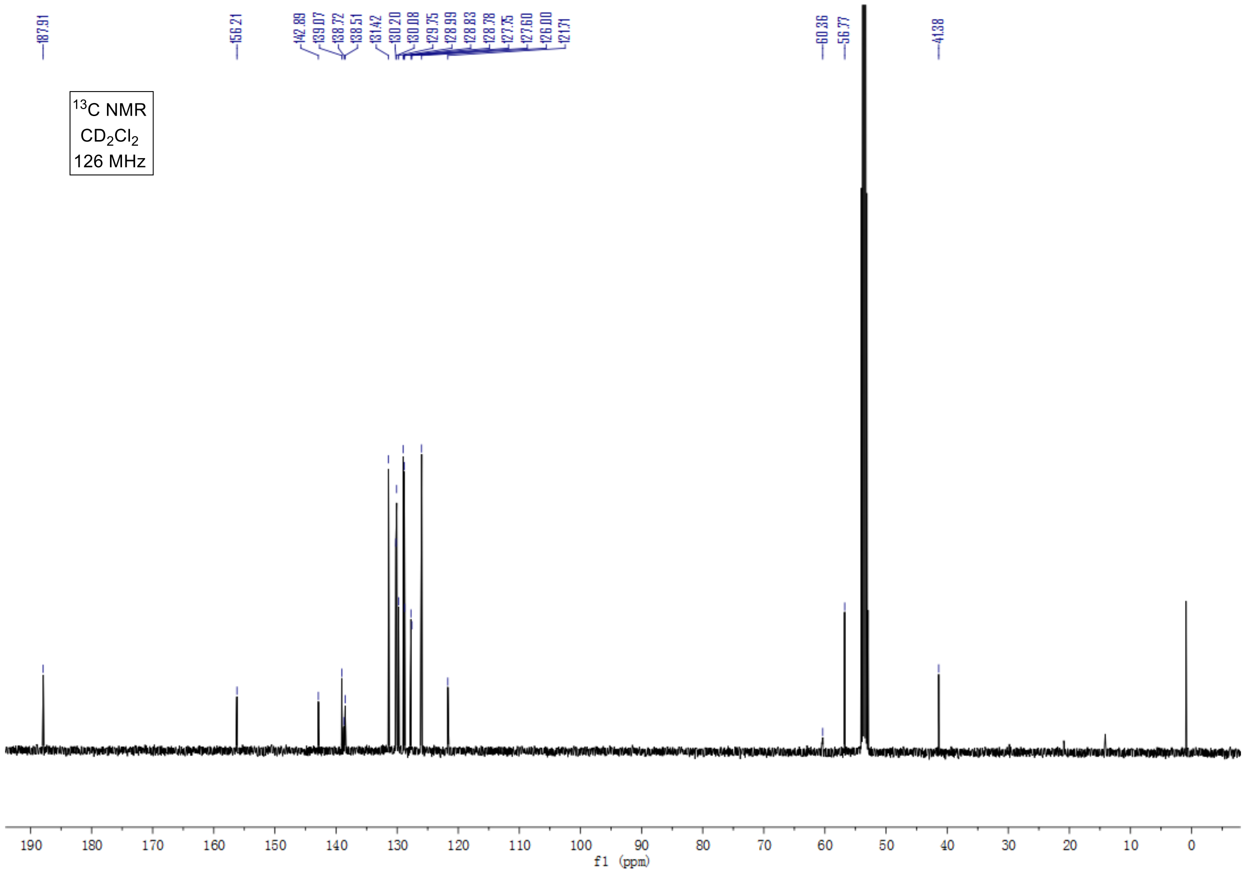

^{13}C NMR
CD_2Cl_2
126 MHz
f1 (ppm)

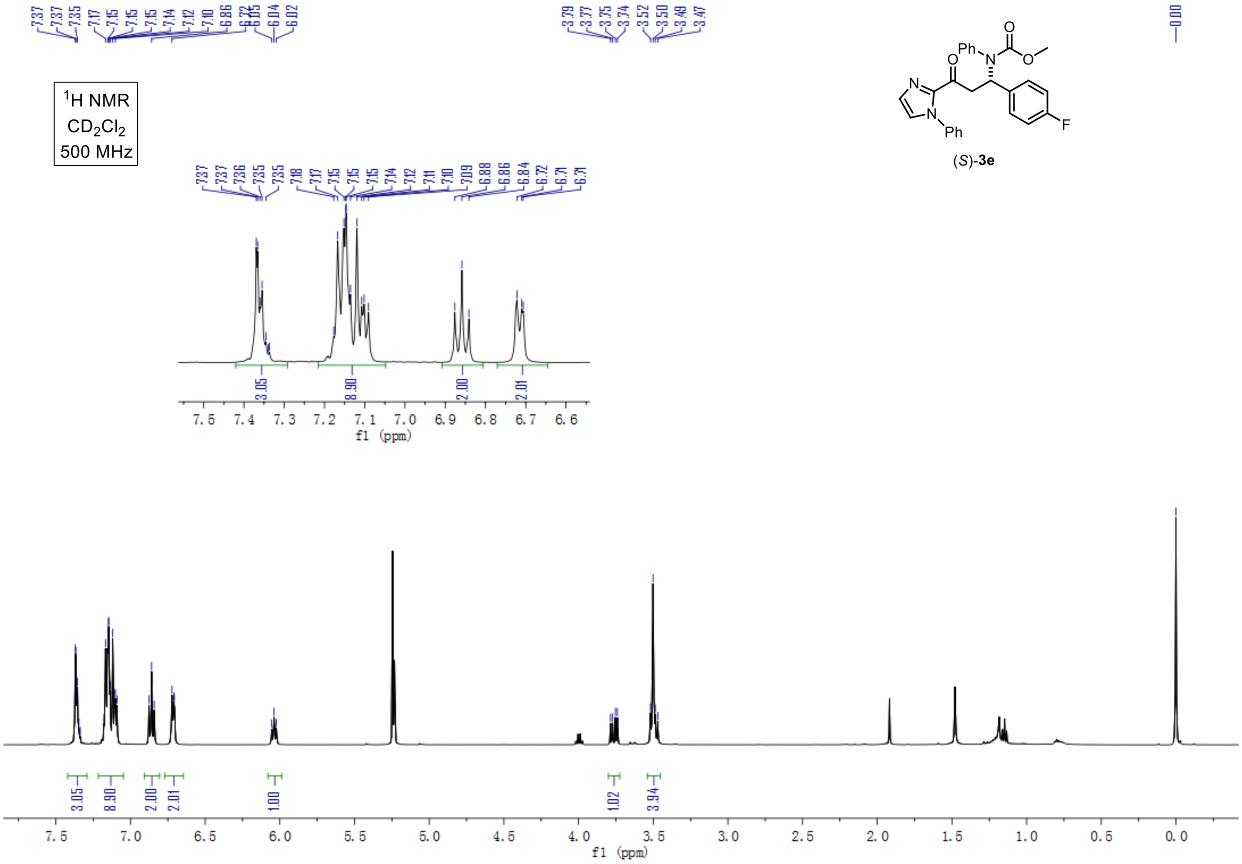

1H NMR
CD2Cl2
500 MHz
3.05
8.90
2.00
2.01
1.00
1.02
3.94
f1 (ppm)
Ph
N
O
O
N
N
Ph
F

(S)-**3e**

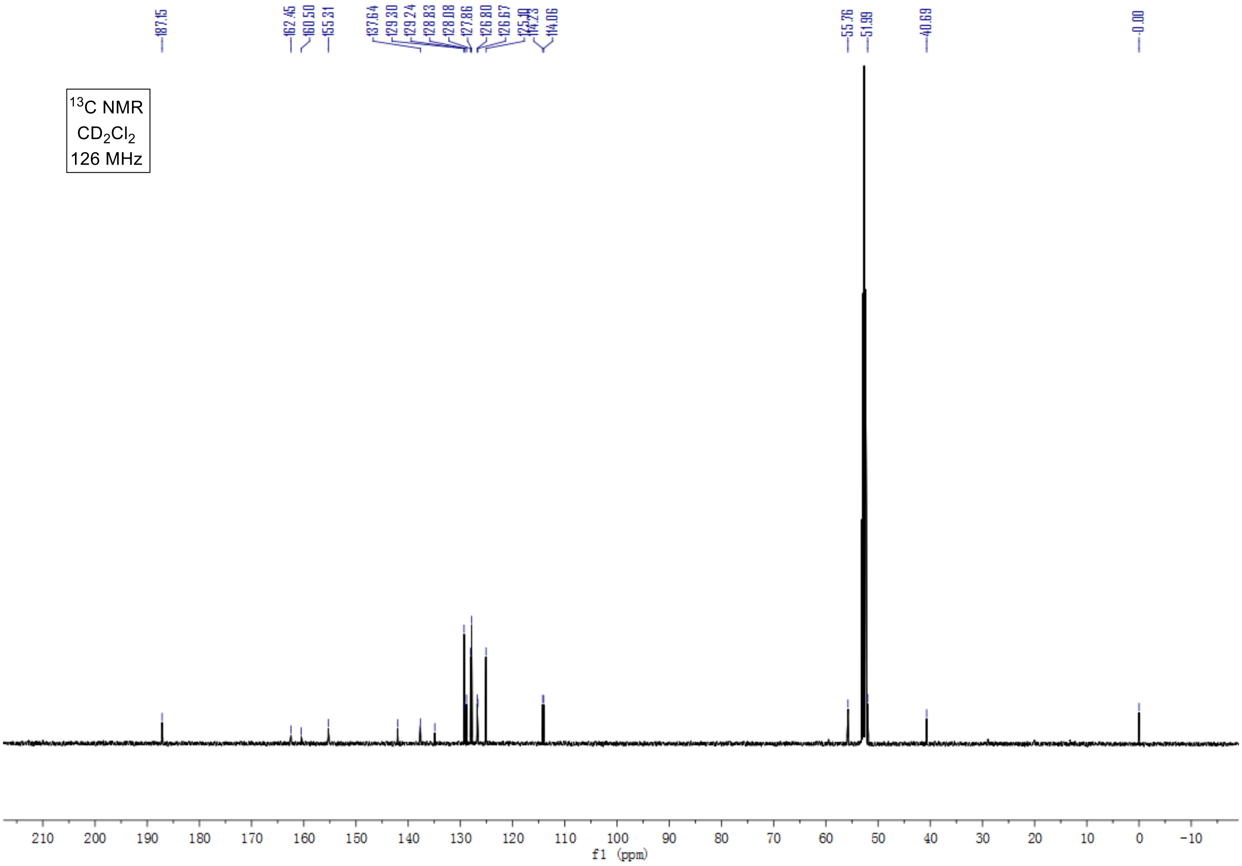

13C NMR
CD2Cl2
126 MHz
187.15
162.45
160.50
155.31
137.64
129.30
129.24
128.83
128.08
127.86
126.80
126.67
114.06
55.76
51.99
40.69
0.00
f1 (ppm)

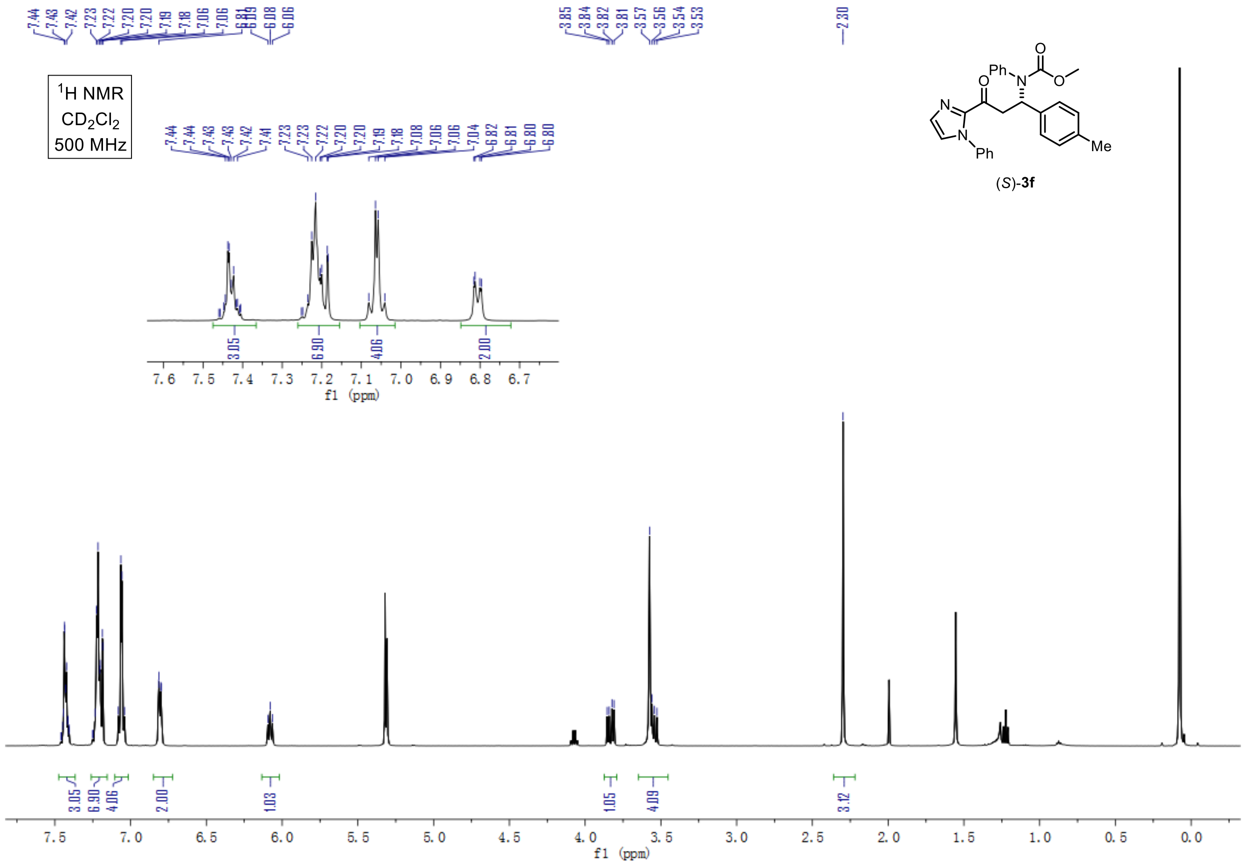
^{1}H NMR
CD_2Cl_2
500 MHz
(S)-3f
f1 (ppm)

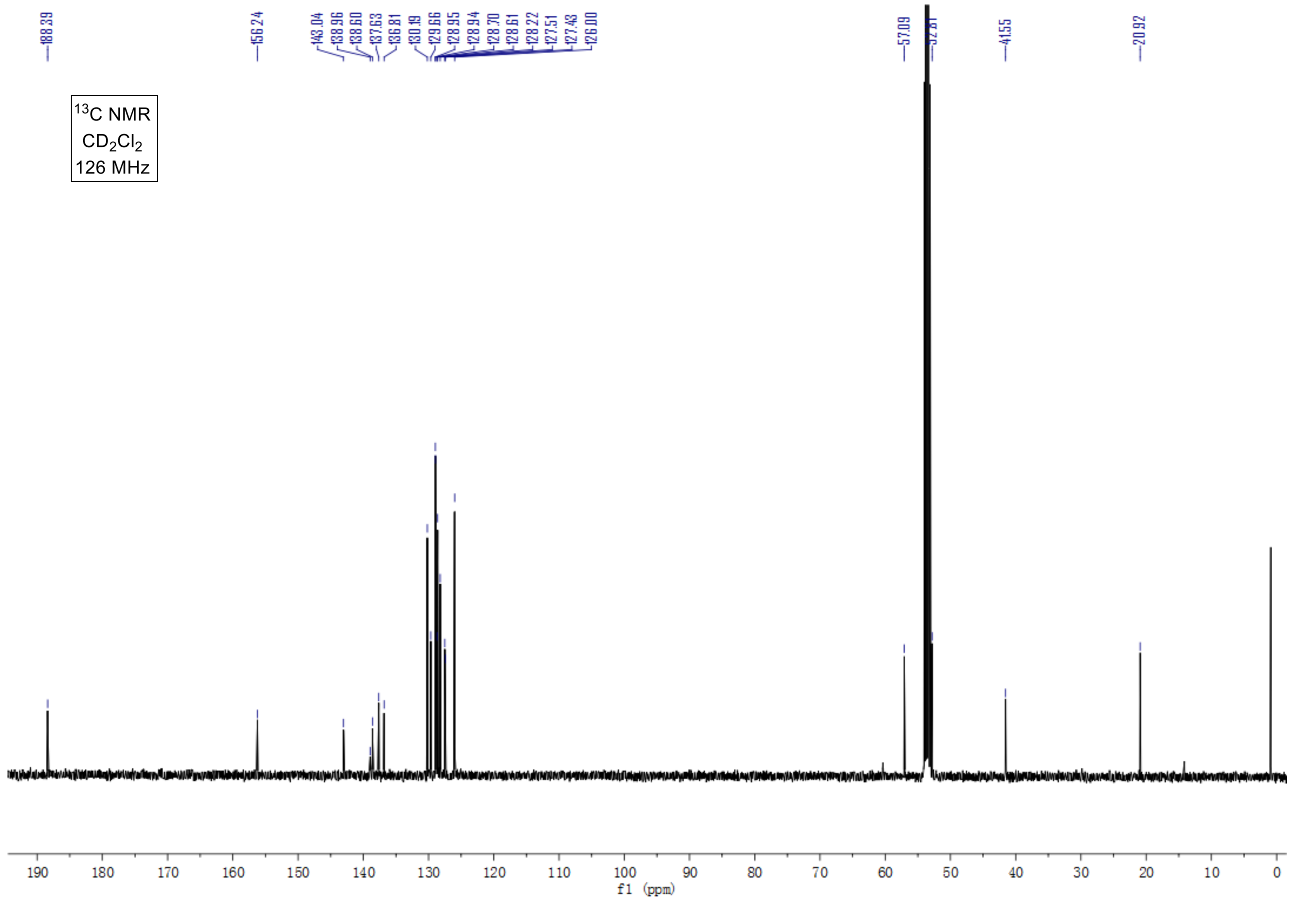
^{13}C NMR
CD_2Cl_2
126 MHz
f1 (ppm)

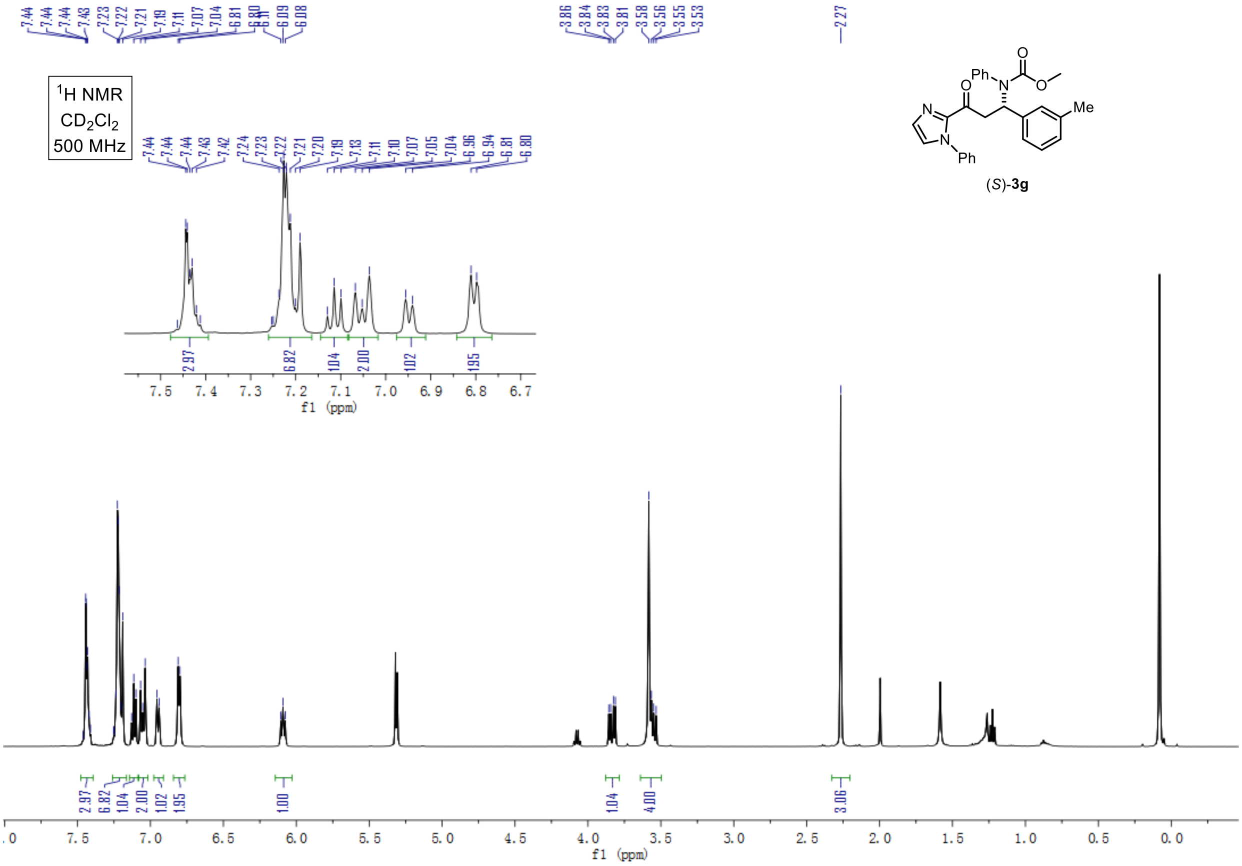

¹H NMR
CD2Cl2
500 MHz
f1 (ppm)
Ph
N
O
O
O
Me
N
N
Ph

(*S*)-**3g**

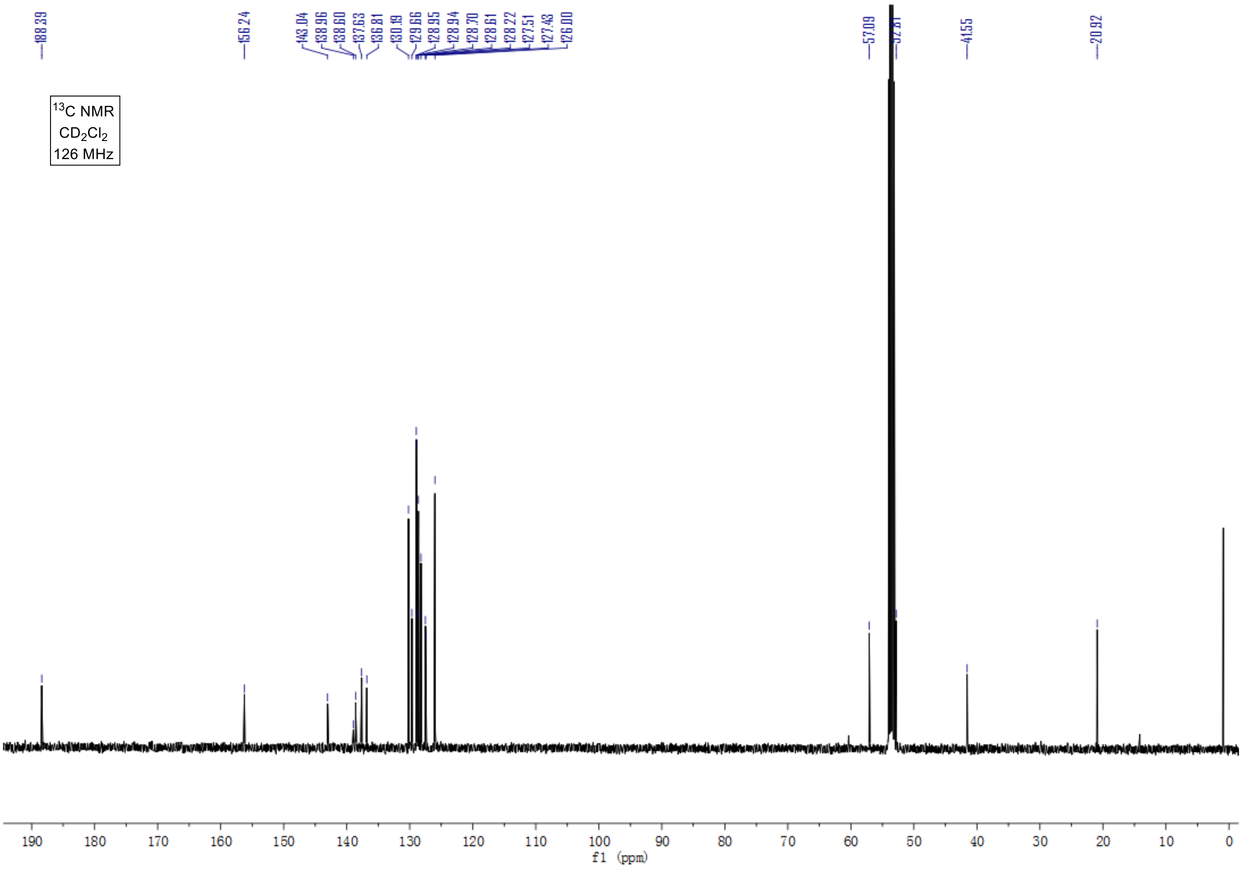

¹³C NMR
CD2Cl2
126 MHz
f1 (ppm)

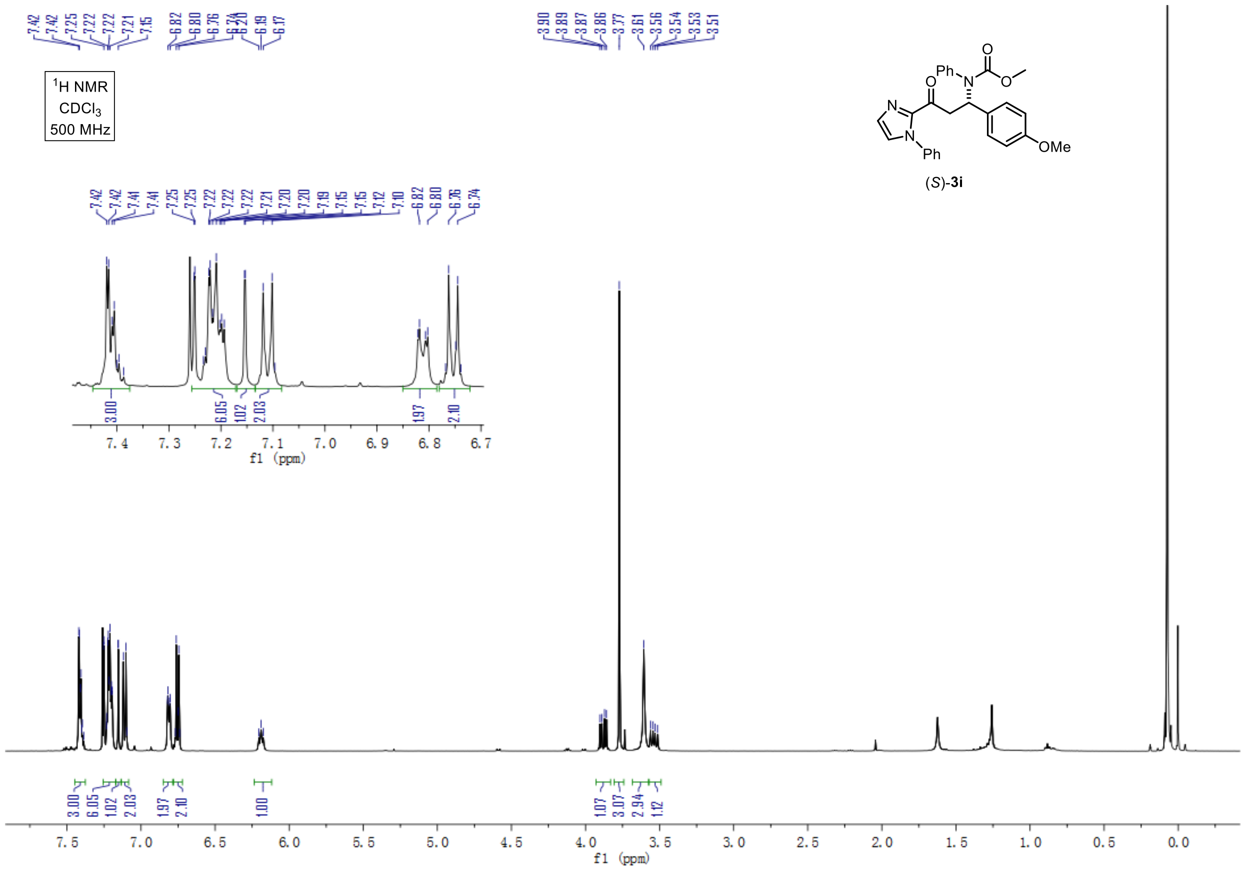

^{1}H NMR
$CDCl_3$
500 MHz
f1 (ppm)
N
N
Ph
O
Ph
N
O
O
OMe

(*S*)-**3i**

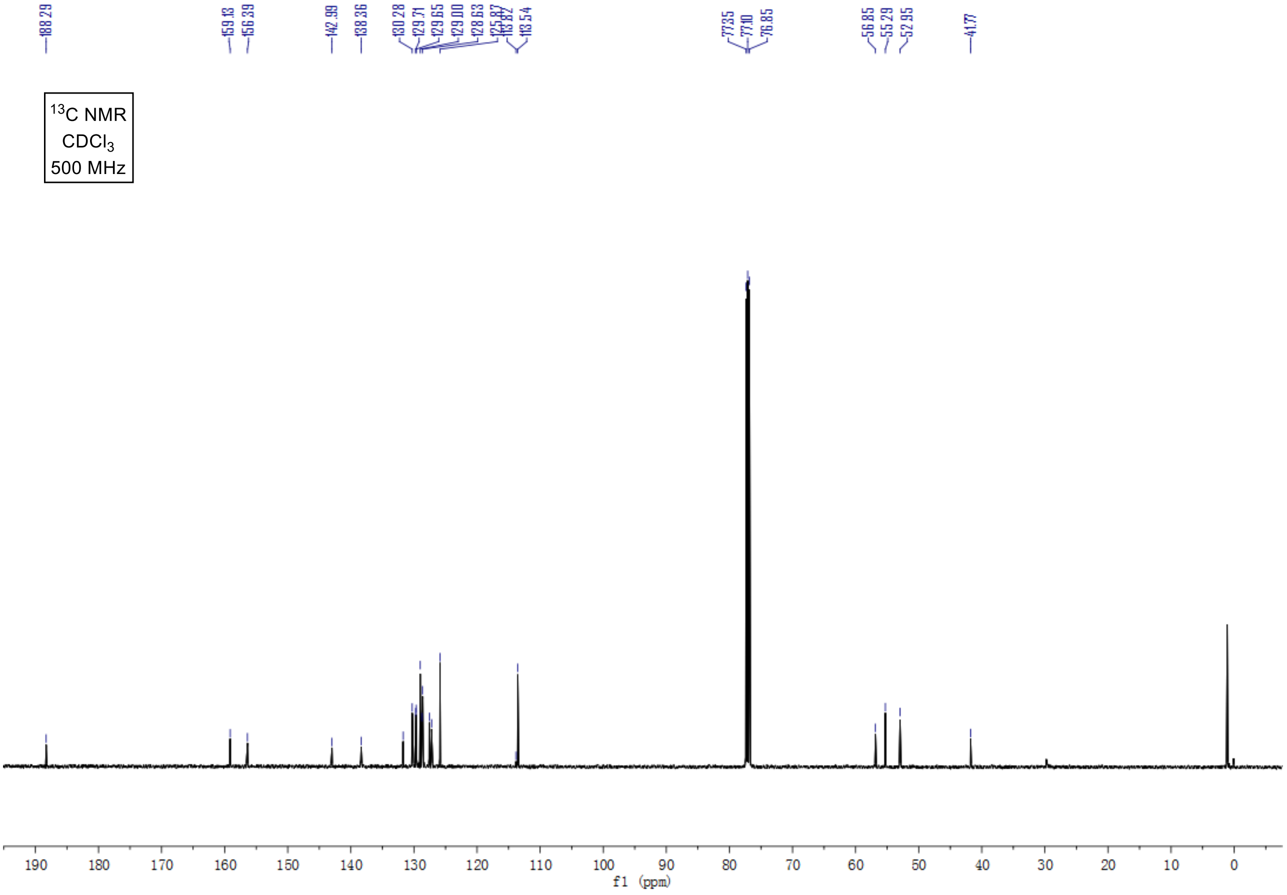

^{13}C NMR
$CDCl_3$
500 MHz
f1 (ppm)

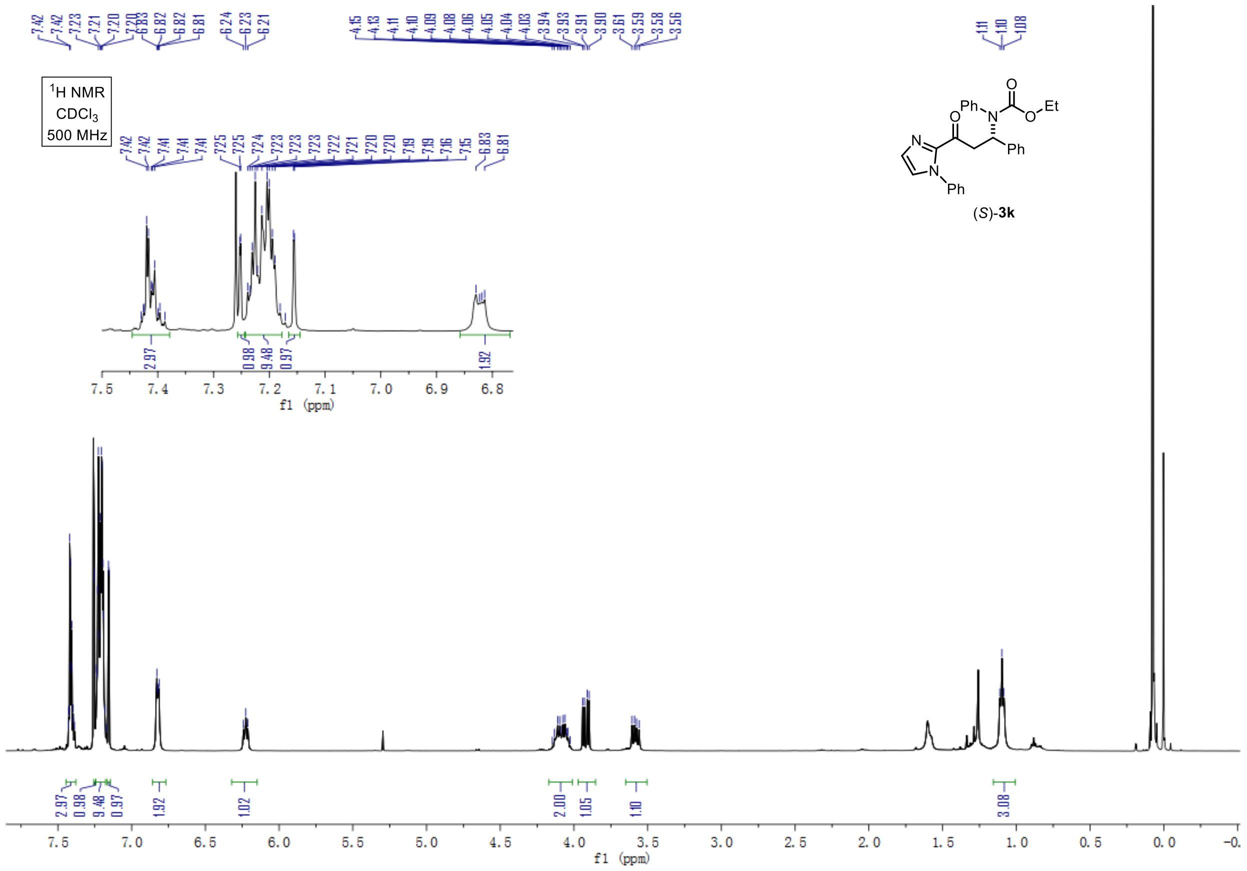
^{1}H NMR
$CDCl_3$
500 MHz
(S)-3k

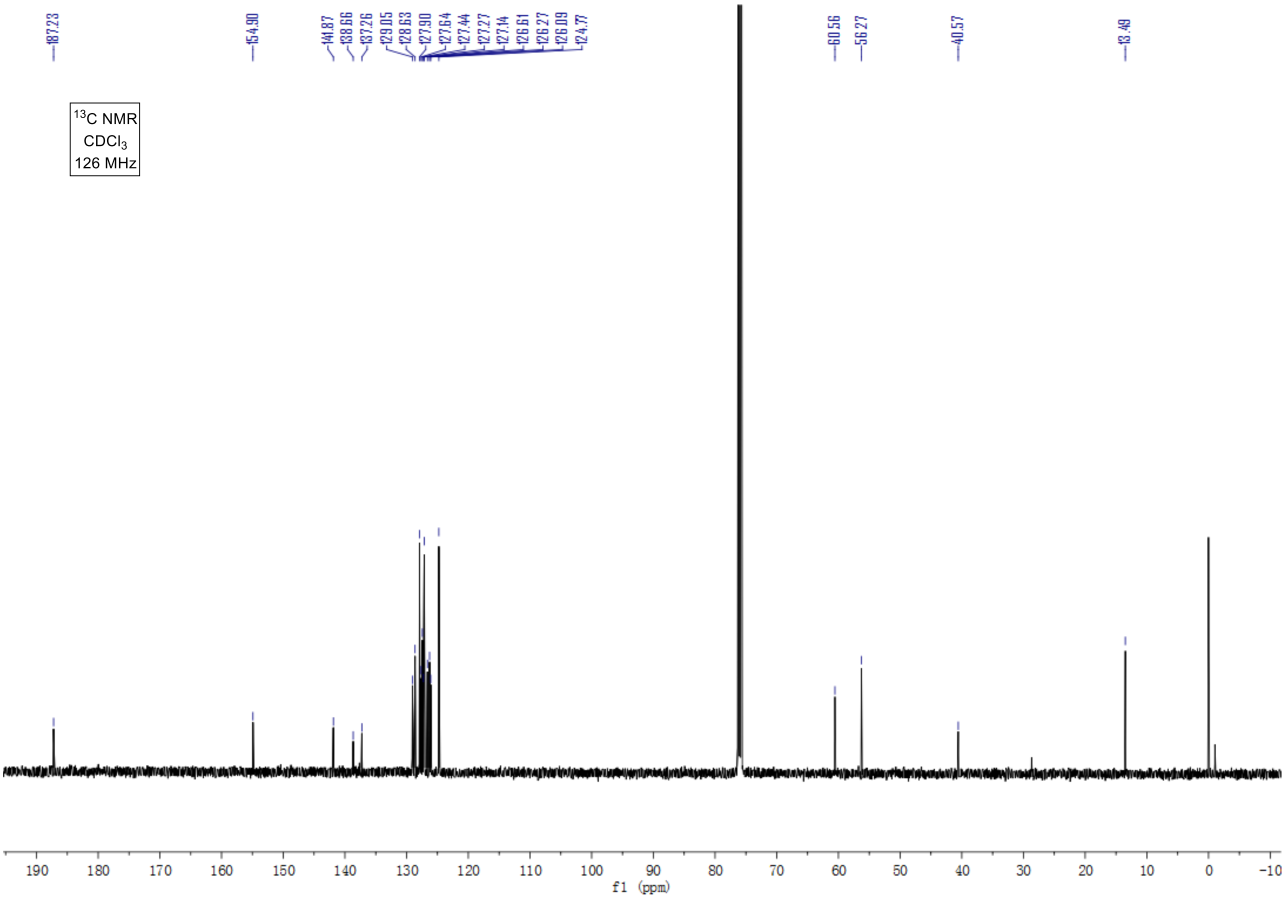
^{13}C NMR
$CDCl_3$
126 MHz

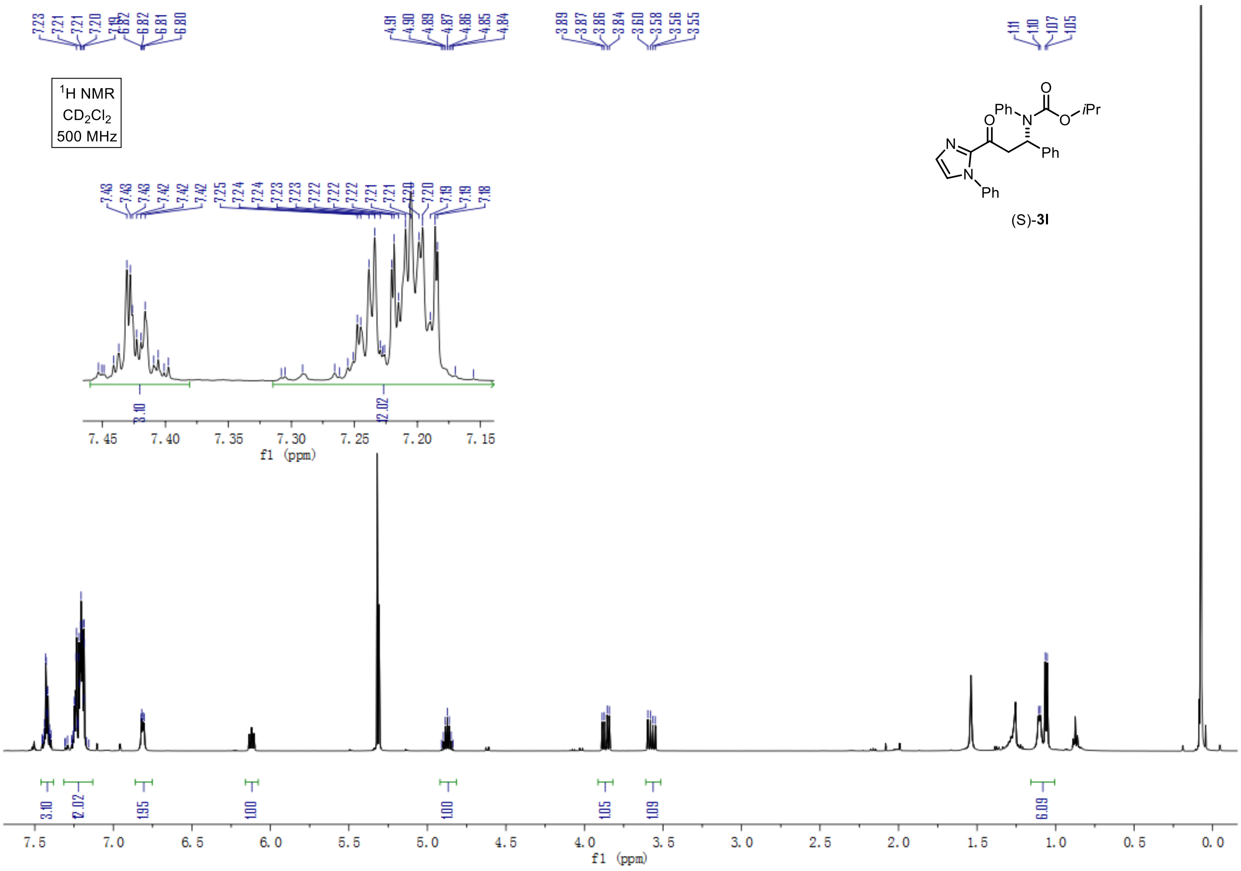
¹H NMR
CD_2Cl_2
500 MHz
f1 (ppm)
Ph
N
O
O
iPr
N
N
Ph
Ph

(S)-**3l**

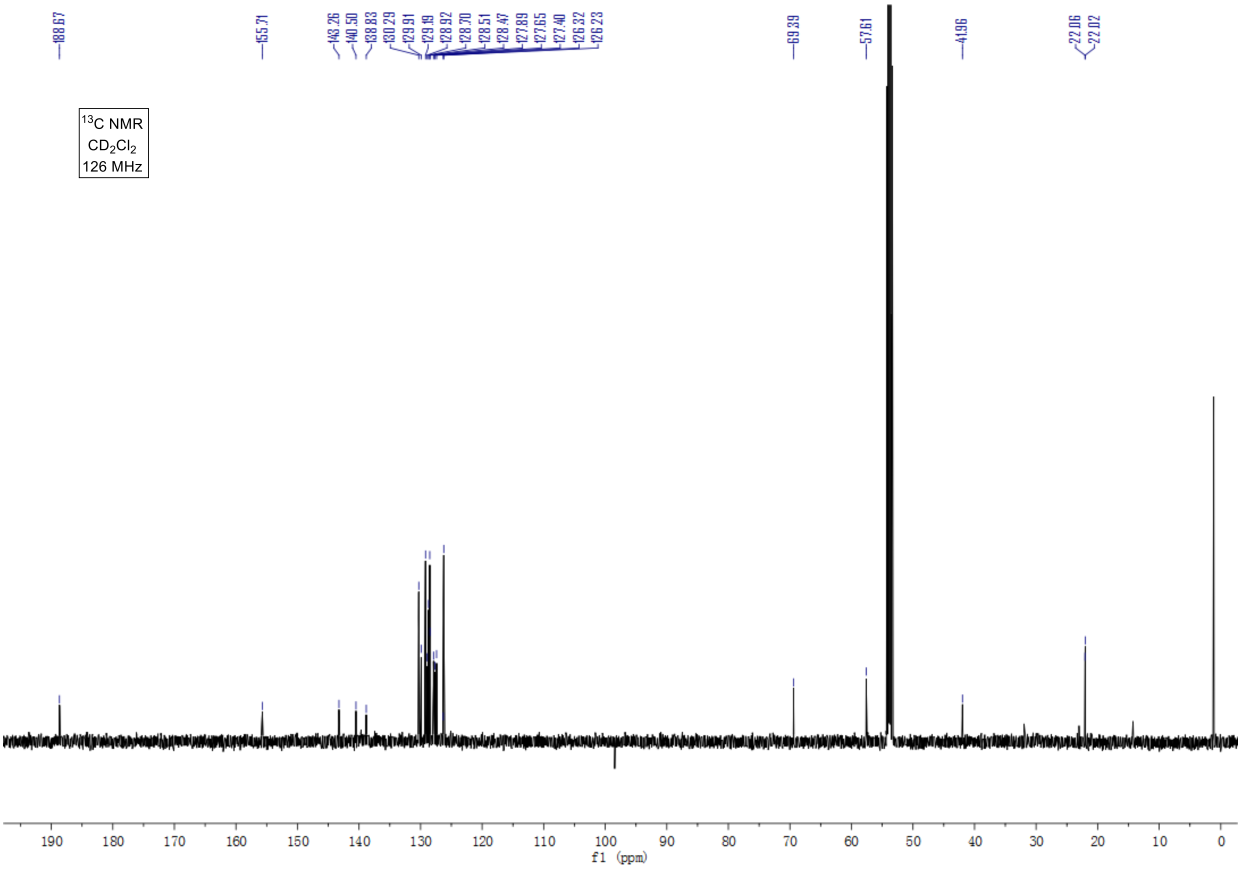
¹³C NMR
CD_2Cl_2
126 MHz
188.67
155.71
143.26
140.50
138.83
130.29
129.91
129.19
128.92
128.70
128.51
128.47
127.89
127.65
127.40
126.32
126.23
69.39
57.61
41.96
22.06
22.02
f1 (ppm)

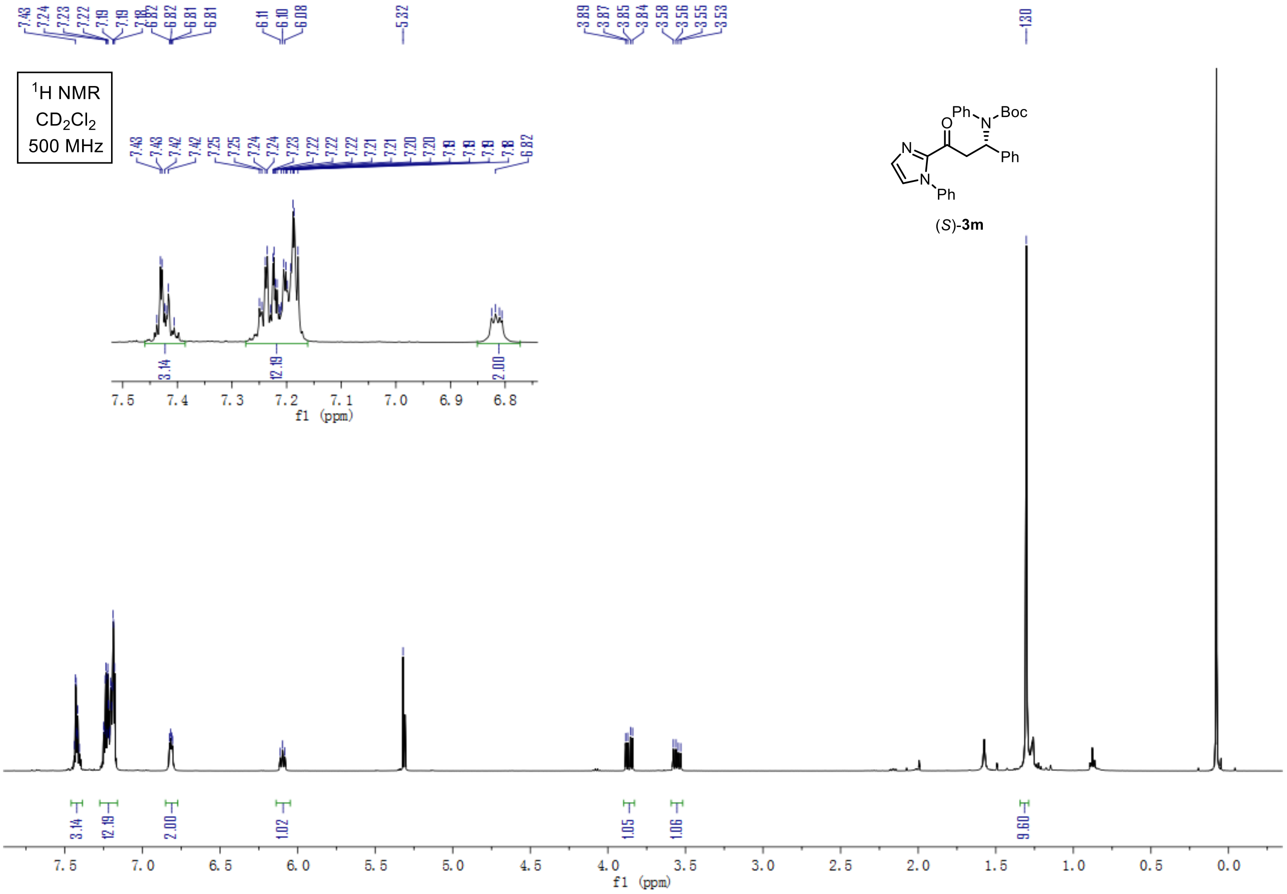

1H NMR
CD_2Cl_2
500 MHz
Ph
N
Boc
O
N
N
Ph
Ph
(S)-3m
7.43 7.24 7.23 7.22 7.19 7.19 7.18 6.82 6.81 6.81 6.11 6.10 6.08 5.32 3.89 3.87 3.85 3.84 3.58 3.56 3.55 3.53 1.30
3.14 12.19 2.00 1.02 1.05 1.06 9.60
f1 (ppm)

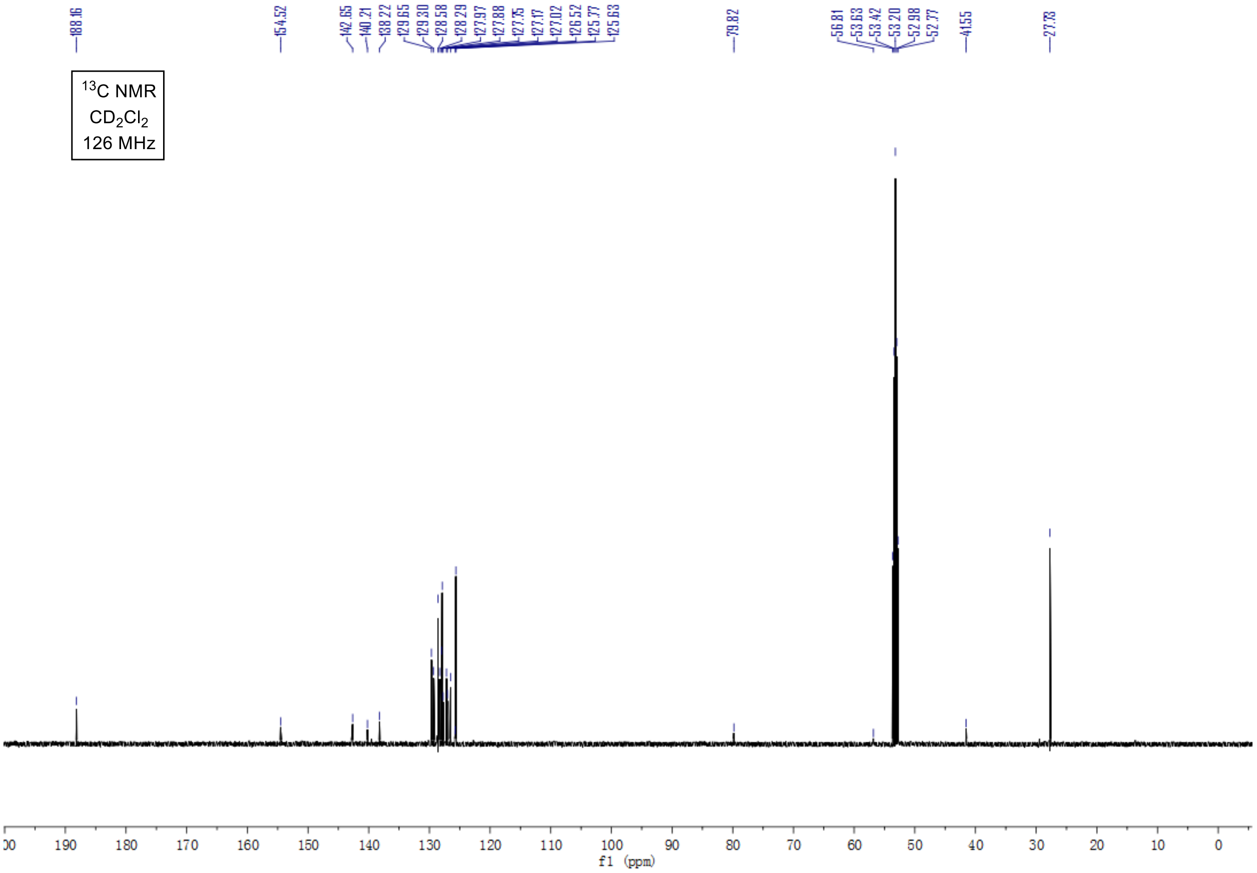

13C NMR
CD_2Cl_2
126 MHz
188.16 154.52 142.65 140.21 138.22 129.65 129.30 128.58 128.29 127.97 127.88 127.75 127.17 127.02 126.52 125.77 125.63 79.82 56.81 53.63 53.42 53.20 52.98 52.77 41.55 27.73
f1 (ppm)

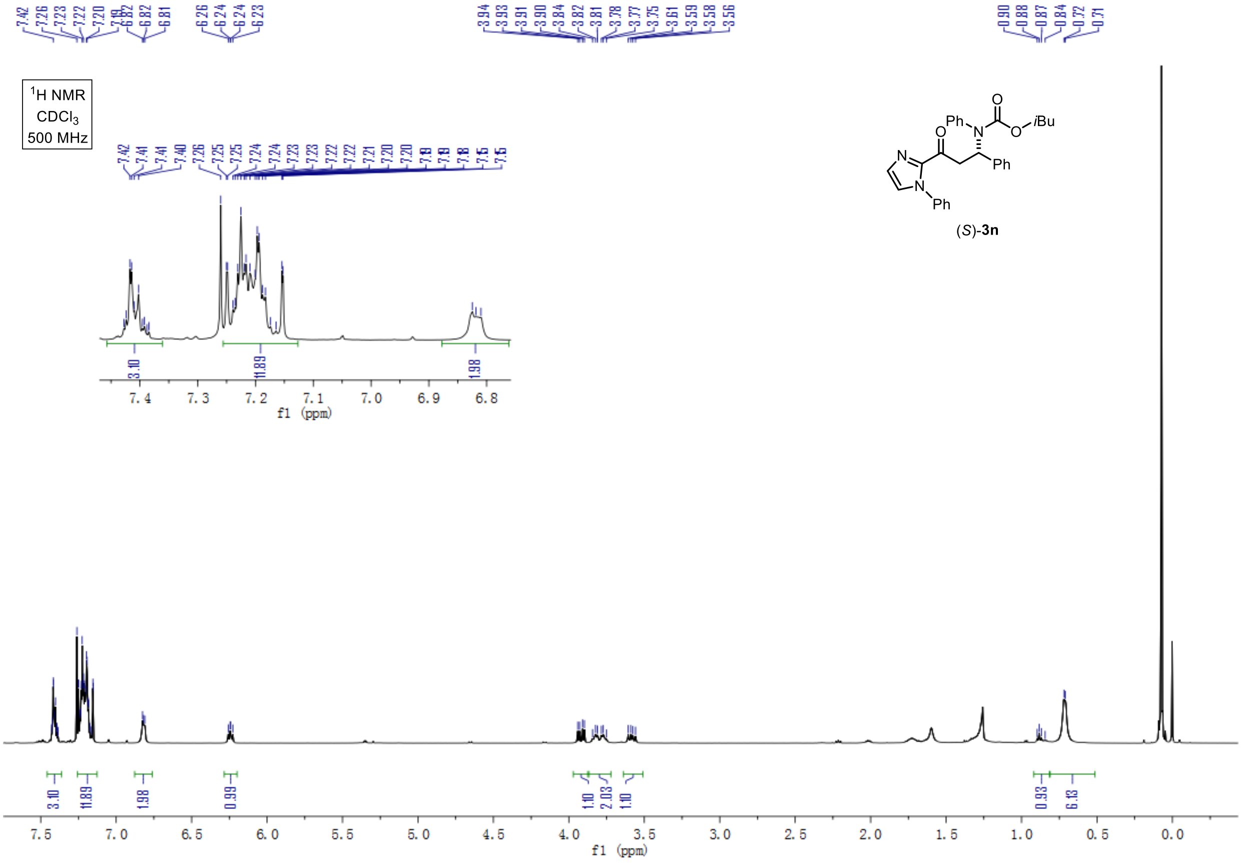
1H NMR
CDCl3
500 MHz
Ph
N
O
O
iBu
O
N
N
Ph
Ph
(S)-3n
f1 (ppm)

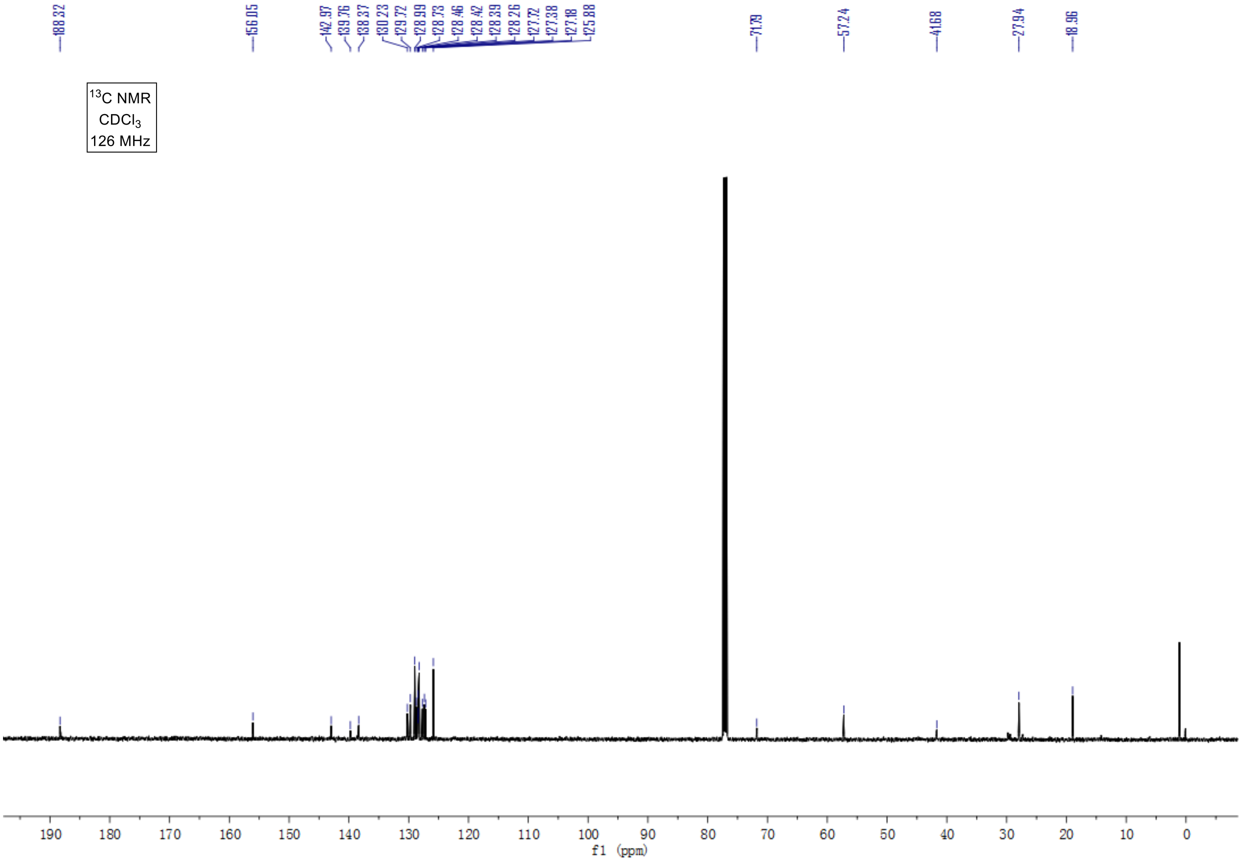
13C NMR
CDCl3
126 MHz
f1 (ppm)

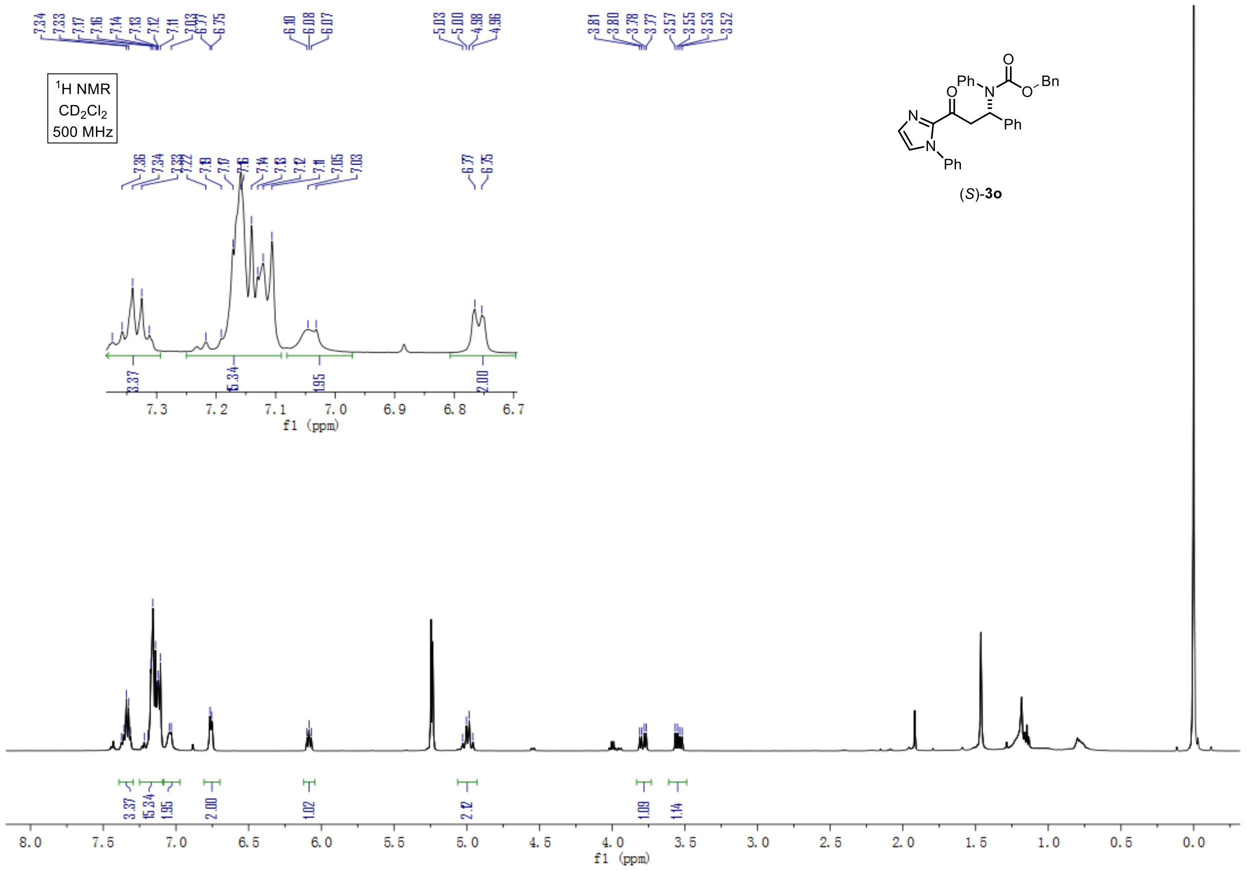

(S)-3o
¹H NMR
CD₂Cl₂
500 MHz
f1 (ppm)

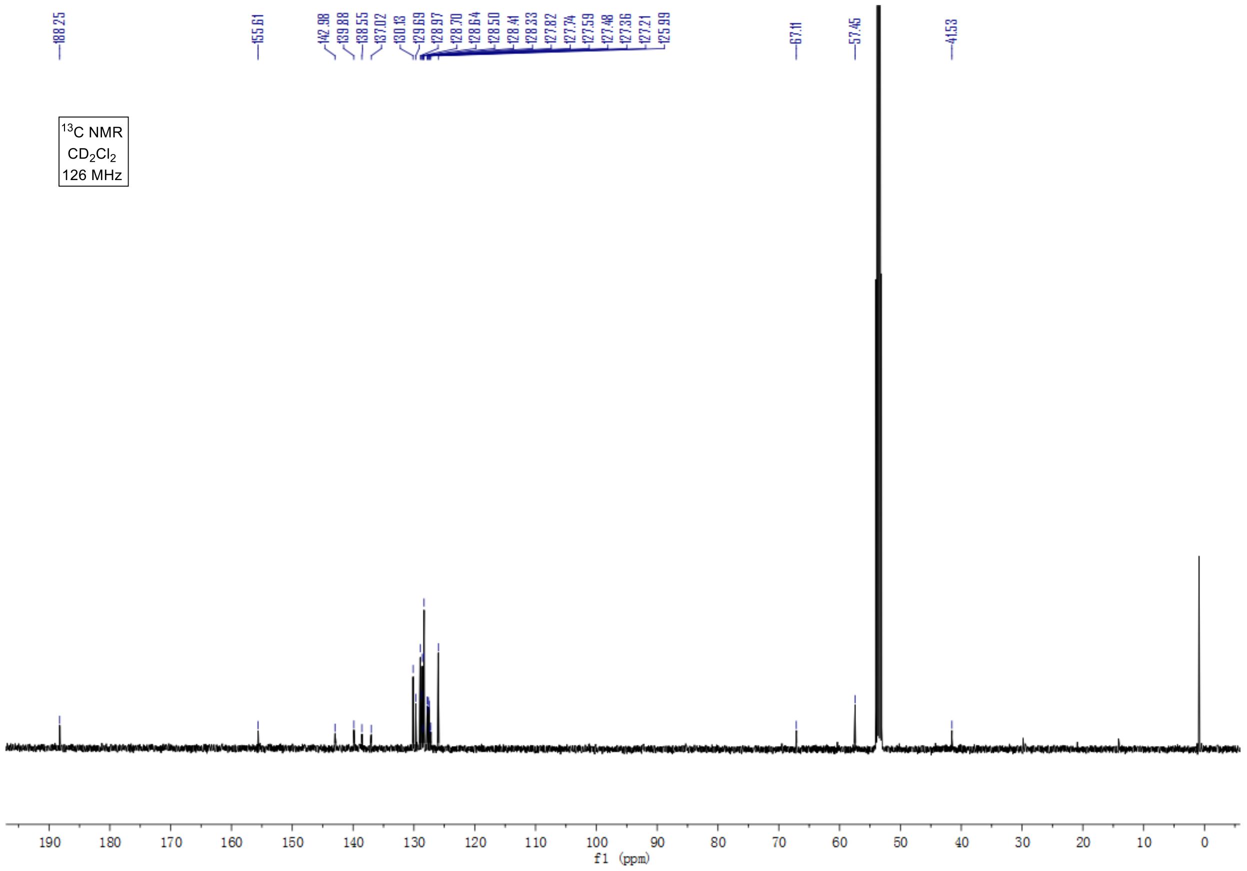

¹³C NMR
CD₂Cl₂
126 MHz
f1 (ppm)

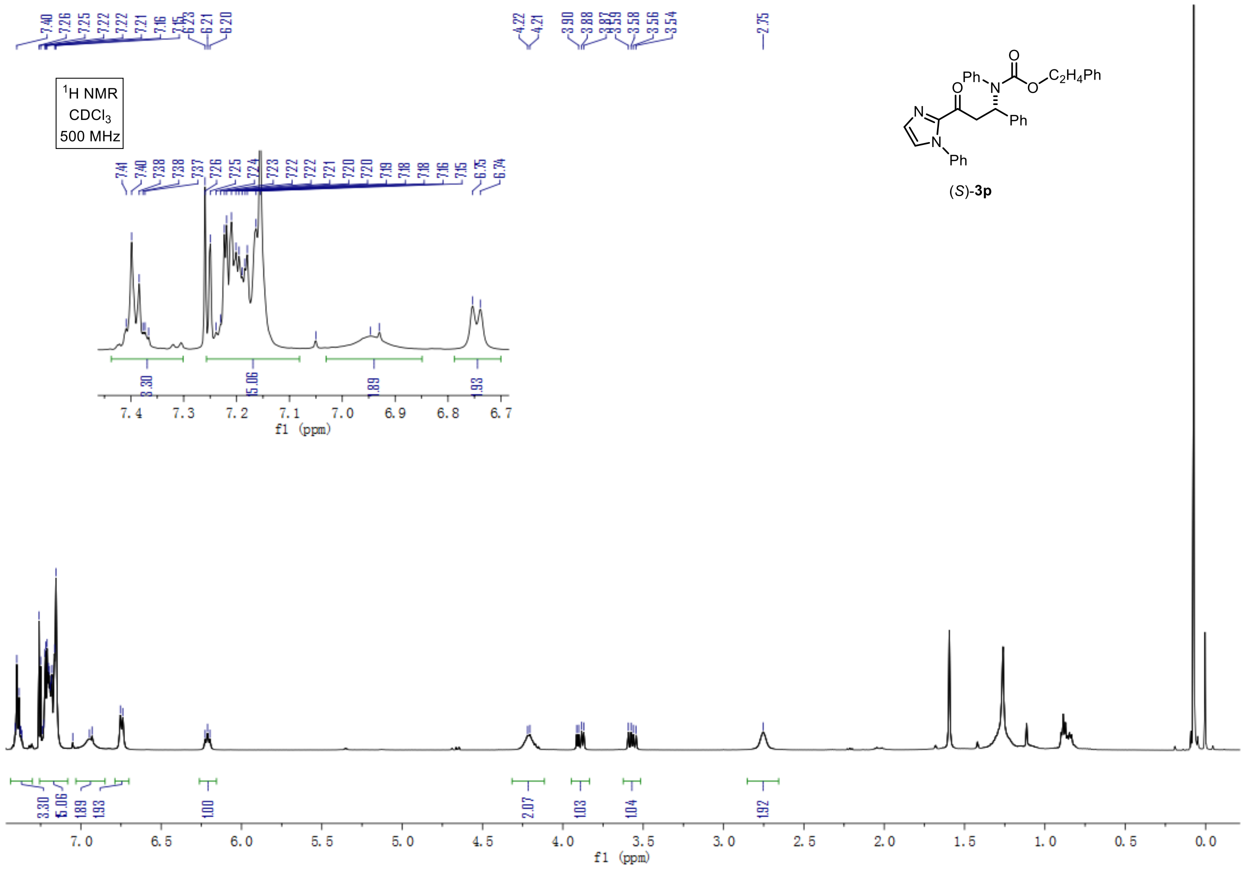

(*S*)-**3p**

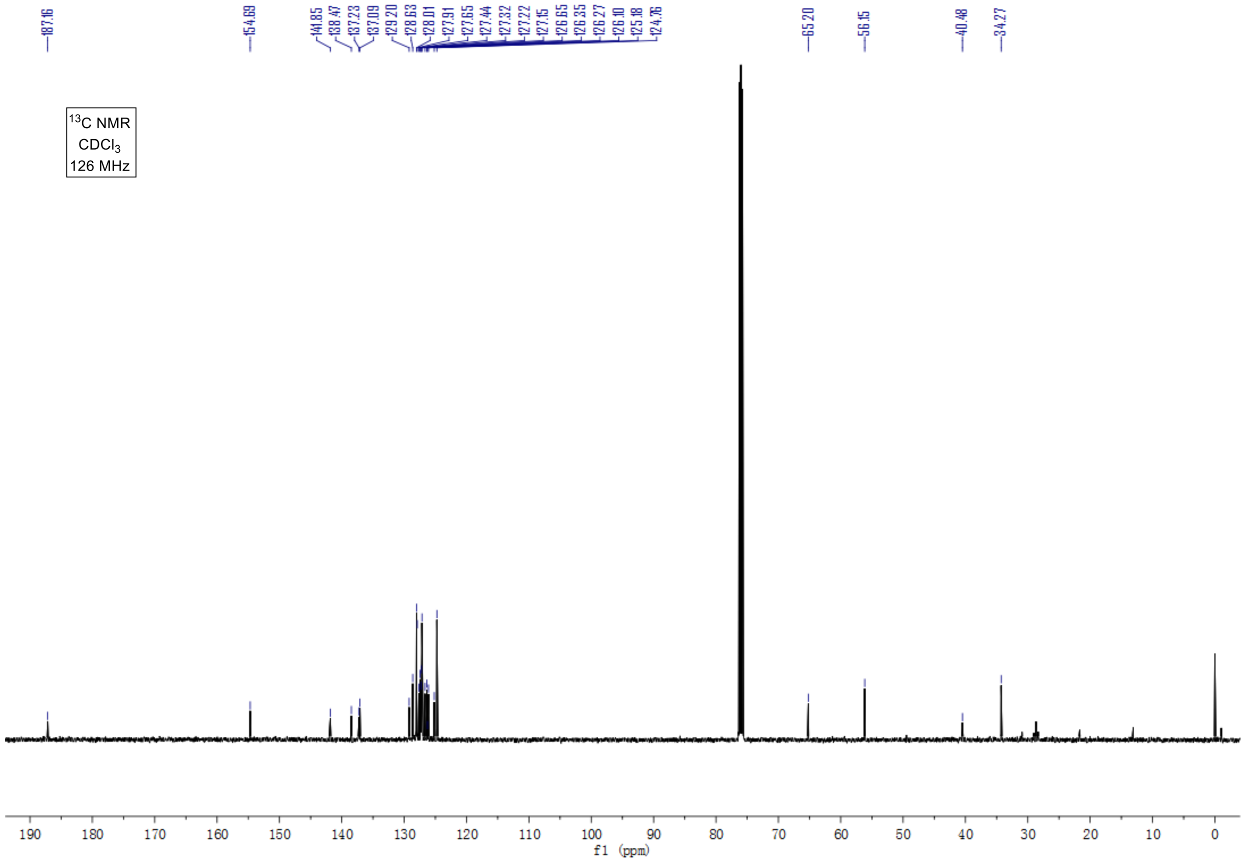

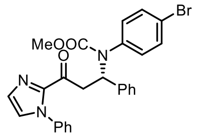
Br
MeOOC
N
O
N
N
Ph
Ph

(*S*)-**3r**

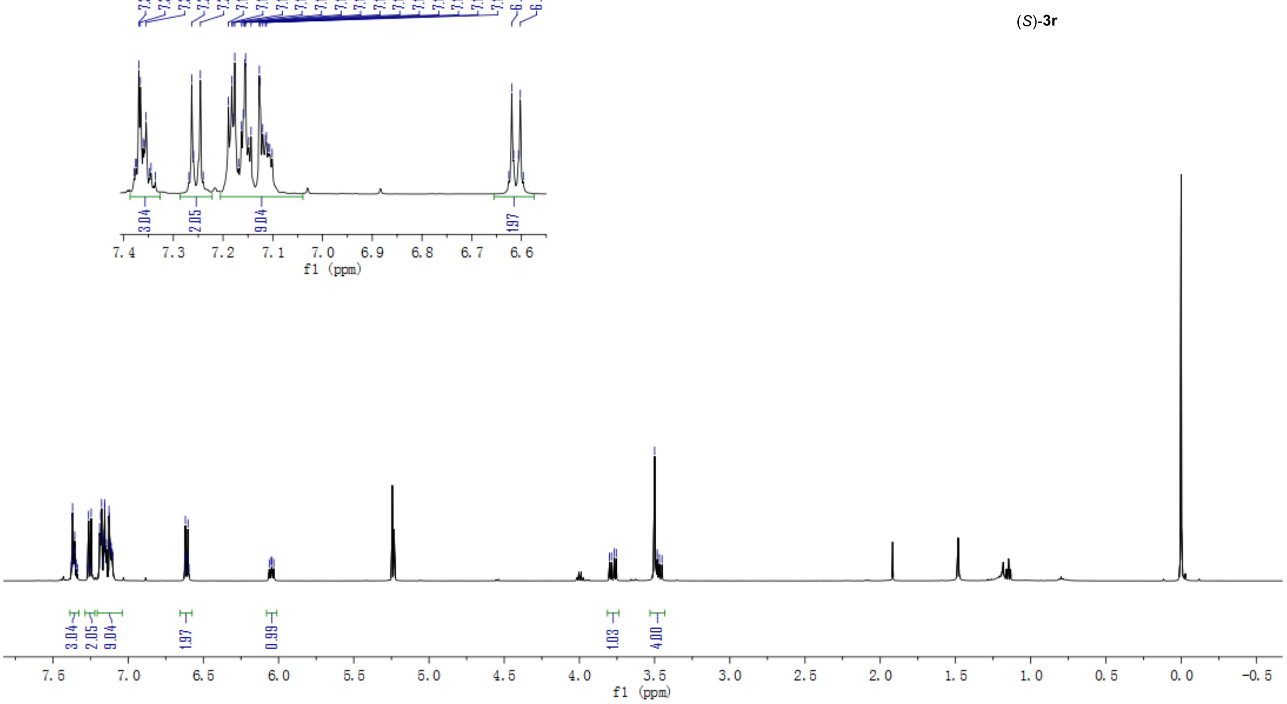
1H NMR
CD2Cl2
500 MHz
f1 (ppm)

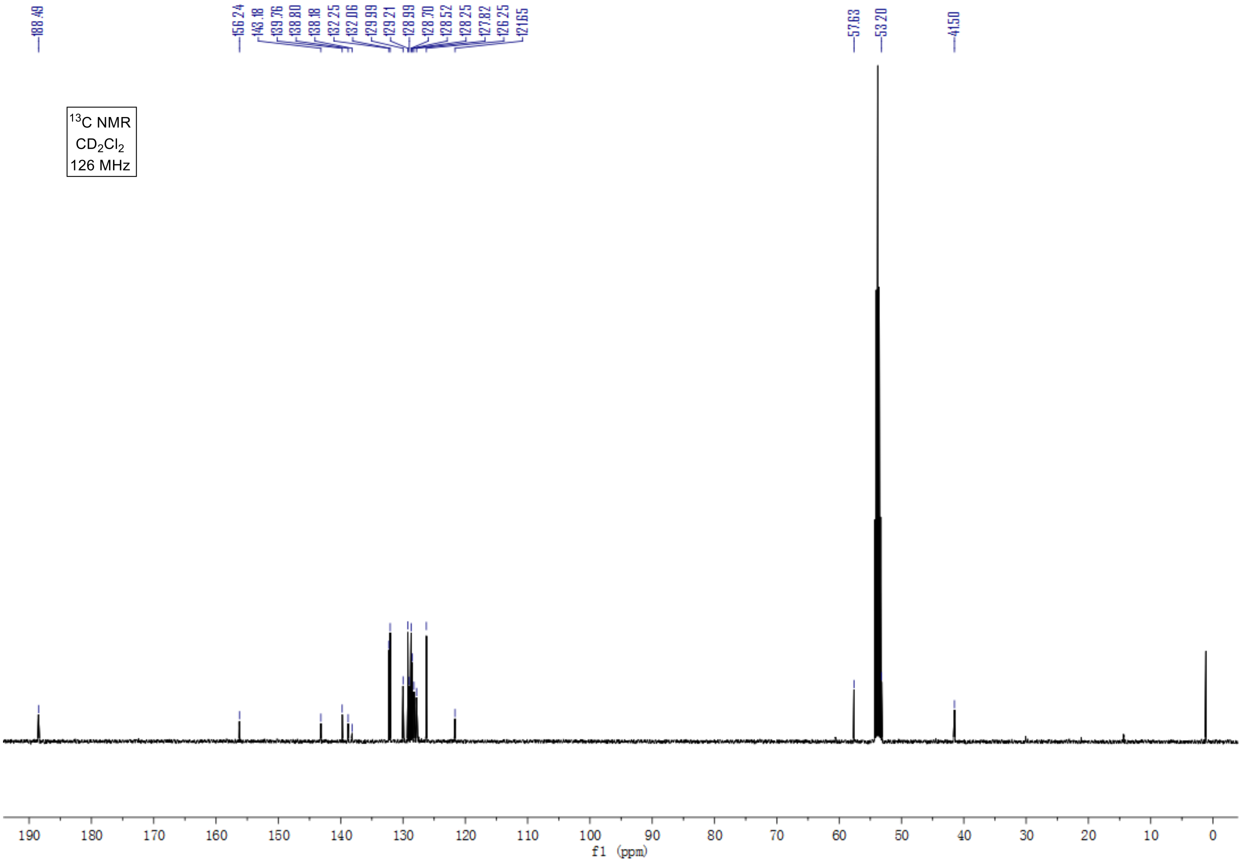
13C NMR
CD2Cl2
126 MHz
f1 (ppm)

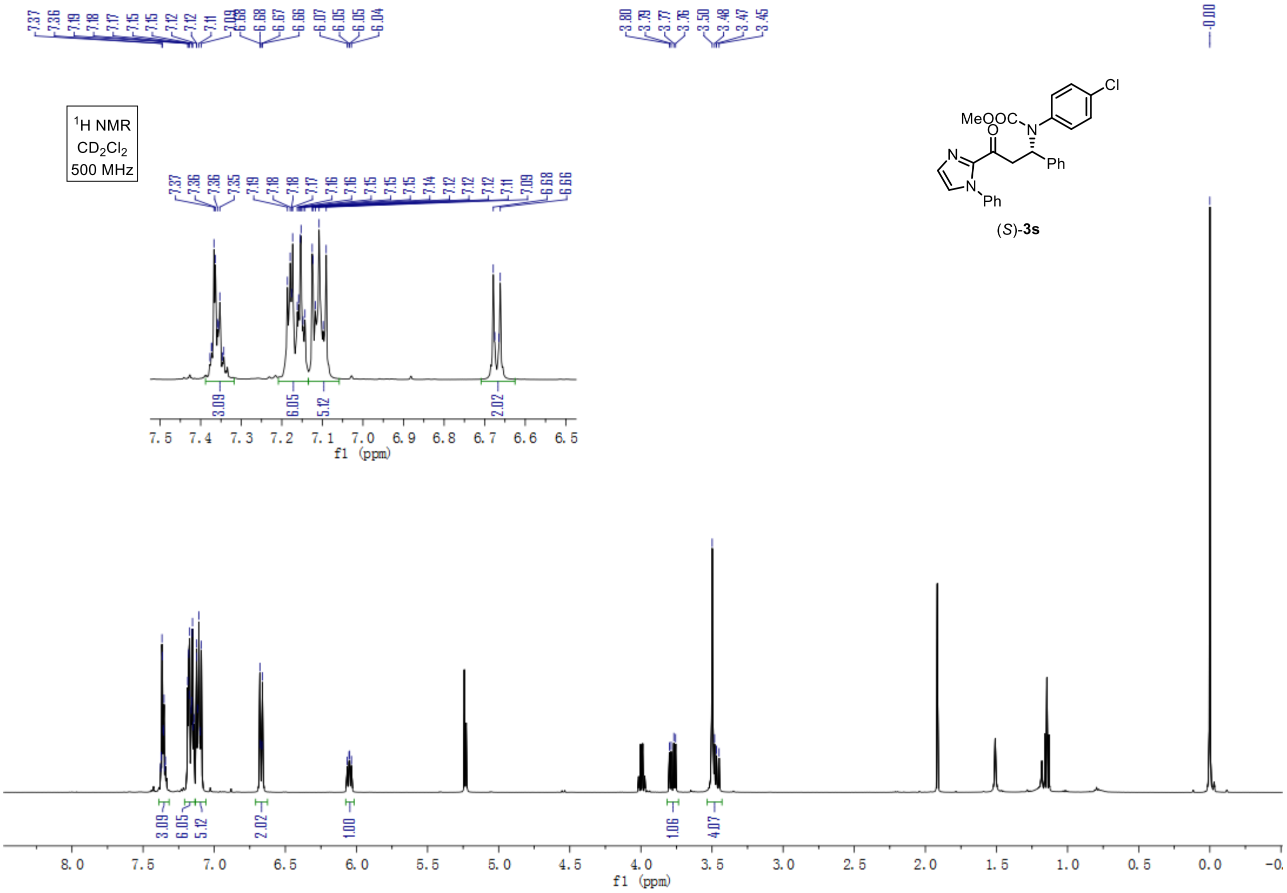
1H NMR
CD2Cl2
500 MHz
MeOOC
N
Cl
O
N
N
Ph
Ph
(S)-3s
f1 (ppm)

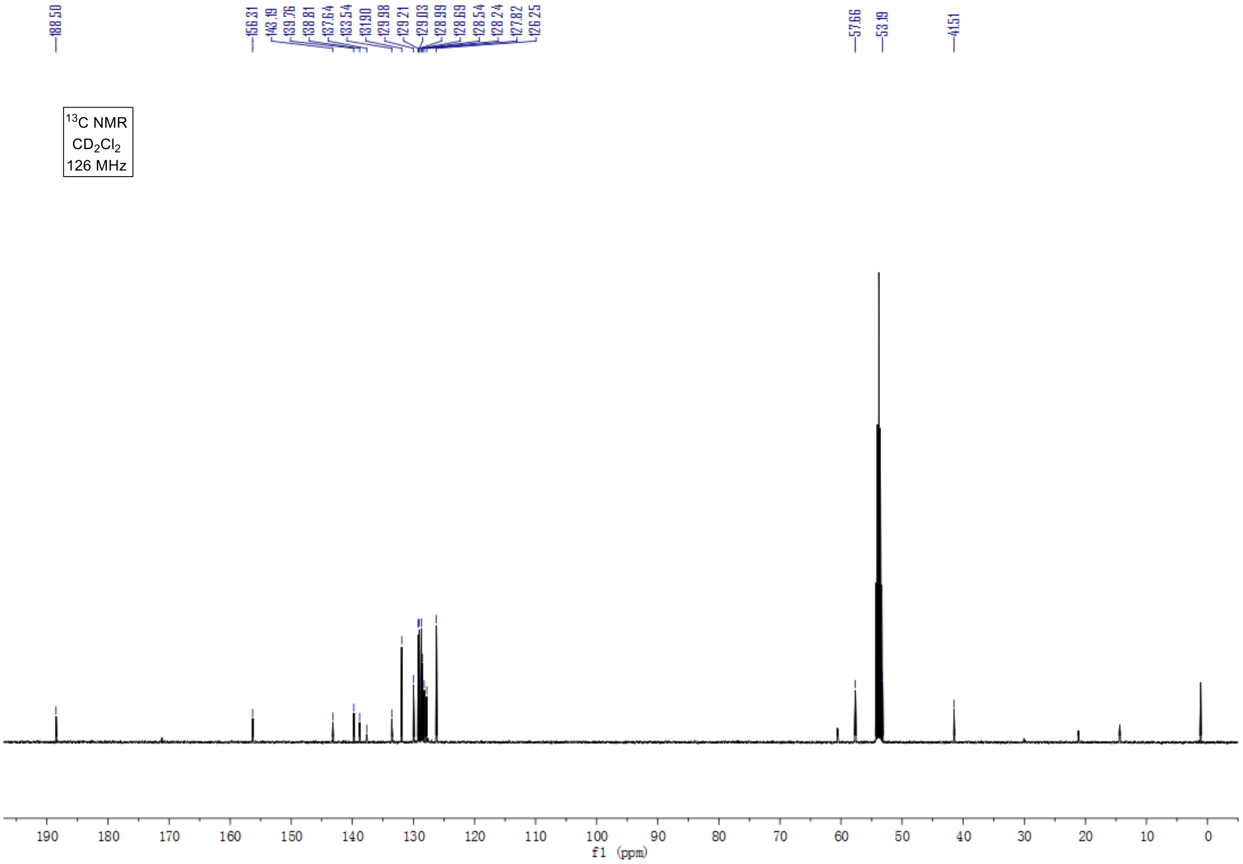
13C NMR
CD2Cl2
126 MHz
f1 (ppm)

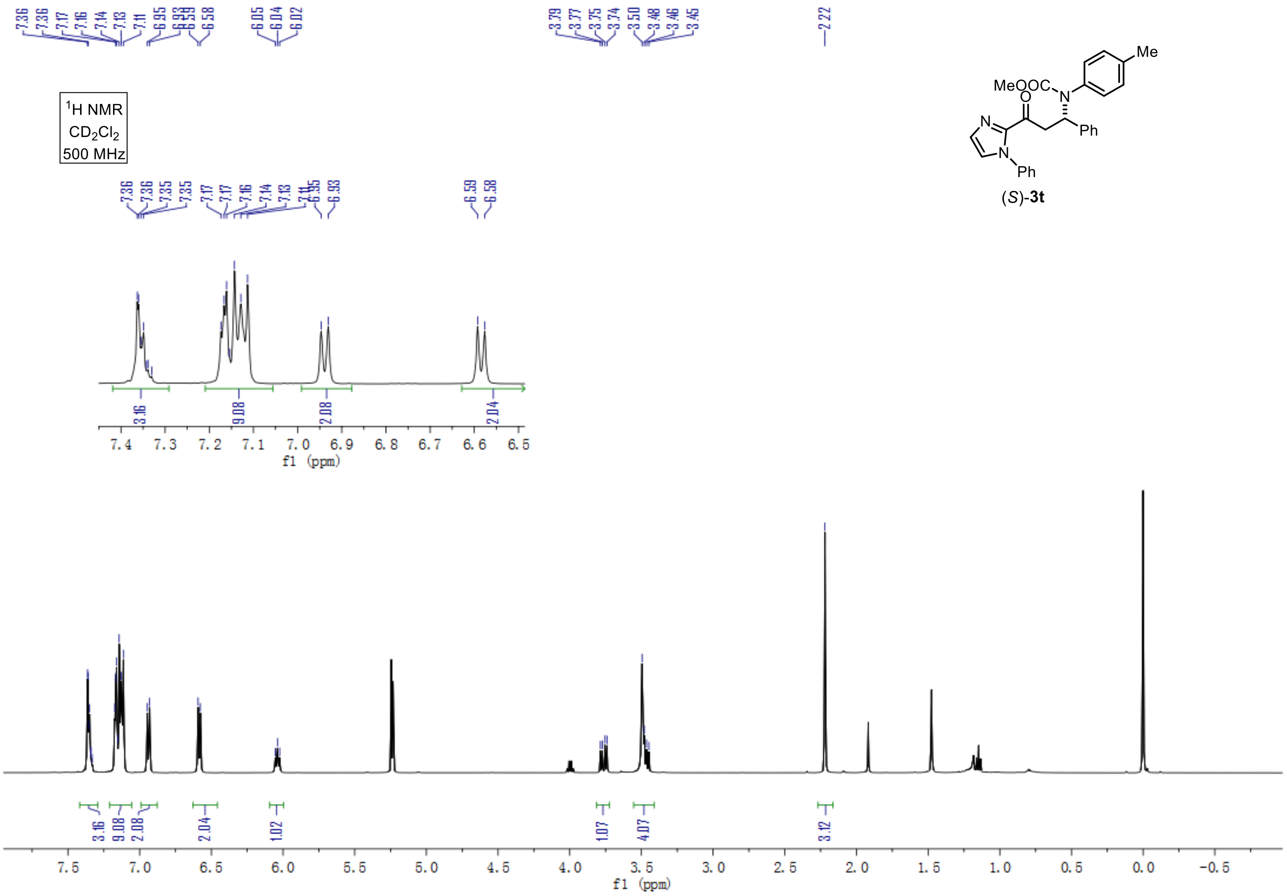
^{1}H NMR
CD_2Cl_2
500 MHz
MeOOC
N
Me
O
N
Ph
N
Ph

(*S*)-**3t**

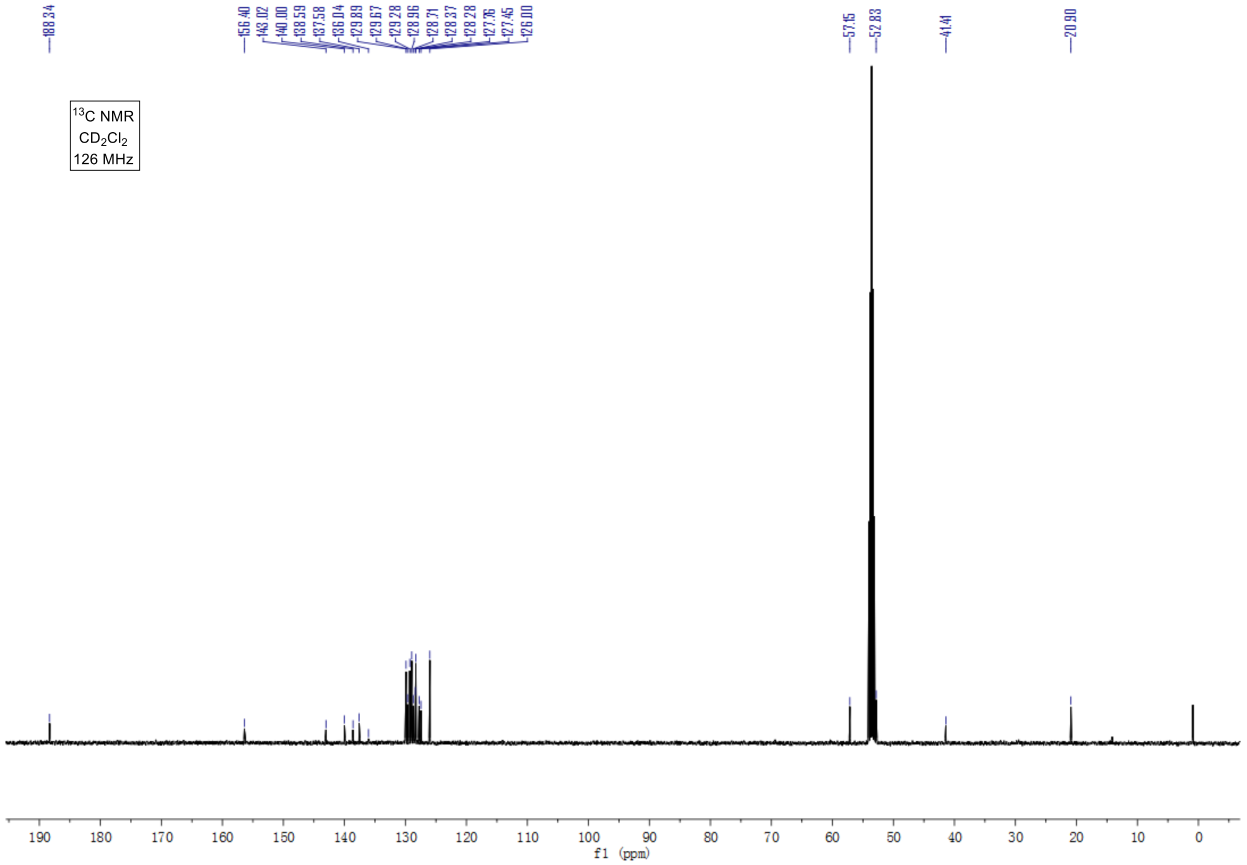
^{13}C NMR
CD_2Cl_2
126 MHz

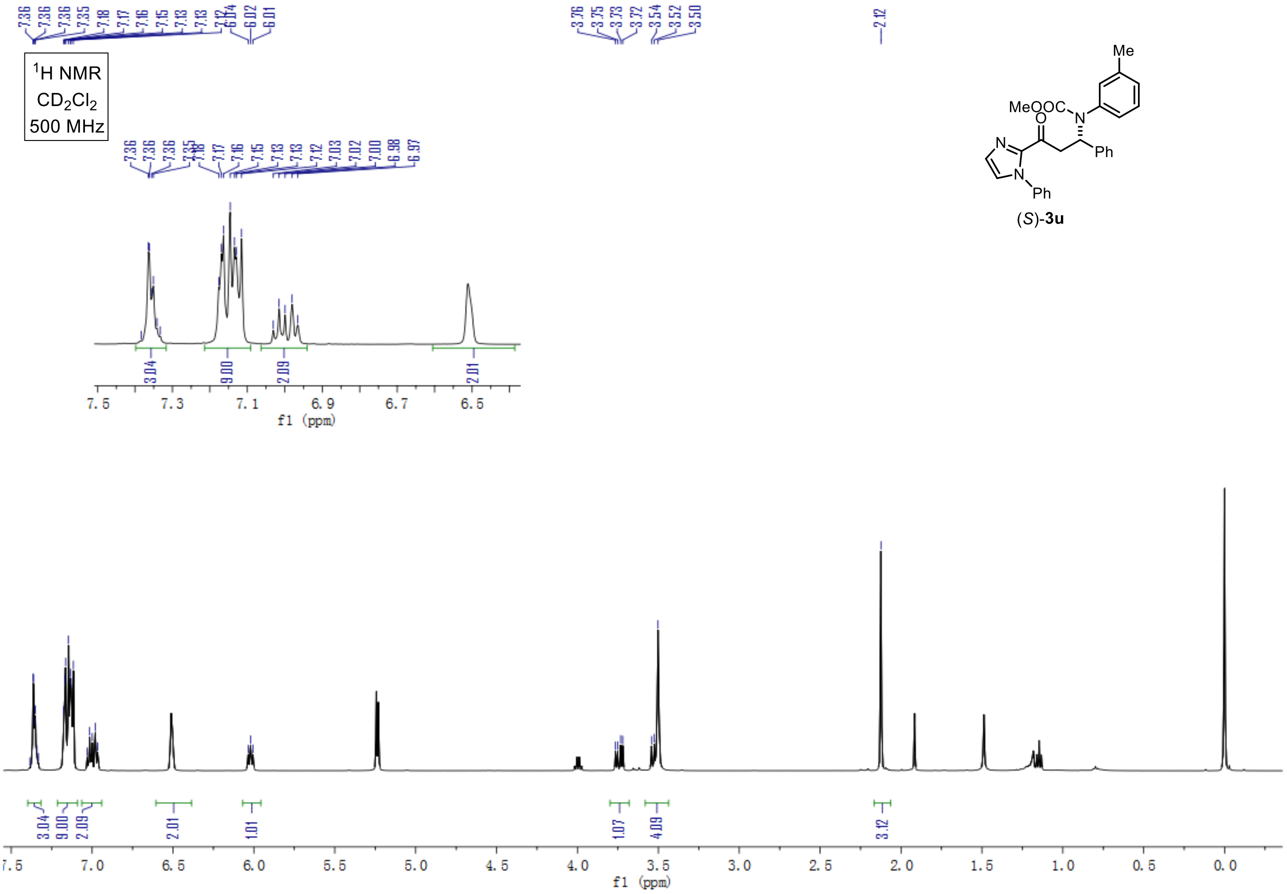

^1H NMR
CD_2Cl_2
500 MHz
Me
MeOOC
N
O
N
N
Ph
Ph
f1 (ppm)

(*S*)-**3u**

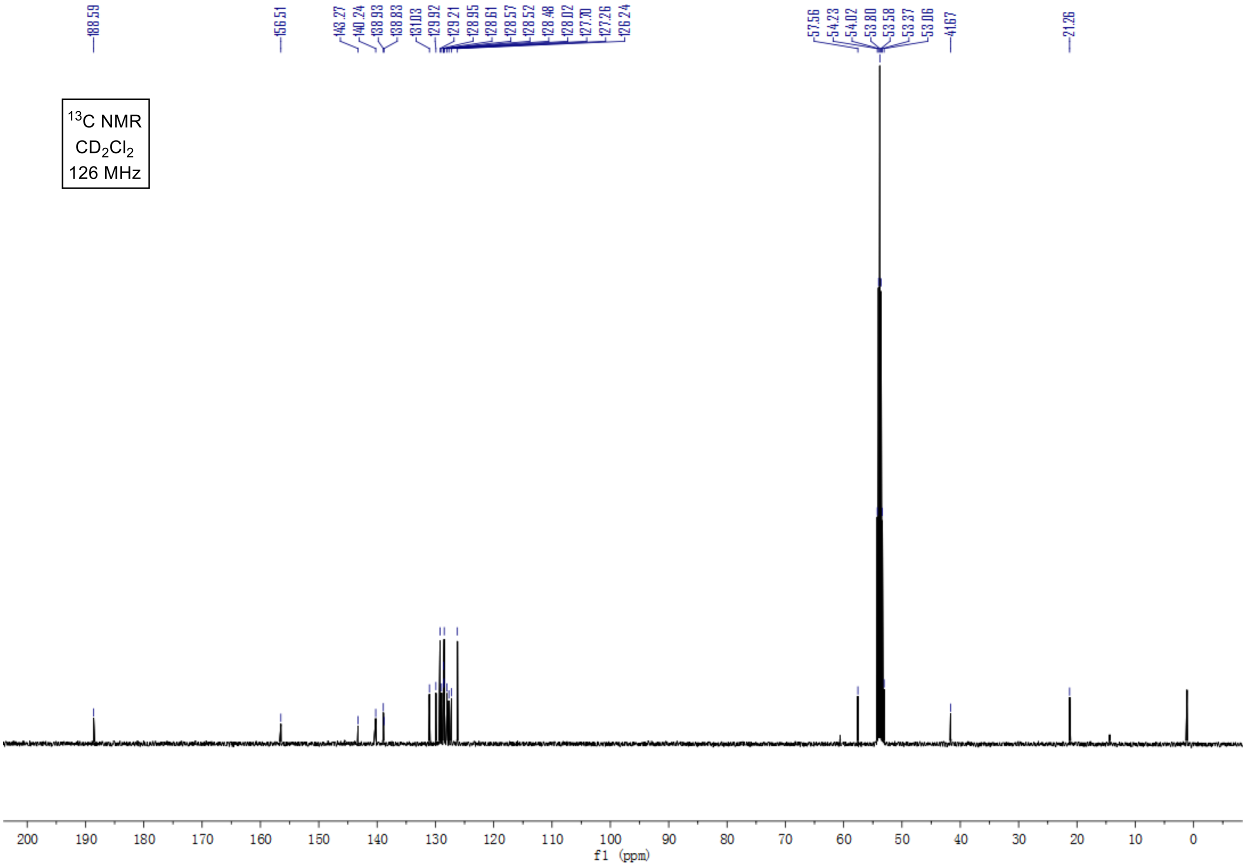

^{13}C NMR
CD_2Cl_2
126 MHz
f1 (ppm)

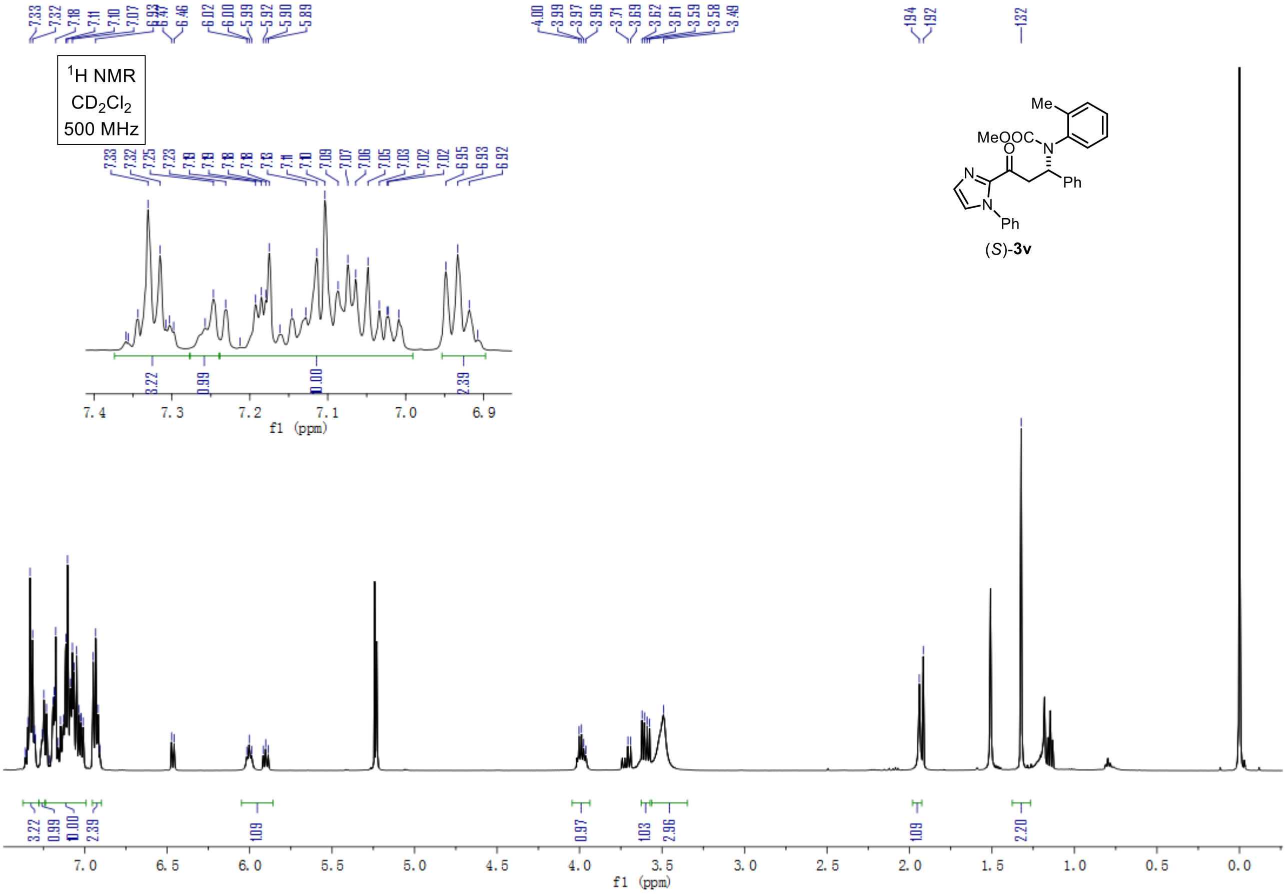

1H NMR
CD2Cl2
500 MHz
MeOOC
Me
N
O
N
N
Ph
Ph
(S)-3v
f1 (ppm)

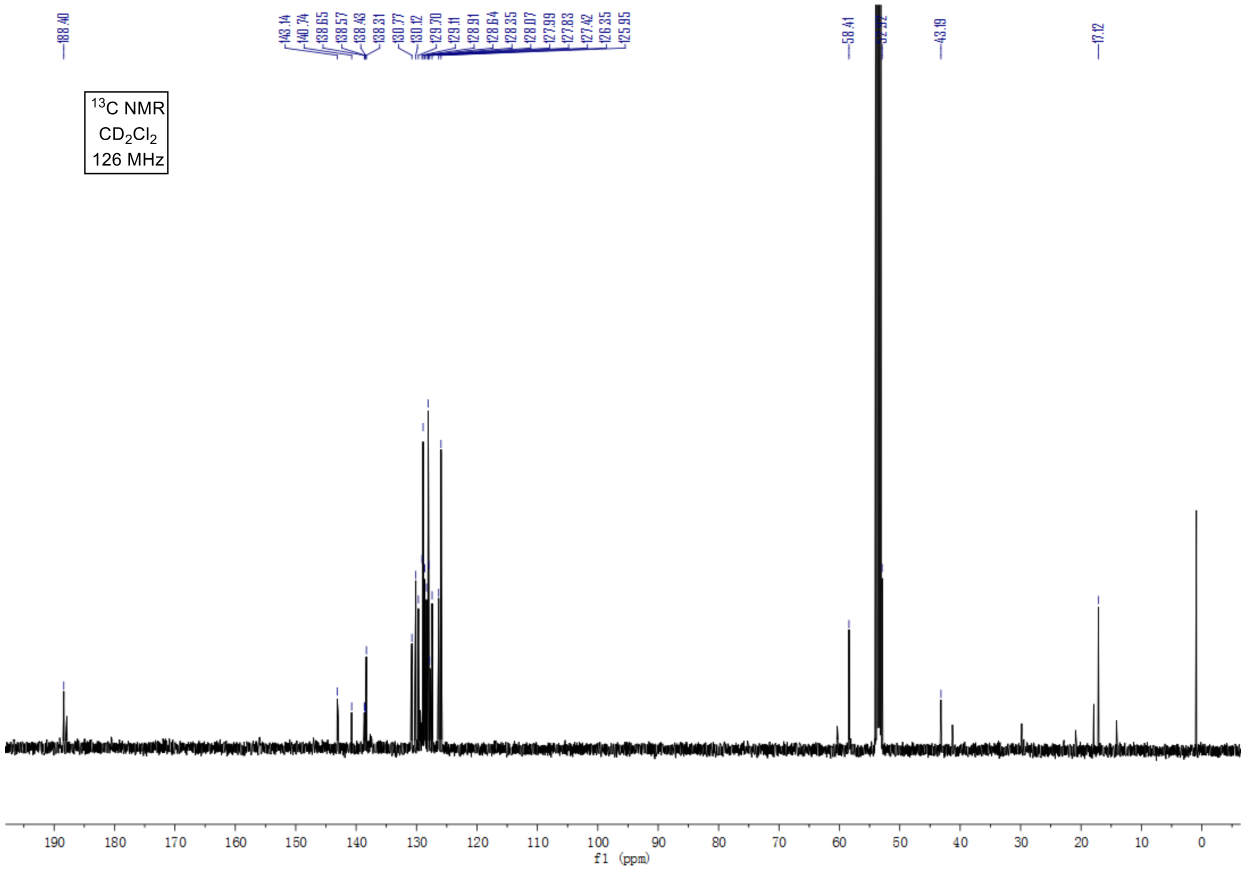

13C NMR
CD2Cl2
126 MHz
f1 (ppm)

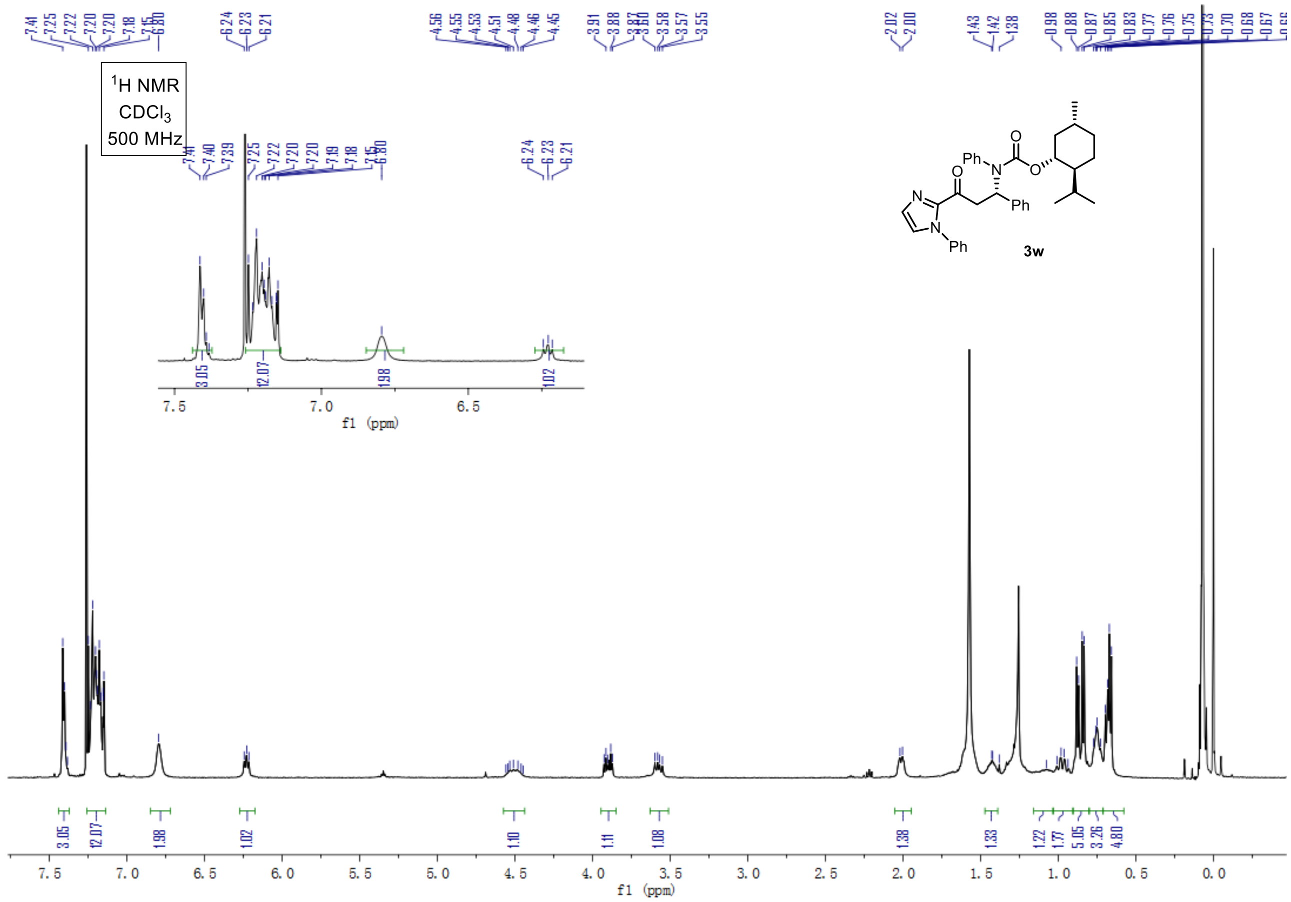
1H NMR
CDCl3
500 MHz
Ph
N
O
O
O
N
N
Ph
Ph
3w
f1 (ppm)

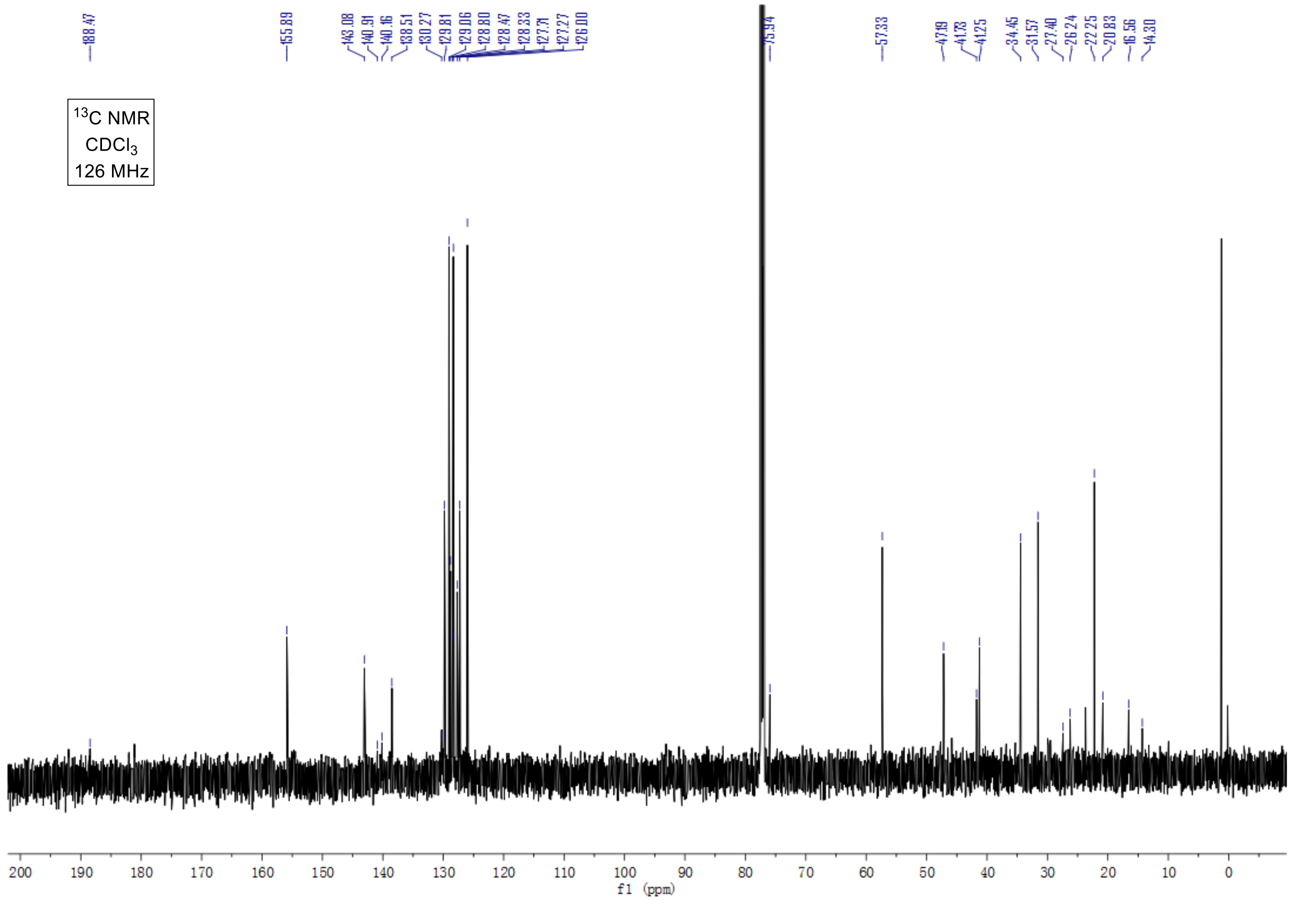
13C NMR
CDCl3
126 MHz
f1 (ppm)

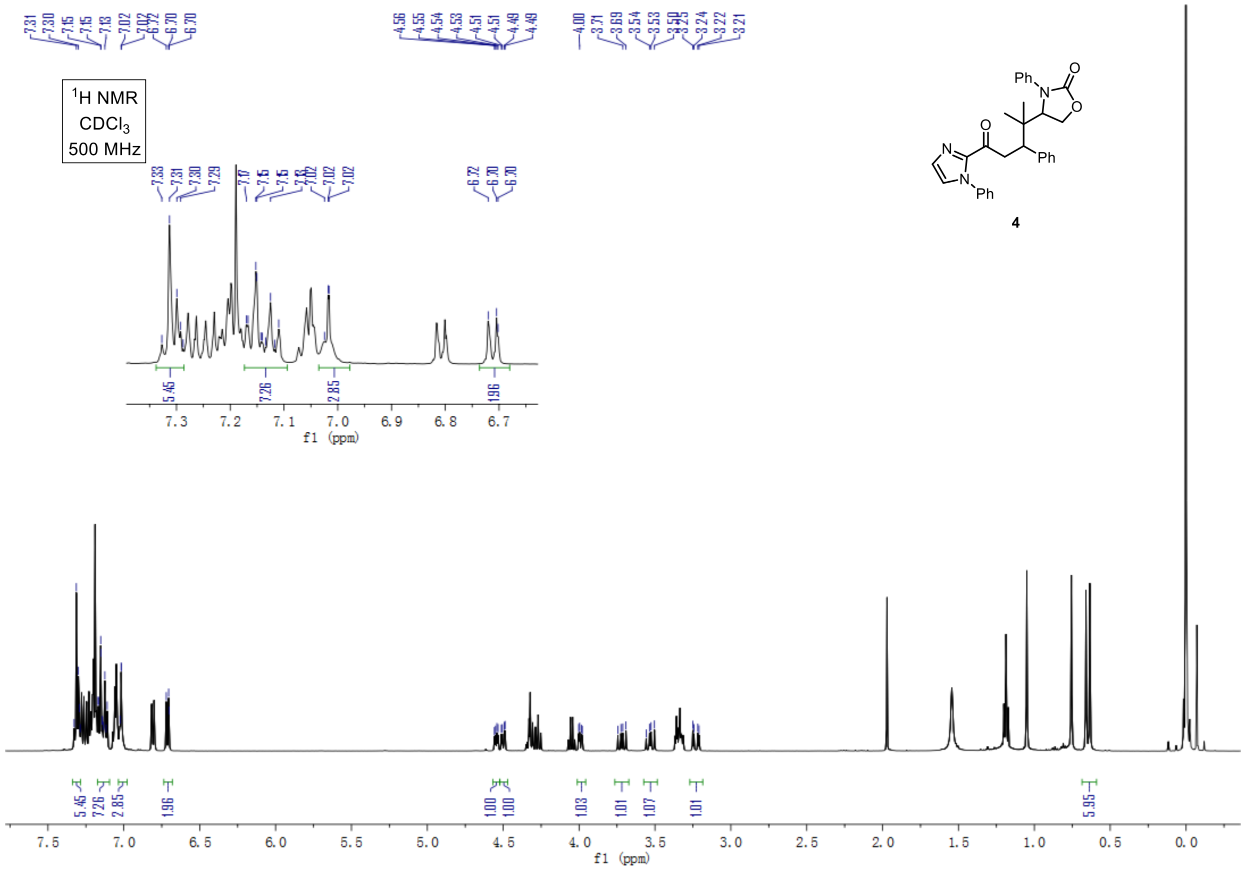

1H NMR
CDCl3
500 MHz
4
f1 (ppm)

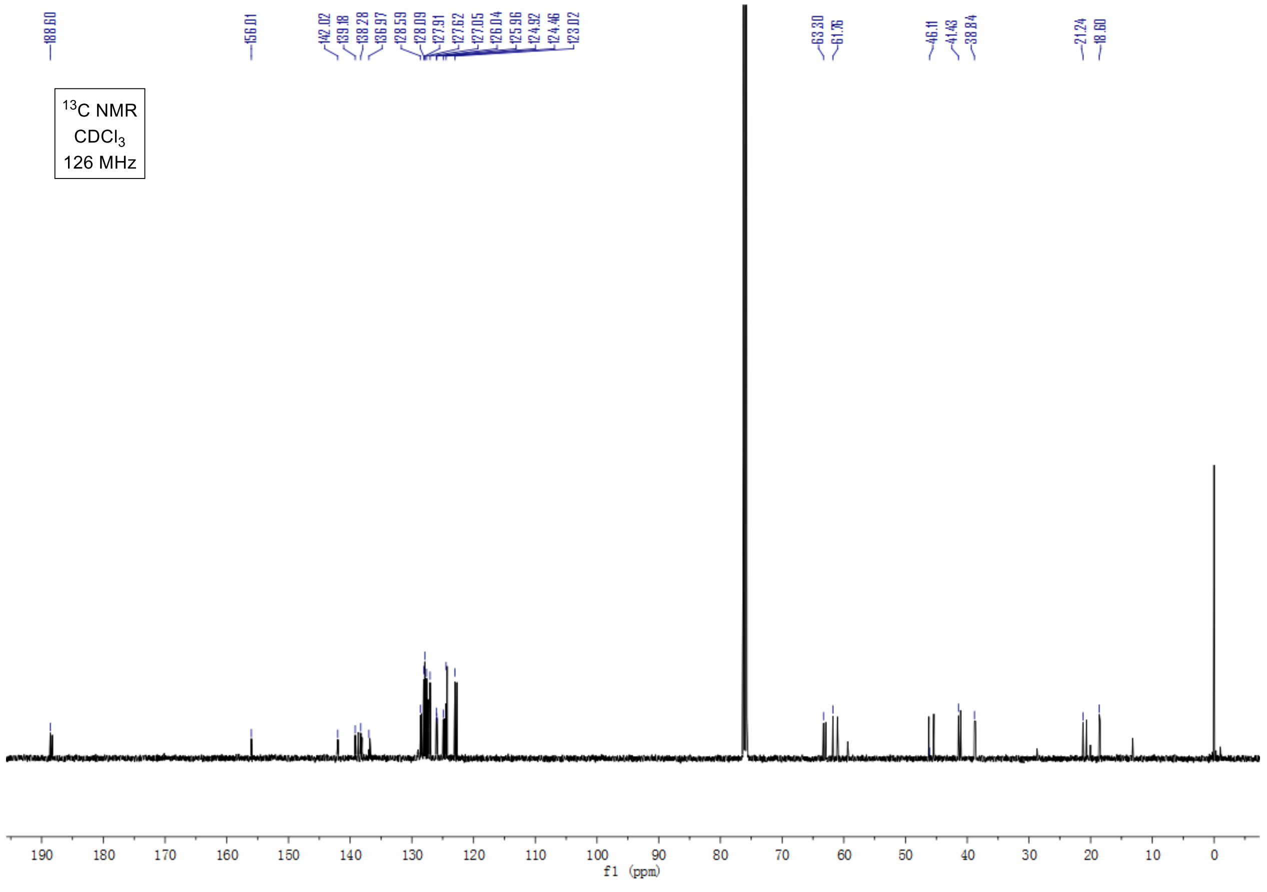

13C NMR
CDCl3
126 MHz
f1 (ppm)

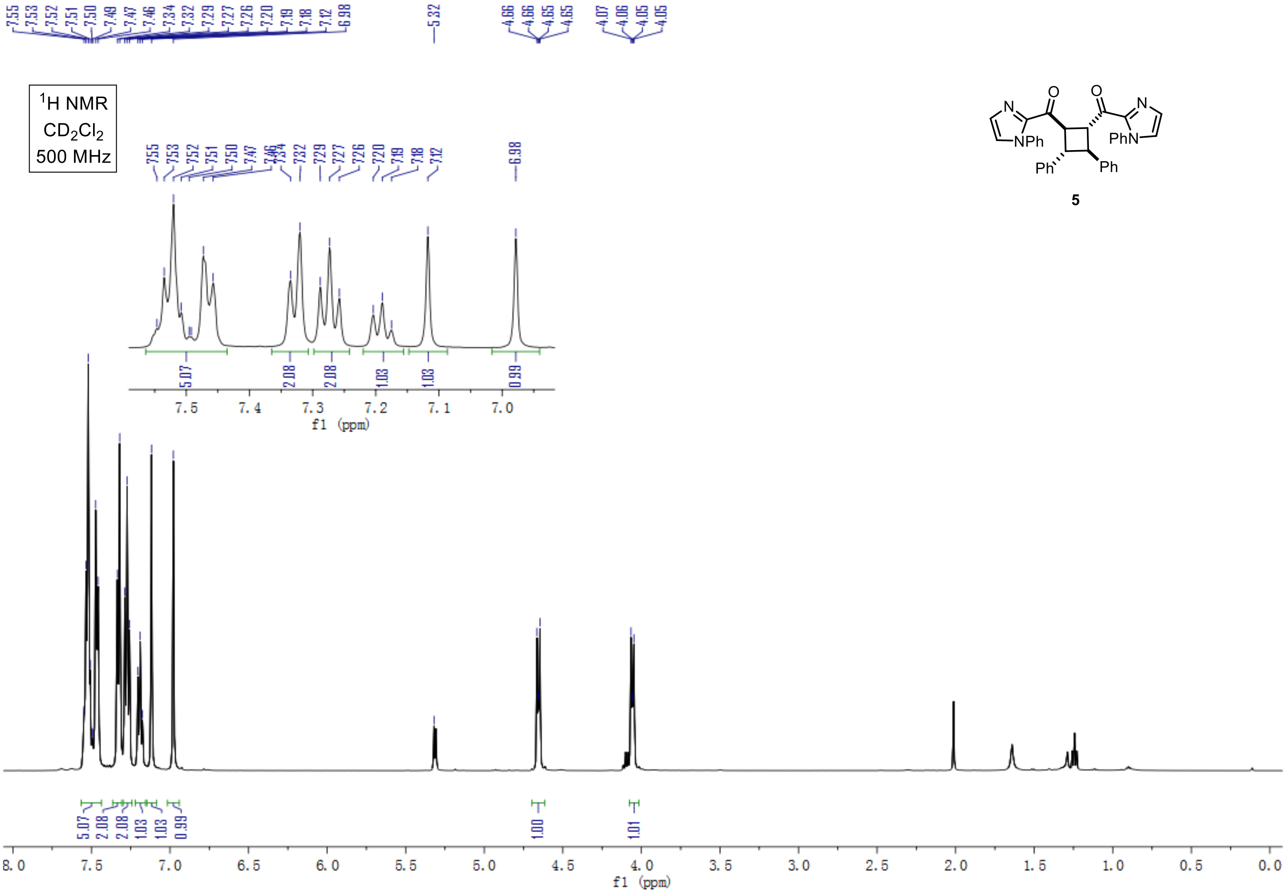

1H NMR
CD2Cl2
500 MHz
7.55 7.53 7.52 7.51 7.50 7.49 7.47 7.46 7.34 7.32 7.29 7.27 7.26 7.20 7.19 7.18 7.12 6.98
5.32
4.66 4.66 4.65 4.65
4.07 4.06 4.05 4.05
O
O
N
N
NPh
PhN
Ph
Ph
5
5.07 2.08 2.08 1.03 1.03 0.99
1.00
1.01
f1 (ppm)

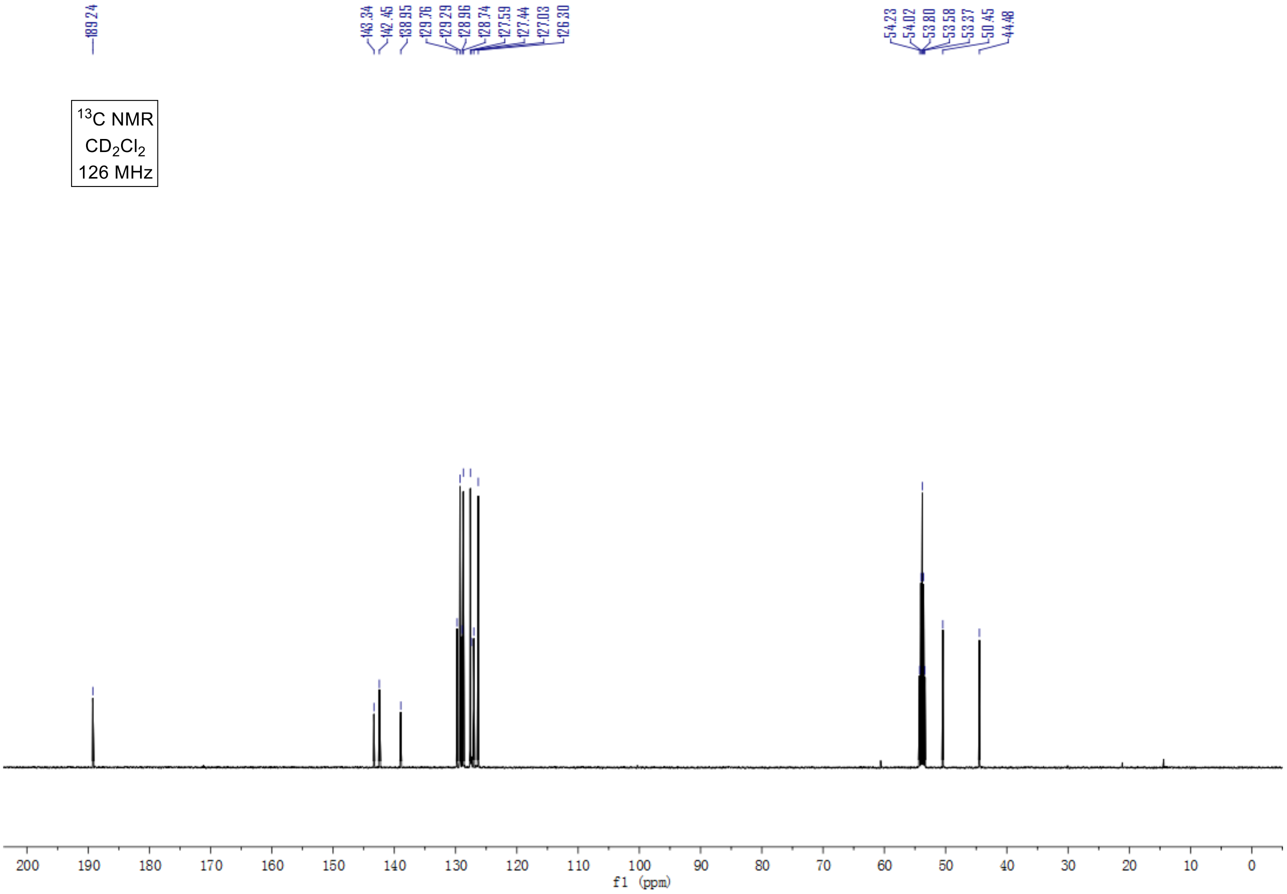

13C NMR
CD2Cl2
126 MHz
189.24
143.34 142.45 138.95 129.76 129.29 128.96 128.74 127.59 127.44 127.03 126.30
54.23 54.02 53.80 53.58 53.37 50.45 44.48
f1 (ppm)

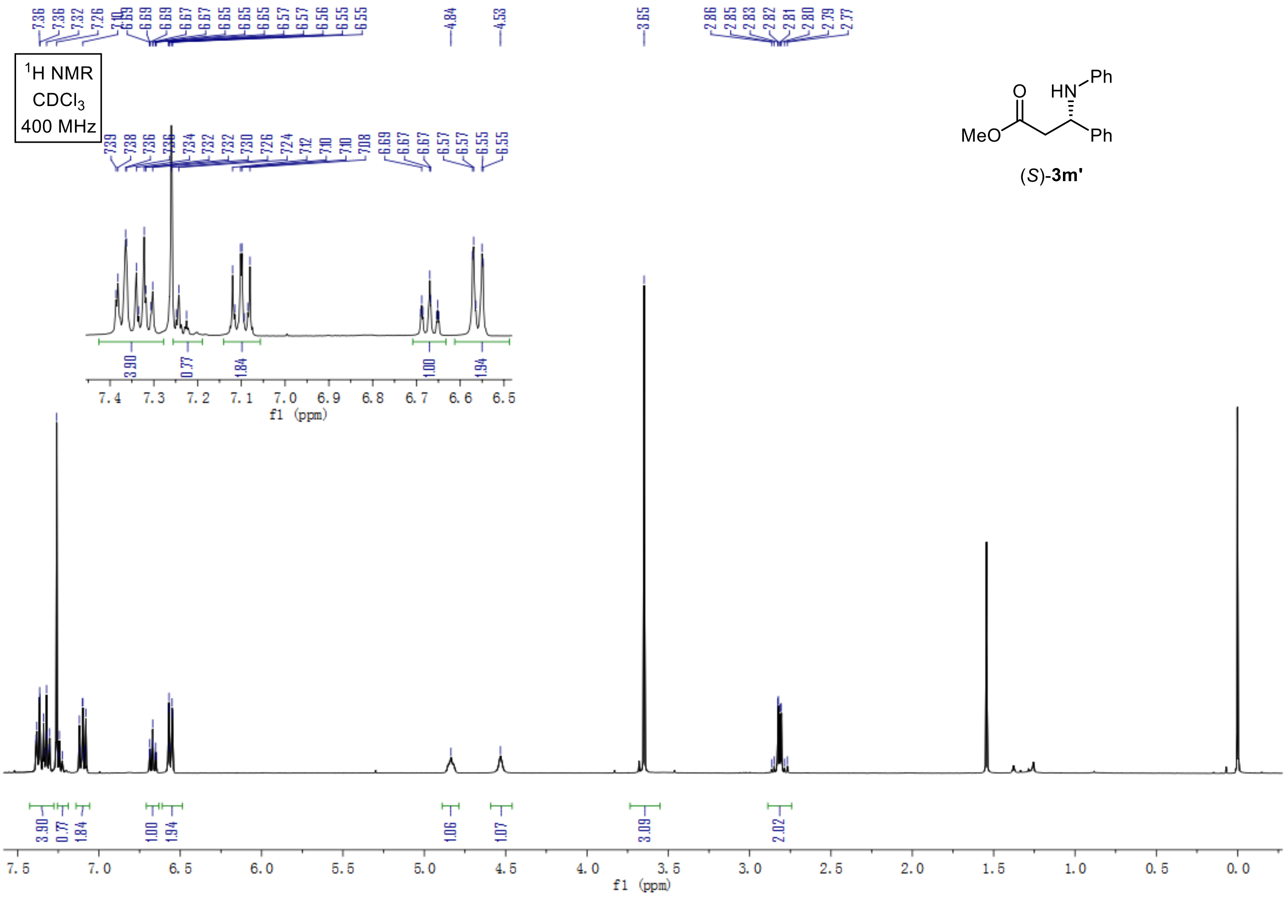

1H NMR
CDCl3
400 MHz
(S)-3m'
MeO
O
HN
Ph
Ph
f1 (ppm)
